# Small-molecule modulation of β-arrestins

**DOI:** 10.1038/s41586-026-10683-5

**Published:** 2026-06-24

**Authors:** Alem W. Kahsai, Natalia Pakharukova, Henry Y. Kwon, Kunal S. Shah, Caroline T. del Real, Bowie N. Shreiber, Jason G. Liang-Lin, Paul J. Shim, Mason A. Lee, Van A. Ngo, Allison M. Schwalb, Uyen Pham, Anand Chundi, Haoran Jiang, Emmanuel Flores-Espinoza, Samuel Liu, Preston C. Nibley, Dana K. Bassford, Hyunggu Hahn, Cal A. Kunzle, Brittany N. Thomas, Jihee Kim, Yang Zhou, Jialu Wang, Xingdong Zhang, Jeffrey S. Smith, Lindsay A. M. Rein, Alex R. B. Thomsen, Sudha K. Shenoy, Sudarshan Rajagopal, Lei Shi, Seungkirl Ahn, Howard A. Rockman, Ali Masoudi, Robert J. Lefkowitz

**Affiliations:** 1 Department of Medicine, Duke University Medical Center, Durham, NC USA; 2 Department of Surgery, Henry Ford Hospital, Detroit, MI USA; 3 Duke University School of Medicine, Duke University Medical Center, Durham, NC USA; 4 Cedars-Sinai Medical Center, Los Angeles, CA USA; 5 US Department of Energy, National Center for Computational Sciences, Oak Ridge National Laboratory, Oak Ridge, TN USA; 6 Department of Biochemistry, Duke University Medical Center, Durham, NC USA; 7 Department of Molecular Pathobiology, New York University College of Dentistry, New York, NY USA; 8 Department of Cell Biology, Duke University Medical Center, Durham, NC USA; 9 Departments of Dermatology, Massachusetts General Hospital and Harvard Medical School, Boston, MA USA; 10 Computational Chemistry and Molecular Biophysics Section, National Institute on Drug Abuse–Intramural Research Program, National Institutes of Health, Baltimore, MD USA; 11 Department of Chemistry, Duke University Medical Center, Durham, NC USA; 12 Howard Hughes Medical Institute, Duke University Medical Center, Durham, NC USA

**Keywords:** Cryoelectron microscopy, Mechanism of action, Chemical tools, Cryoelectron microscopy

## Abstract

β-Arrestins are multifunctional regulators of G-protein-coupled receptor (GPCR) signalling and orchestrate diverse downstream signalling events and physiological responses across the GPCR superfamily^1–3^. Although GPCR pharmacology has advanced to target orthosteric and allosteric sites, as well as G proteins and GPCR kinases, direct chemical tools to modulate β-arrestin activities have remained conspicuously absent. Here we report the identification of small-molecule inhibitors that selectively target β-arrestins and delineate their mechanism of action through integrated pharmacological, biochemical, biophysical and structural analyses. These inhibitors disrupt β-arrestin engagement with agonist-activated GPCRs, impairing desensitization, internalization and β-arrestin-dependent physiological functions while sparing G protein–receptor coupling. Cryo-electron microscopy, molecular dynamics simulations and structure-guided mutagenesis reveal that one modulator, Cmpd-5, engages a pocket within the central crest of β-arrestin1 formed by the middle, C and lariat loops, a critical receptor-binding interface, stabilizing a distinct conformation that is incompatible with full β-arrestin–receptor engagement. Together, these findings establish a mechanistic framework for β-arrestin modulation, reveal a novel allosteric site for structure-based drug design, and open new avenues for transducer-targeted, pathway-specific GPCR therapeutic agents.

## Abstract (web summary)

Integrated pharmacological, biochemical, biophysical and structural analyses of small-molecule β-arrestin inhibitors show how they block β-arrestin engagement with activated GPCRs, revealing their mechanism of action and uncovering a previously unrecognized allosteric regulatory site.

## Main

β-Arrestins are essential protein communication hubs that regulate ligand activation, signalling and trafficking of GPCRs^1–4^. GPCRs represent the most prominent family of transmembrane receptors in humans, encompassing more than 800 genes, and are the target of approximately one-third of all US Food and Drug Administration (FDA)-approved drugs^5^. As versatile adapter protein scaffolds, β-arrestins have a crucial role in GPCR desensitization, endocytosis and modulation of signalling pathways^3,4,6^. The mammalian arrestin family includes two widely expressed isoforms, β-arrestin1 (βARR1) and β-arrestin2 (βARR2) (also known as arrestin-2 and arrestin-3, respectively), along with two visual arrestins, arrestin-1 and arrestin-4, which are specialized for rod and cone photoreceptors^1,3,4,7^. Upon agonist activation, GPCRs interact with and activate heterotrimeric G proteins, triggering second messenger production and downstream signalling^1,3,5^. Subsequent phosphorylation of the agonist-activated receptors by GPCR kinases (GRKs), primarily within the carboxy-terminal tail, enables β-arrestins to bind to the receptor^1,2,8,9^. This binding sterically hinders further G-protein interaction, leading to receptor desensitization, attenuation of G-protein-mediated downstream signalling pathways, and receptor internalization via clathrin-coated pits^7,10,11^.

Beyond their traditional role in signalling termination, β-arrestins also function as versatile signal transduction units, participating independently or in concert with G proteins in diverse signalling cascades^3,12–15^. In this capacity, they scaffold and allosterically regulate diverse downstream effectors, facilitating interactions with signalling mediators including MAP kinases (MAPKs), the proto-oncogene kinase SRC, NF-κB, AKT (also known as protein kinase B) and MDM–p53, among others, as well as various components of the endocytic machinery^3,6,12,15,16^. Their involvement spans many biological processes, encompassing gene expression, metabolism, immune function, motility and apoptosis^3,4^. Consequently, dysregulated β-arrestin signalling is implicated in a broad spectrum of diseases, including cancer, inflammatory and autoimmune disorders, metabolic syndromes, cardiovascular conditions and neurological disorders^3,4,15,17–19^. Despite this central role, to our knowledge, no pharmacological agents directly target β-arrestins, in contrast to GPCR transducers such as G proteins and GRKs, for which there are many modulators^20–23^. As a result, studies of β-arrestins continue to rely on cumbersome genetic models and animal systems^4,24^. These limitations underscore the need for chemical strategies to probe and modulate β-arrestin activity. Here, we report the identification of the first small molecules that bind and inhibit β-arrestins, and illuminate their mechanism of action through integrated structural, computational and functional analyses. These findings establish a mechanistic framework for selectively modulating β-arrestin-mediated GPCR signalling.

## Identification of β-arrestin modulators

**Extended Data Fig. 1:**
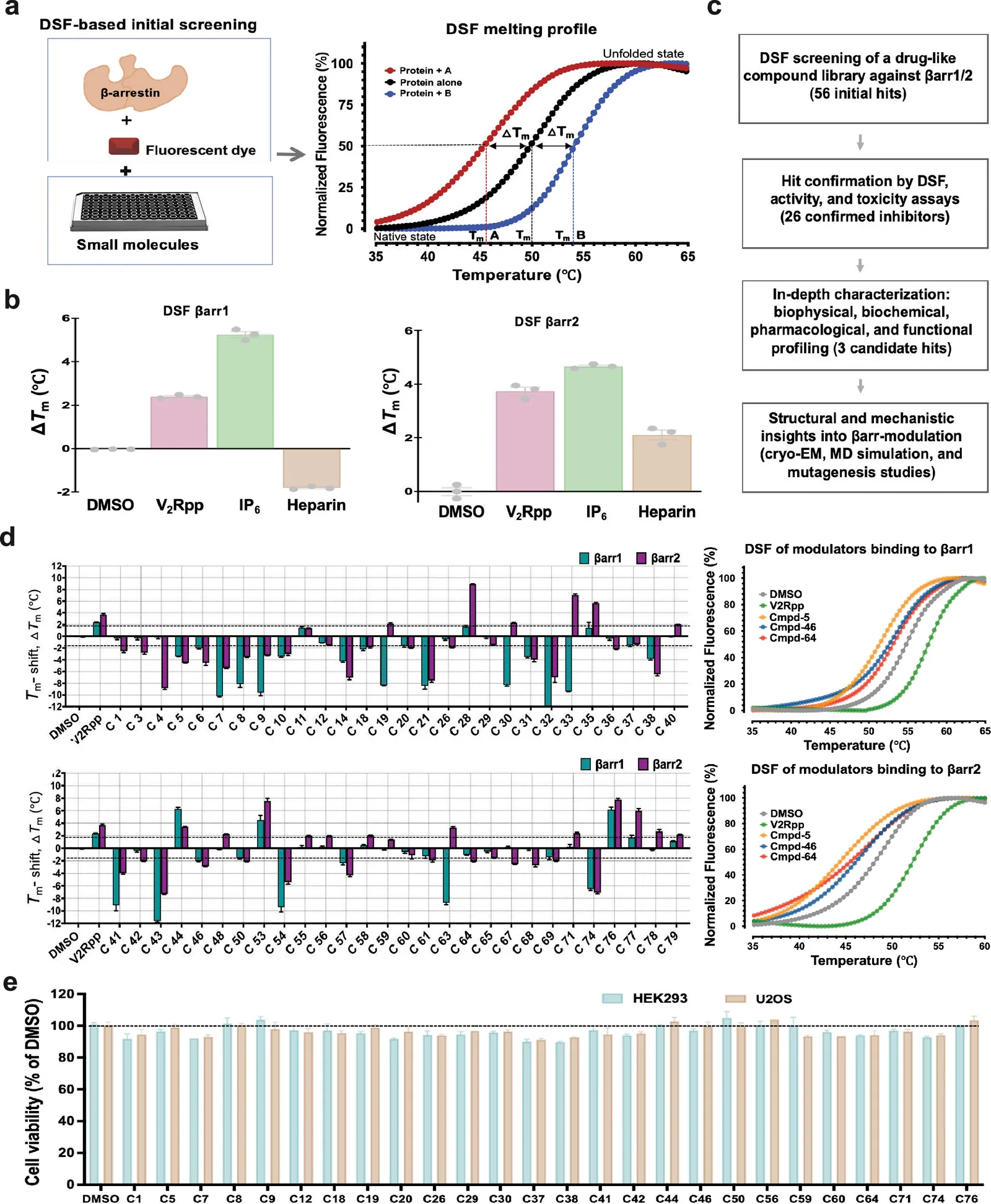
Differential scanning fluorimetry (DSF) platform for screening β-arrestin1/2 binding small molecules. **a**, Schematic of the DSF workflow for screening βarr1/2 binders. DSF of βarrs (5 µM) was performed using a fluorescent dye, with fluorescence measured as a function of temperature. Representative DSF profiles are shown as normalized fluorescence, with corresponding melting transitions (*T*_m_) and ligand-induced thermal shifts (Δ*T*_m_). **b**, DSF assay validation using known βarr ligands–V_2_Rpp, IP_6_, and heparin (50 µM each)–showing Δ*T*_m_ for βarr1 (left, ~54.5 °C) and βarr2 (right, ~49 °C). All three validation ligands are highly negatively charged, consistent with their known mode of βarr engagement. *T*_m_ shifts (|Δ*T*_m_ | ≥ 1.8 °C, dashed lines) denote significant binding, with the threshold informed by the Δ*T*_m_ of βarr1 upon heparin binding. Data are mean ± s.e.m., n = 3. **c**, Screening workflow outlining the multi-tiered process: initial DSF screening of a compound library (56 preliminary hits), followed by hit confirmation (26 validated binders), in-depth characterization yielding three candidate βarr modulators, and mechanistic and structural studies to elucidate modes of action. The screened NCI diversity set represents a collection of hundreds of thousands of structurally diverse compounds. Candidates such as C74 showed strong inhibition but were deprioritized due to poor solubility, physicochemical liabilities, or assay interference. **d**, DSF data for βarr1 and βarr2 (5 µM) in the presence of screened small molecules (100 µM, n = 3). Bar plots show Δ*T*_m_ for each compound, highlighting potential binders (Δ*T*_m_; both stabilizing and destabilizing shifts). Small molecules with |Δ*T*_m_ | ≥ 1.8 °C were considered binders. Right, representative DSF melting curves depict binding profiles of select modulators compared to controls. V_2_Rpp served as a positive control. Data are mean ± s.e.m., n = 3. **e**, Cell viability of HEK293 and U2OS cells treated with 50 µM of each compound or vehicle (DMSO) for 8 h, assessed by MTT assay. Viability is expressed as % relative to DMSO control (dashed line) (HEK293, light green; U2OS, beige). Data are mean ± s.e.m., n = 3.

**Extended Data Fig. 2:**
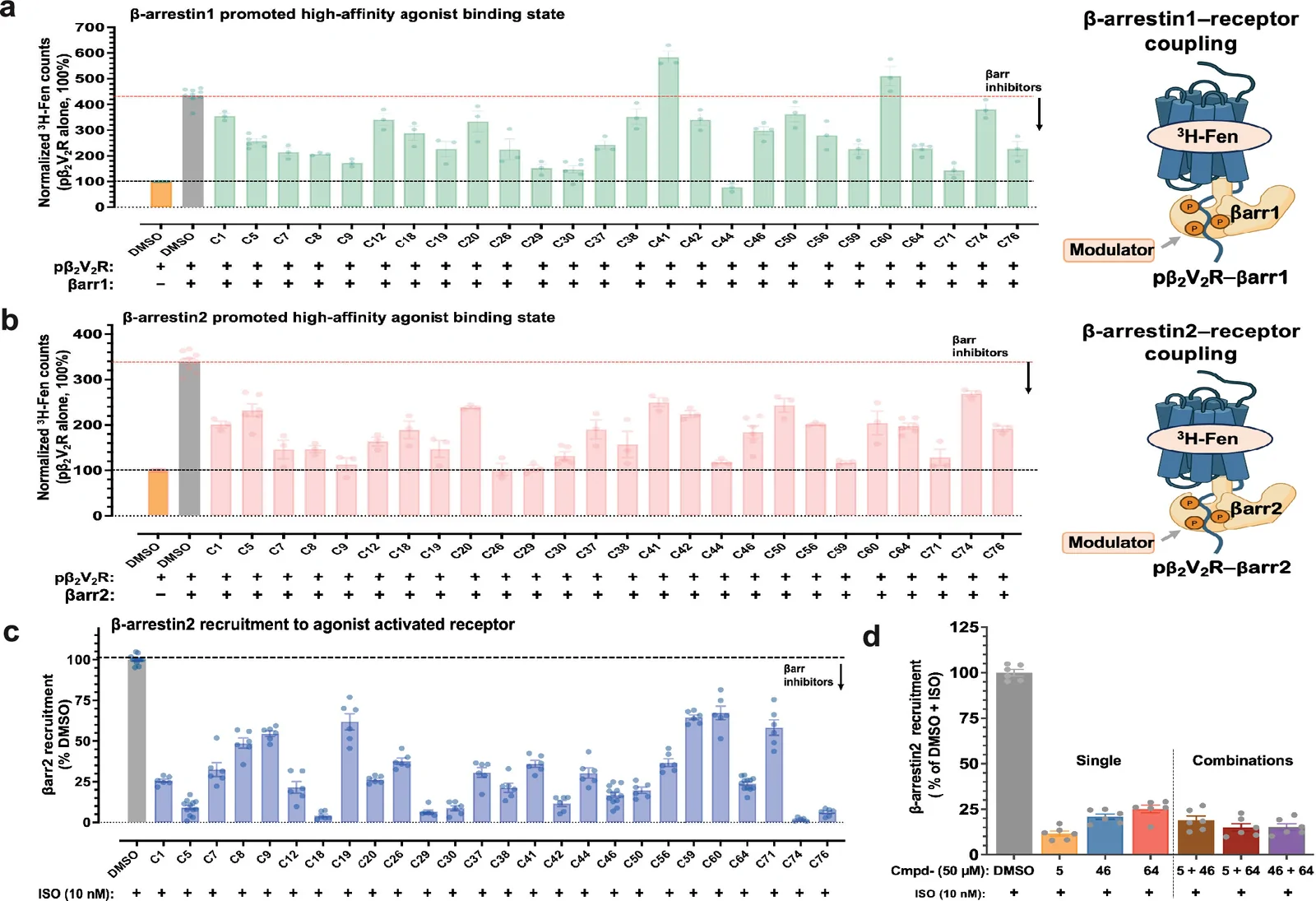
Characterization of putative β-arrestin modulators in cell-based and in vitro assays. **a**, Effect of modulators on βarr1–promoted high-affinity agonist binding to phosphorylated β_2_V_2_R. In vitro radioligand-binding assays were performed using Sf9 membranes containing p β_2_V_2_R and [3H]-Fen (6 nM), in the presence of βarr1, with or without modulators (100 µM). The chimeric β_2_V_2_R construct combines the β2-adrenergic receptor with the V_2_R C-terminal tail to drive robust βarr1/2 interaction. Binding increases with βarr coupling, as agonist association stabilizes the active receptor conformation, further promoting βarr engagement. The orange bar indicates pβ_2_V_2_R alone; the grey bar shows enhanced binding upon βarr1 addition (2 µM). The dashed red line marks this βarr1–stimulated binding level. Compounds with bars below this line are interpreted to impair βarr1–mediated receptor engagement. Right, schematic illustrating reciprocal stabilization of the phosphorylated receptor high-affinity state by βarrs, enhancing agonist binding. Data are mean ± s.e.m., n = 3. **b**, Effect of modulators on βarr2–promoted high-affinity agonist binding to phosphorylated β_2_V_2_R, performed as in a but with βarr2. Data are mean ± s.e.m., n = 3. **c**, Effect of βarr modulators on βarr2 recruitment to agonist-activated β_2_V_2_R. PathHunter U2OS cells expressing βarr2 and β_2_V_2_R were treated with each compound (50 µM, 30 min) before stimulation with isoproterenol (10 nM). Recruitment was quantified as % of DMSO control. Signals below the dashed line (agonist-only control) indicate inhibition of βarr2 recruitment. Arrows indicate direction of inhibition. Data are mean ± s.e.m., n = 5. Together with the binding data (panels a, b), these cell-based results helped prioritize compounds showing consistent activity, tractable chemical profiles, and suitability for downstream characterization. **d**, Additive inhibitory effects of βarr modulators on βarr2 recruitment. PathHunter βarr2 recruitment assay in U2OS cells expressing β_2_V_2_R and βarr2. Cells were pretreated with Cmpd-5, Cmpd-46, or Cmpd-64 (50 µM, 30 min) either alone or in pairwise combinations (25 µM each), then stimulated with the β-agonist isoproterenol (ISO; 10 nM). Recruitment was quantified as % of DMSO + ISO control. Single modulators reduced recruitment, and combinations produced comparable inhibition, consistent with dose additivity without synergy. Data are mean ± s.e.m., n = 6. Schematics in **a**,**b** created in BioRender; Kahsai, A. https://BioRender.com/pw680be (2026).

To identify small molecules that directly engage β-arrestins, we developed a multi-tiered discovery pipeline, beginning with differential scanning fluorimetry (DSF)^25,26^ to assess interactions with purified βARR1 and βARR2 (βARR1/2), followed by functional, biophysical, and structural analyses (Extended Data Fig. 1a–c). The DSF platform was validated using classic β-arrestin ligands (Extended Data Fig. 1b). Using DSF, we screened 3,500 chemically diverse compounds from the National Cancer Institute (NCI) diversity set, representing a collection of hundreds of thousands of compounds (Extended Data Fig. 1a,c). Preliminary hits were identified on the basis of thermal shifts (|Δ*T*_m_| ≥ 1.8 °C), a threshold guided by β-arrestin-binding controls (Extended Data Fig. 1a–d). These hits were further refined based on DSF reproducibility, toxicity filtering and functional screen data, yielding 26 putative inhibitors with favourable profiles (Extended Data Figs. 1d,e and 2a–c).

**Fig. 1:**
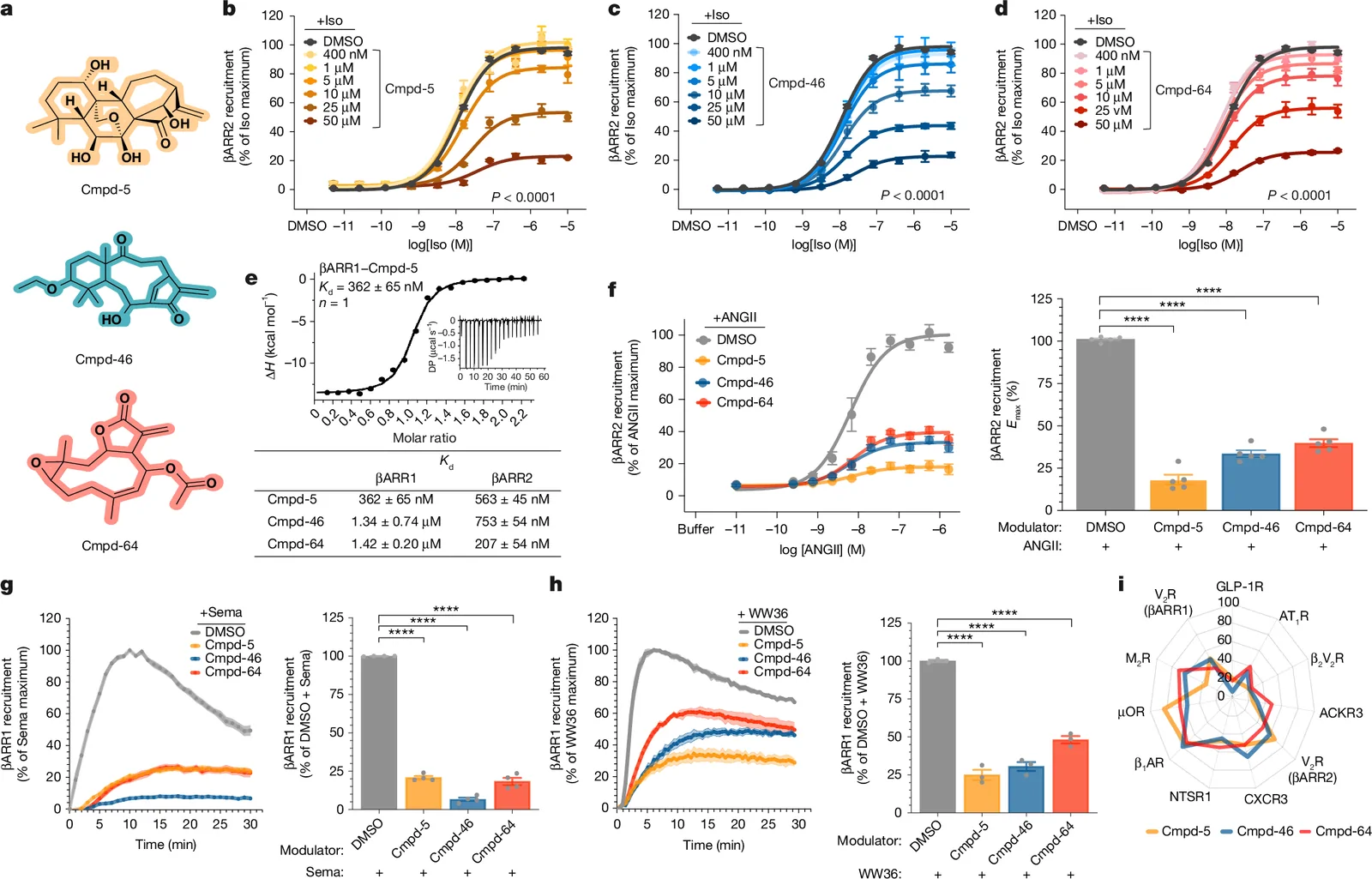
Identification and characterization of small-molecule β-arrestin modulators. **a**, Chemical structures of three validated β-arrestin modulators that act as inhibitors: Cmpd-5 (NSC 250682), Cmpd-46 (NSC 302979) and Cmpd-64 (NSC 22070). **b**–**d**, Dose-dependent inhibition of Iso-induced βARR2 recruitment to β_2_V_2_R, measured by PathHunter enzyme fragment complementation in U2OS cells co-expressing βARR2 (enzyme acceptor) and β_2_V_2_R (ProLink donor); βARR2 recruitment restores β-galactosidase activity, generating a chemiluminescent signal. Compounds were pre-incubated for 30 min before Iso stimulation, reducing maximum effect (*E*_max_) by 77.0 ± 1.2% (Cmpd-5) (**b**), 77.3 ± 1.0% (Cmpd-46) (**c**) and 74.4 ± 0.5% (Cmpd-64) (**d**) at 50 μM. **e**, ITC analysis of modulator binding to βARR1 and βARR2. Top, representative isotherm and raw injection heats (inset) for βARR1 with Cmpd-5 from one of three independent experiments, fit to a one-site model. Bottom, *K*_d_ values (mean ± s.e.m.) for all compounds with βARR1 or βARR2. **f**, Inhibition of ANGII-induced βARR2 recruitment to AT_1_R in CHO-K1 cells pretreated with modulators (50 μM, 30 min) before ANGII stimulation (1 μM, 1 h), measured by PathHunter assay. Right, bar graph shows *E*_max_ normalized to DMSO. **g**,**h**, Inhibition of βARR1 recruitment measured by NanoBiT complementation (SmBiT–βARR1; LgBiT–PM) in CRISPR βARR1/2-knockout HEK293 cells, following semaglutide (Sema) stimulation at GLP-1R (**g**) and WW36 stimulation at ACKR3 (**h**), with modulator pretreatment (50 μM). Left, luminescence traces; right, quantification based on peak signal relative to DMSO. **i**, Radar plot summarizing normalized βARR1/2 recruitment inhibition across the GPCR panel. Values are normalized to the vehicle and represent the mean inhibition per receptor–modulator pair. For GLP-1R, NTSR_1_, ACKR_3_, CXCR_3_, μOR, β_1_AR, M_2_R and V_2_R, βARR1 recruitment was measured; for AT_1_R, β_2_V_2_R and V_2_R, βARR2 recruitment was measured. Data are mean ± s.e.m.; *n* = 4 (**b**–**d**,**g**), *n* = 5 (**f**), *n* = 3 (**h**). One-way analysis of variance (ANOVA) with Dunnett’s post hoc analysis. *P < 0.05, **P < 0.01, ***P < 0.001, ****P < 0.0001; NS, not significant (*P* ≥ 0.05).

Among the functional screens, we used two complementary approaches to assess β-arrestin-dependent activity. First, we used a pharmacological assay to measure how candidate compounds affected the ability of βARR1/2 to engage and stabilize agonist-activated, phosphorylated receptor. To track this, we monitored the binding of [^3^H]-4-methoxyfenoterol (^3^H-Fen)—a radiolabelled β_2_-adrenergic receptor (β_2_AR) agonist whose occupancy increases allosterically with the coupling of β-arrestin^27–29^—using a chimeric pβ_2_V_2_R system in which the β_2_AR is fused to the C-terminal tail of the vasopressin receptor (V_2_R) to drive robust βARR1/2 engagement^7,28^ (Extended Data Fig. 2a,b). Here, a single-dose screen revealed that most modulators reduced β-arrestin coupling to pβ_2_V_2_R by at least 30% for at least one isoform (Extended Data Fig. 2a,b). To complement this pharmacological readout, we also evaluated β-arrestin responses in cells using a chemiluminescence-based βARR2 recruitment assay (PathHunter) to agonist-activated β_2_V_2_R. All 26 compounds inhibited isoproterenol (Iso)-induced βARR2 recruitment (Extended Data Fig. 2c). Based on these analyses, we prioritized three chemically distinct modulators, Cmpd-5 (also known as oridonin), Cmpd-46 and Cmpd-64, for their robust inhibition across orthogonal assays, tractable chemical properties and suitability for mechanistic characterization (Fig. 1a and Extended Data Fig. 1e). Other candidates that showed strong inhibition were deprioritized owing to poor solubility, physicochemical liabilities or assay interference. Notably, the three prioritized compounds each reduced β-arrestin-mediated high-affinity agonist binding by about 40%, consistent with their effects in the βARR2 recruitment assay (Extended Data Fig. 2a–c). Each also demonstrated robust, dose-dependent inhibition of Iso-induced βARR2 recruitment, with maximal reductions of 77.0% (Cmpd-5), 77.3% (Cmpd-46) and 74.4% (Cmpd-64) (Fig. 1b–d). To assess their functional interplay, we tested the compounds in combination in the same experiment. Pairwise treatments yielded inhibition of Iso-induced βARR2 recruitment comparable to those of individual modulators, indicating dose additivity without synergy (Extended Data Fig. 2d), consistent with non-occlusive mechanisms.

**Extended Data Fig. 3:**
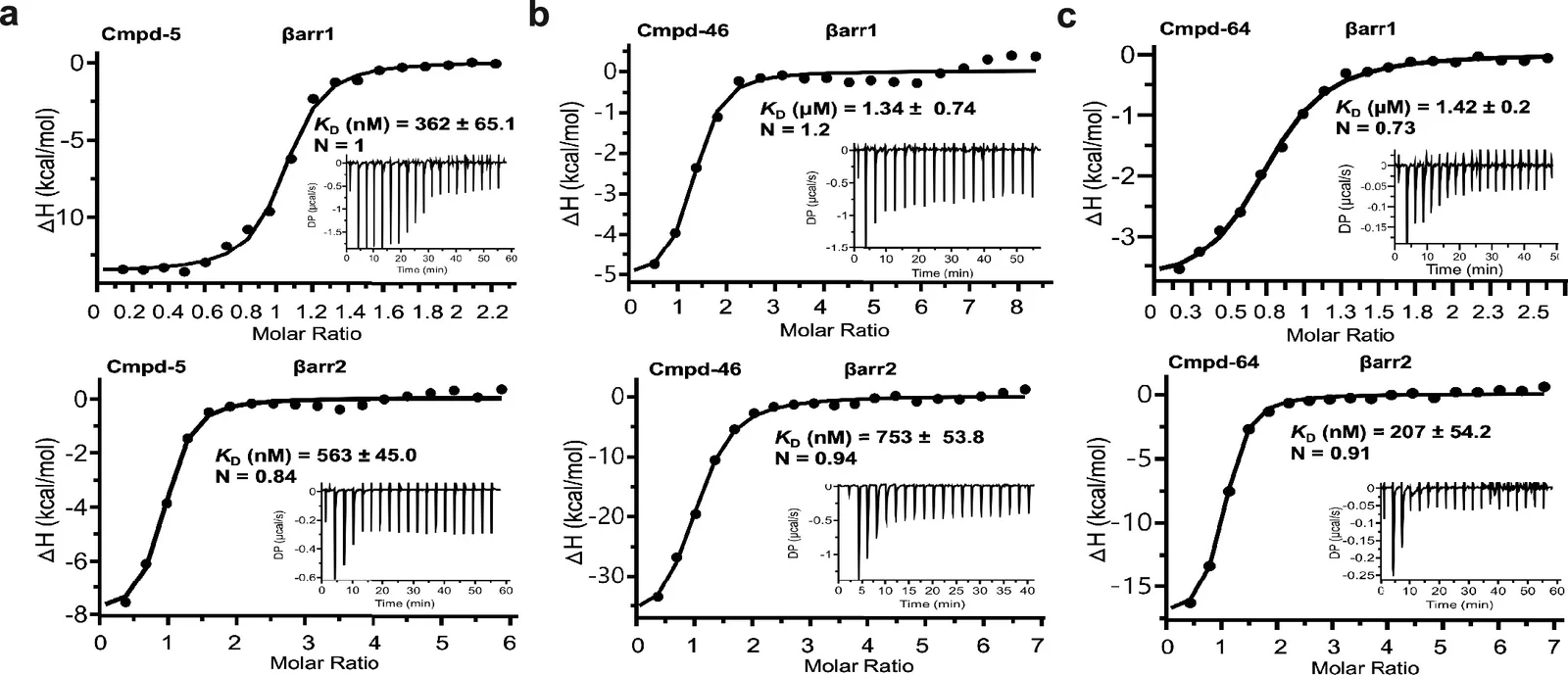
Isothermal titration calorimetry (ITC) analysis of modulator binding to βarr1 and βarr2. **a–c**, ITC isotherms for modulator binding to βarr1 (top) and βarr2 (bottom). **a**, Cmpd-5; **b**, Cmpd-46; **c**, Cmpd-64. Raw injection heats (insets) and fitted binding isotherms (solid lines) were modeled using a one-site independent binding model. Apparent Equilibrium dissociation constants (*K*_D_) and stoichiometry values (N) are shown as mean ± s.e.m., based on fits from independent measurements. Representative titration curves from 2–3 independent experiments are shown. Cmpd-5 bound both isoforms comparably; Cmpd-46 showed slight βarr2 preference, and Cmpd-64 exhibited ~6-fold selectivity for βarr2 over βarr1. Stoichiometry values for most interactions were consistent with 1:1 binding.

**Extended Data Fig. 4:**
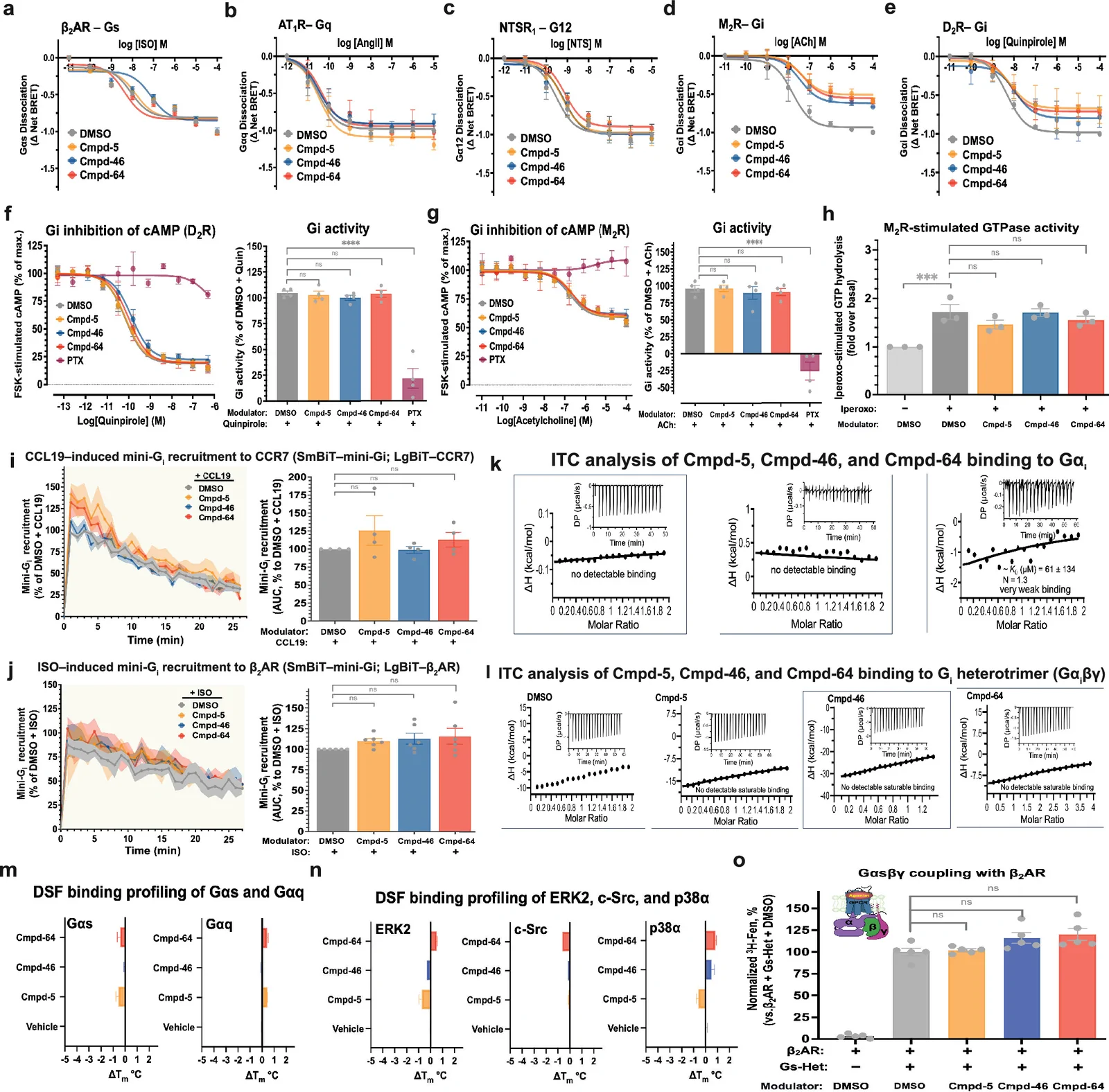
Selectivity assessment of β-arrestin modulators. **a**–**e**, TRUPATH BRET2 assays measuring GPCR-mediated Gα–Gβγ dissociation. HEK293T βarr1/2 KO cells expressing the indicated GPCR, Gα-RLuc8, Gβ and Gγ-GFP2 were stimulated with the respective agonists. Dissociation of Gα (RLuc8) from Gβγ (GFP2) was quantified as ΔNet BRET. GPCRs tested: a, β_2_AR–Gs (ISO); b, AT_1_R–Gq (Ang II); c, NTSR_1_–G12 (NTS); d, M_2_R–Gi (ACh); e, D_2_R–Gi (quinpirole). At M2R and D2R, reductions in maximal Gα-Gβγ dissociation were detected in the presence of modulators (mean fractional decrease vs DMSO: M_2_R − 0.43 [Cmpd-5], −0.32 [Cmpd-46], −0.38 [Cmpd-64]; D_2_R − 0.31 [Cmpd-5], −0.18 [Cmpd-46], −0.27 [Cmpd-64]). **f**, **g**, β-arrestin modulators do not inhibit Gi activity in mediating cAMP reduction at D_2_R or M_2_R. HEK293 cells expressing the GloSensor cAMP reporter with either D_2_R (f) or M_2_R (g) were pretreated with modulators (50 μM, 15 min) or vehicle (DMSO). Pertussis toxin (PTX; 100 ng/mL, overnight) served as a positive control for Gi blockade. Forskolin (FSK; 2 μM)-stimulated cAMP levels were normalized to the maximal FSK response per condition (left panels); Gi activity was quantified at the inhibitory plateau and normalized to DMSO plus agonist (right panels). **h**, β-arrestin modulators do not inhibit Gi-mediated GTP hydrolysis at M_2_R. GTPase activity was measured in vitro using the GTPase-Glo assay with HDL-reconstituted M_2_R (100 nM) pre-incubated with iperoxo (10 μM) or DMSO, followed by addition of purified Gi heterotrimer (500 nM), GTP (2.5 μM), and modulators (Cmpd-5, -46, or -64; 10 μM each) or DMSO. **i**, **j**, Agonist-induced recruitment of SmBiT-mini-Gi to LgBiT-tagged CCR7 (i) or β_2_AR (j) measured by NanoBiT complementation. Cells were stimulated with CCL19 (250 nM) or ISO (10 μM) in the presence of indicated modulators (50 μM). Time courses (left) and AUC quantification normalized to DMSO plus agonist (right) are shown. **k**, **l**, Binding profiling of βarr modulators with purified Gαi (k) and Gi heterotrimer (Gαiβγ) (l) by ITC. Representative binding isotherms and raw injection heats (insets) are shown for Cmpd-5, Cmpd-46 and Cmpd-64 (DMSO control shown for the heterotrimer). With Gαi, no detectable binding was observed for Cmpd-5 or Cmpd-46, whereas Cmpd-64 exhibited a weak, poorly defined interaction. With Gi heterotrimer, no evidence of saturable binding was observed, and thermograms were comparable to DMSO/buffer control with matched DMSO carrier. Thermodynamic parameters were obtained by fitting to a one-site binding model. **m**, **n**, DSF analysis of modulator binding to Gαs and Gαq (m) and ERK2, c-Src and p38α kinases (n). Δ*T*_m_ values are shown relative to vehicle controls. **o**, βarr modulators do not alter Gαsβγ heterotrimer-mediated high-affinity agonist binding to β_2_AR. Inset, schematic of reciprocal allosteric coupling between agonist-bound β_2_AR and Gαsβγ, whereby each enhances the binding affinity of the other. Schematic created in BioRender; Kahsai, A. https://BioRender.com/pw680be (2026). Bar graph, radioligand binding assays in Sf9 membranes expressing β_2_AR were performed using [^3^H]-Fen (4.3 nM) and purified Gαsβγ (200 nM) with or without modulators (Cmpd-5, -46 or -64; 100 µM). Gαsβγ enhanced agonist binding (grey), whereas modulators had no significant effect. Data are normalized to β_2_AR + Gαsβγ + DMSO. Data are mean ± s.e.m.; n = 3 (a–e), n = 4 (f, g), n = 3 (h), n = 4 (i), n = 5 (j), n = 2–3 (k–l); representative traces shown for k, l; n = 3 (m–n) and n = 5 (o). One-way ANOVA with Dunnett’s post hoc test; ****P < 0.0001 (f, g); ***P < 0.001 (h); ns, not significant.

To further probe direct interactions, we measured modulator binding to βARR1/2 by isothermal titration calorimetry (ITC), revealing dissociation constants (*K*_d_) in the high-nanomolar to low-micromolar range (Fig. 1e and Extended Data Fig. 3). Cmpd-5 bound each isoform similarly, Cmpd-46 showed modest βARR2 preference, and Cmpd-64 exhibited approximately sixfold selectivity for βARR2 over βARR1. After confirming direct binding to β-arrestins, we next tested whether the modulators engaged other transducers. Systematic profiling across the four canonical Gα subfamilies using the TruPath bioluminescence resonance energy transfer (BRET) biosensor platform revealed no appreciable effects at Gα_s_ (β_2_AR), Gα_q_ (angiotensin receptor (AT_1_R)) or Gα12 (neurotensin receptor (NTSR_1_)) (Extended Data Fig. 4a–c). At G_i_-coupled receptors, reductions in maximal Gα–Gβγ dissociation were detected at both muscarinic receptor (M_2_R) and D_2_ dopamine receptor (D_2_R) (Extended Data Fig. 4d,e). To determine whether these signals reflected direct G_i_ modulation, we turned to the classical measure of G_i_ activity, G_i_-mediated inhibition of adenylyl cyclase, the functional readout through which inhibitory G proteins were originally discovered. None of the modulators altered agonist-induced cAMP reduction at D_2_R or M_2_R, whereas pertussis toxin, a well-established G_i_ inhibitor, abolished this response entirely (Extended Data Fig. 4f,g). Similarly, using M_2_R reconstituted into lipid nanodiscs and purified G_i_ heterotrimer, we measured iperoxo-stimulated GTP hydrolysis, which was unaffected by any β-arrestin modulator (Extended Data Fig. 4h), providing direct in vitro evidence against G_i_ inhibition. Consistent with these results, agonist-induced recruitment of mini-G_i_ to CCR7 and β_2_AR remained similar to controls, indicating that receptor–G protein coupling, which proceeds primarily through engagement of the α5 helix of Gα_i_, was similarly preserved (Extended Data Fig. 4i,j). Direct interaction measurements by ITC also revealed no detectable binding of the modulators to purified Gα_i_ or the G_i_ heterotrimer (Extended Data Fig. 4k,l), in clear contrast to the saturable, well-defined interactions observed with β-arrestins (Fig. 1e and Extended Data Fig. 3). Beyond G_i_, DSF profiling across Gα_s_, Gα_q_ and β-arrestin-associated effector proteins (ERK2, SRC and p38α) similarly revealed no detectable binding, and none of the modulators perturbed Gα_s_βγ-promoted high-affinity agonist binding to β_2_AR (Extended Data Fig. 4m–o). Collectively, across multiple experimental paradigms spanning cellular, cell-free and biophysical approaches, the modulators selectively engage β-arrestins and do not inhibit G_i_ activity. The effects observed in the TruPath assay thus appear to be assay-specific and should be interpreted with caution.

## β-Arrestin modulators inhibit β-arrestin recruitment

**Extended Data Fig. 5:**
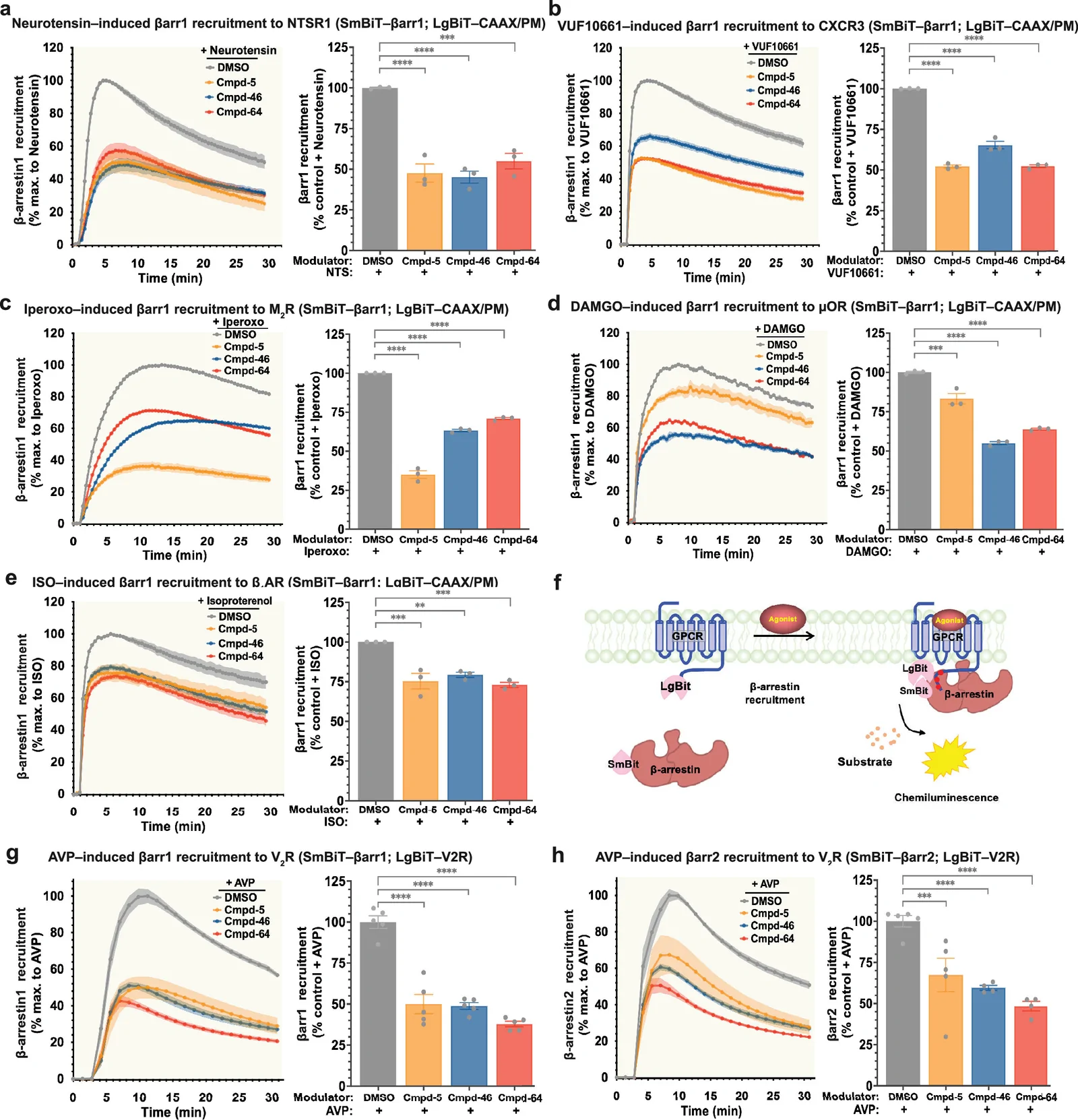
βarr modulators broadly inhibit βarr1/2 recruitment across a panel of GPCRs. **a**–**e**, Time-course and quantification of βarr1 recruitment to NTSR_1_ (a, neurotensin, 1 μM), CXCR_3_ (b, VUF10661, 10 μM), M_2_R (c, iperoxo, 1 μM), μOR (d, DAMGO, 10 μM), and β_1_AR (e, isoproterenol, 10 μM), measured in CRISPR βarr1/2-knockout HEK293 cells using NanoBiT (SmBiT–βarr1, LgBiT–CAAX). **f**, Schematic of the NanoBiT βarr recruitment assay. **g**, **h**, Inhibition of βarr1 (g) and βarr2 (h) recruitment to V2R upon AVP stimulation, using NanoBiT complementation with LgBiT–V2R and SmBiT–βarr1 or –βarr2. Modulators were applied at 50 μM for 30 min prior to agonist addition (100 nM AVP). For all panels: bar graphs represent peak recruitment normalized to vehicle (DMSO); data are mean ± s.e.m., n = 3 (a–e), n = 4–5 (g, h) biologically independent replicates. One-way ANOVA with Dunnett’s post hoc analysis; **P < 0.01; ***P < 0.001; ****P < 0.0001.

To assess the broader applicability of the β-arrestin modulators beyond the β_2_V_2_R model system, we evaluated their effects on β-arrestin recruitment across a diverse panel of GPCRs spanning distinct receptor families and coupling paradigms, encompassing receptors with tight, stable β-arrestin engagement as well as those with transient or intermediate interactions, including receptors that recruit β-arrestins through intracellular loop 3 (ICL3) and atypical chemokine receptors with β-arrestin-biased signalling (Fig. 1f–i and Extended Data Fig. 5). This panel comprised AT_1_R, V_2_R, chemokine receptor (CXCR_3_), NTSR_1_, β_1_AR, μ-opioid receptor (μOR) and M_2_R, as well as the β-arrestin-biased atypical chemokine receptor ACKR3 (also known as CXCR7) and the incretin receptor GLP-1R, a class B1 GPCR with clinical relevance in metabolic disorders. β-Arrestin recruitment was quantified using PathHunter enzyme fragment complementation for AT_1_R and NanoBiT complementation for all others, in CRISPR–Cas9 βARR1/2-knockout HEK293 cells (Extended Data Fig. 5). Across this panel, all three modulators produced robust but receptor-dependent inhibition of βARR1/2 recruitment (35–94% inhibition; Fig. 1f–i and Extended Data Fig. 5). GLP-1R and AT_1_R showed the greatest reductions (93% and 82%, respectively), resembling that of β_2_V_2_R (Fig. 1b–d,f,g). Representative inhibition profiles are shown for βARR2 at AT_1_R (using the agonist angiotensin II (ANGII)), βARR1 at ACKR_3_ (using WW36) and GLP-1R stimulated with semaglutide, a clinically used metabolic agonist (Fig. 1f–h). Although full blockade was not observed, particularly among transient, class A-like receptors, this is likely to reflect modulator-induced stabilization of a β-arrestin conformation that limits productive coupling rather than abolishing constitutive β-arrestin–receptor engagement. A radar plot (Fig. 1i) summarizes normalized inhibition across all receptors, highlighting the broad yet mechanistically distinct actions of the modulators across GPCR classes. Collectively, these findings demonstrate the broad applicability of the modulators as chemical probes to explore β-arrestin-dependent signalling.

## Inhibition of desensitization and internalization

**Fig. 2:**
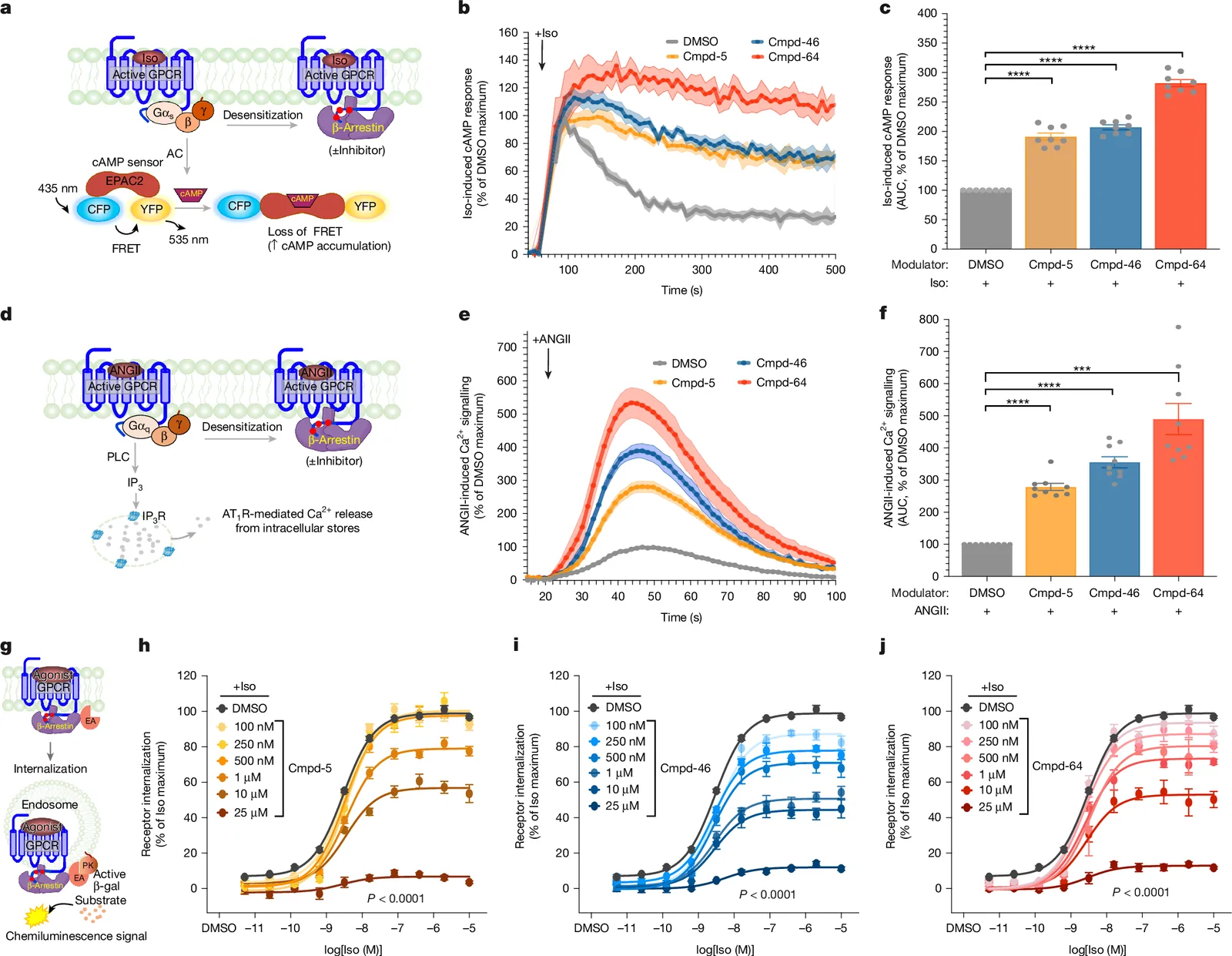
β-Arrestin modulators reduce agonist-induced receptor desensitization and internalization. **a**, Schematic of the Epac2 FRET cAMP biosensor (CFP–Epac2–YFP), in which cAMP binding increases the CFP–YFP distance, reducing FRET efficiency. AC, adenylyl cyclase. **b**, Kinetic profiles from HEK293 cells stably expressing β_2_AR and the cAMP sensor. Cells were pretreated with modulators (40 μM, 5 min) before Iso (10 μM) stimulation. **c**, Area under the curve (AUC) quantification of FRET responses. Modulators increased Iso-induced cAMP accumulation to 191% (Cmpd-5), 207% (Cmpd-46) and 282% (Cmpd-64) relative to DMSO (set to 100%). **d**, Schematic of AT_1_R-mediated calcium mobilization via the Gα_q_–PLC–inositol 1,4,5-trisphosphate (IP_3_) pathway. IP_3_R, inositol 1,4,5-trisphosphate receptor. **e**, Real-time Ca^2+^ signalling in HEK293 cells stably expressing AT_1_R. Cells were pretreated with modulators (10 μM, 30 min) before ANGII (30 pM) stimulation. **f**, AUC quantification showing enhanced Ca^2+^ responses by 279% (Cmpd-5), 356% (Cmpd-46) and 490% (Cmpd-64) relative to DMSO (set to 100%). **g**, Schematic of the PathHunter β-gal complementation internalization assay. Agonist-induced βARR2 (enzyme acceptor (EA)-tagged) recruitment drives internalization of untagged GPCRs into ProLink (PK)-tagged endosomes, reconstituting β-gal activity and generating luminescence. The assay was performed in U2OS cells expressing βARR2- and endosome-targeted reporters. **h**–**j**, Modulators dose-dependently inhibit Iso-induced β_2_V_2_R internalization. Pretreatment with modulators (30 min) followed by Iso stimulation reduced *E*_max_ by 93.2 ± 1.0% (Cmpd-5) (**h**), 88.0 ± 0.8% (Cmpd-46) (**i**) and 87.1 ± 0.8% (Cmpd-64) (**j**) at 25 μM. Data are mean ± s.e.m.; *n* = 8 (**b**,**c**,**e**,**f**) and *n* = 3 (**h**–**j**). One-way ANOVA with Dunnett’s test.

**Extended Data Fig. 6:**
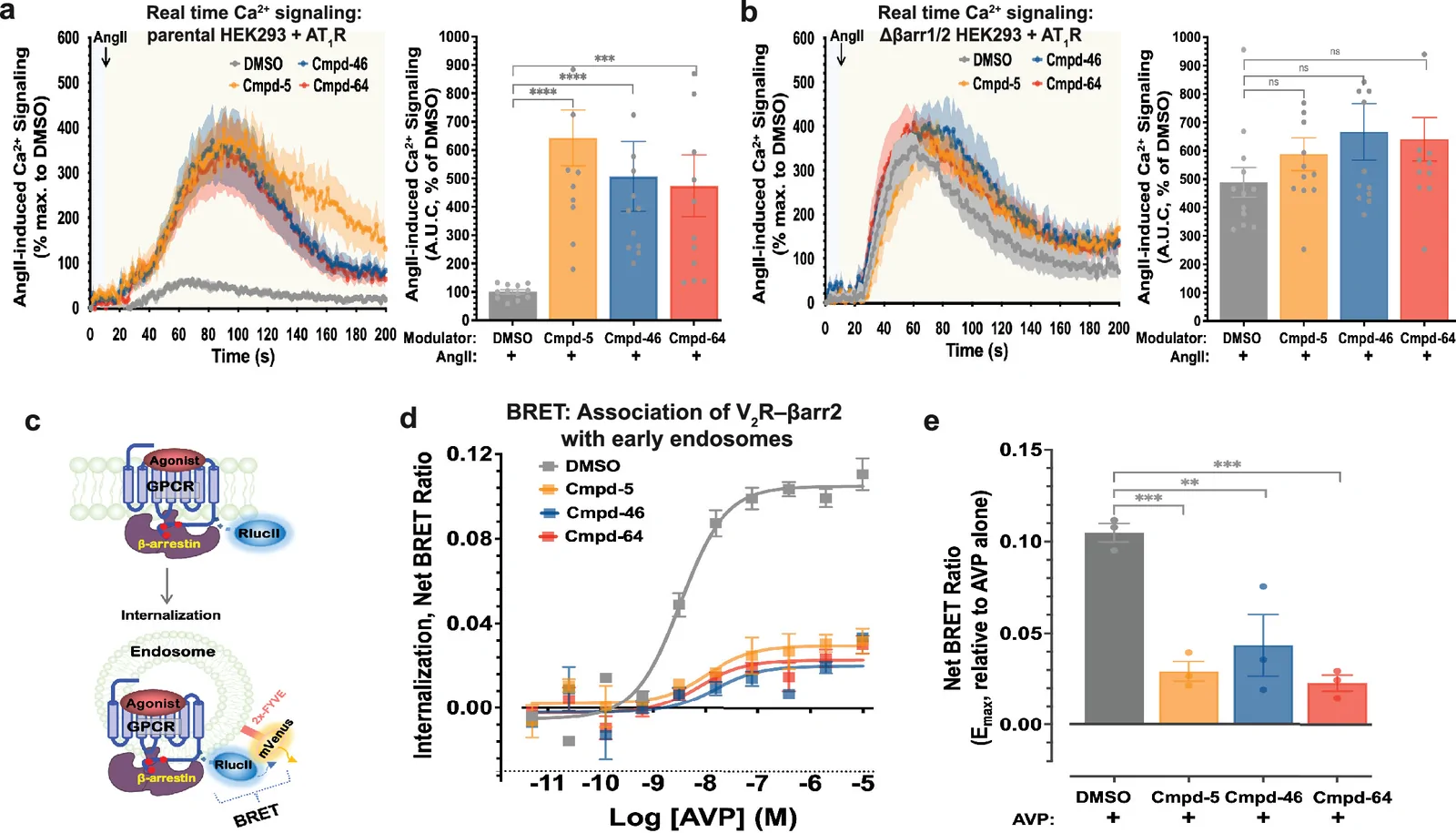
β-arrestin modulators reduce agonist-induced receptor desensitization and internalization. **a**, **b**, Real-time Ca^2+^ signaling in HEK293 cells transiently expressing AT_1_R: parental (a) or CRISPR βarr1/2-knockout (b). Cells were treated with modulators (10 μM, 30 min) and stimulated with AngII (120 pM). Right panels: AUC quantification normalized to AngII alone (100%). In parental HEK293 cells, βarr modulators increased AngII-stimulated Ca^2+^ mobilization by 4- to 5-fold relative to AngII alone, consistent with inhibition of βarr-mediated desensitization. In βarr1/2-knockout cells, no such potentiation was observed, confirming βarr dependence of the modulatory effect. **c**, Schematic of the BRET-based assay for monitoring GPCR association with early endosomes. Upon AVP stimulation, V_2_R–RlucII and βarr2 are internalized, bringing RlucII into proximity with the endosome-localized 2xFYVE–mVenus, resulting in increased BRET. Bioluminescence resonance energy transfer (BRET) between V2R–RlucII and 2xFYVE–mVenus was used to monitor receptor trafficking to early endosomes. **d**, HEK293 cells transiently expressing V_2_R–RlucII and 2xFYVE–mVenus were pretreated with βarr modulators (40 μM, 30 min), followed by stimulation with increasing concentrations of AVP. Modulators attenuated the net BRET ratio, indicating reduced receptor–endosome association. **e**, Quantification of Emax values relative to AVP + DMSO controls. Data are mean ± s.e.m.; n = 8 for a, b and n = 3 for d, e. One-way ANOVA with Dunnett’s test; ns, not significant; ***P < 0.001; ****P < 0.0001.

Prolonged GPCR activation attenuates agonist response, partly owing to β-arrestin binding to phosphorylated receptors, which uncouples them from G proteins and promotes internalization^2,3,10^. To examine how β-arrestin modulators influence this process, we used live-cell biosensors to monitor real-time second messenger dynamics, focusing on cAMP and Ca^2+^. Using a Förster resonance energy transfer (FRET)-based cAMP biosensor (ICUE2; CFP–Epac2–YFP)^30^ in HEK293 cells stably expressing β_2_AR (Fig. 2a), we found that all three modulators inhibited β_2_AR desensitization, sustaining cAMP signalling and enhancing agonist-induced cAMP accumulation relative to dimethyl sulfoxide (DMSO) control (set to 100%) (Fig. 2b,c). To determine whether this effect extended to a Gα_q_-coupled receptor, we examined ANGII-induced calcium mobilization in HEK293 cells stably expressing AT_1_R^31^ (Fig. 2d). Consistent with the results for β_2_AR, the modulators significantly inhibited AT_1_R desensitization, enhancing Gα_q_-mediated Ca^2+^ signalling, a readout sensitive to changes in β-arrestin-mediated desensitization (Fig. 2e,f). To confirm that these effects were β-arrestin-dependent, we performed Ca^2+^ signalling assays in parental HEK293 cells and CRISPR–Cas9-generated βARR1/2 knockouts that transiently expressed AT_1_R (Extended Data Fig. 6a,b). In parental cells, modulators increased ANGII-stimulated Ca^2+^ mobilization by four to fivefold, whereas this effect was absent in βARR1/2-knockout cells, indicating that the modulatory effect is β-arrestin-dependent (Extended Data Fig. 6a,b). Collectively, these findings demonstrate that the modulators disrupt β-arrestin–GPCR coupling to prevent receptor desensitization.

Agonist-induced β-arrestin recruitment facilitates GPCR internalization^7,10,32^. Given that all three modulators impaired β-arrestin engagement and receptor desensitization, we next examined their effects on receptor endocytosis. Treatment with β-arrestin modulators markedly reduced Iso-induced internalization of β_2_V_2_R–βARR2, as quantified using the PathHunter β-gal complementation assay in U2OS cells (Fig. 2g), with maximal reductions reaching 93% for Cmpd-5, 88% for Cmpd-46 and 87% for Cmpd-64 at 25 μM (Fig. 2h–j). To independently validate these findings, we used bystander BRET to quantify V_2_R–RlucII trafficking to early endosomes via 2×FYVE-mVenus^33^ (Extended Data Fig. 6c). [Arg8]-Vasopressin (AVP) stimulation induced a concentration-dependent increase in BRET signal, reflecting progressive V_2_R accumulation on early endosomes, and all three modulators attenuated this response (Extended Data Fig. 6d,e). Together, these findings reveal that the modulators disrupt β-arrestin–GPCR coupling, impairing agonist-driven receptor internalization.

## Downstream signalling and physiological functions

**Fig. 3:**
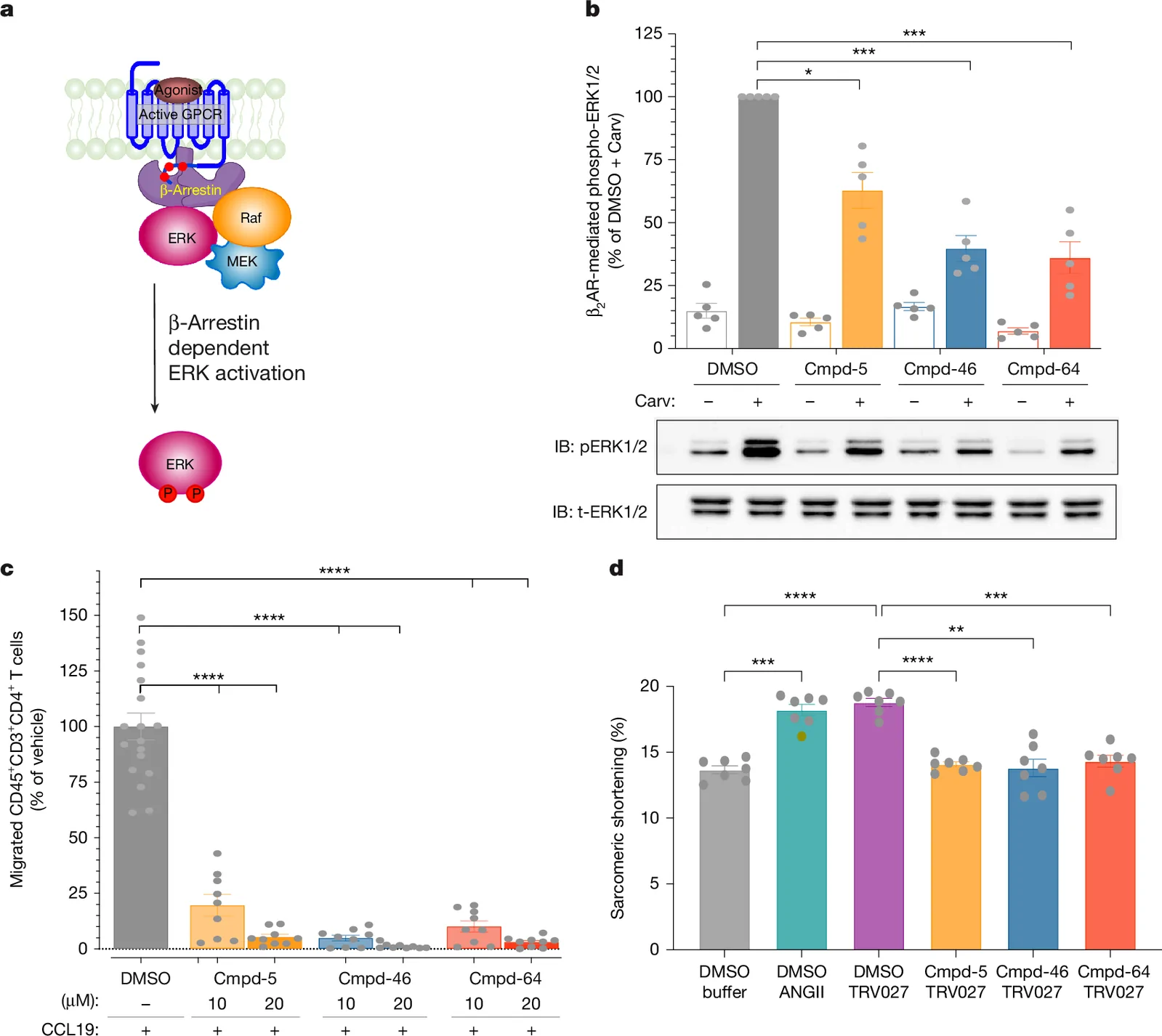
β-Arrestin modulators inhibit ERK signalling, impair T cell migration and blunt β-arrestin-mediated cardiomyocyte contractility. **a**,**b**, Effects of modulators on β-arrestin-dependent ERK signalling downstream of GPCRs. **a**, Schematic model of β-arrestin-dependent ERK activation, in which β-arrestin scaffolds phosphorylated GPCRs to RAF–MEK–ERK signalling components (for blot source data, see Supplementary Fig. 1). **b**, Cmpd-5, Cmpd-46 and Cmpd-64 (30 μM) attenuate ERK1/2 phosphorylation in β_2_AR-expressing cells stimulated with carvedilol (Carv) (10 μM, 5 min), a β-arrestin-biased agonist. Top, quantification of phosphorylated ERK1/2 (pERK1/2) normalized to carvedilol alone. Bottom, representative immunoblots (IBs). *n* = 5 independent experiments. t-ERK1/2, total ERK1/2. **c**, β-Arrestin modulators impair chemokine-driven T cell migration. Wild-type mouse lymphocytes were pretreated with Cmpd-5, Cmpd-46 or Cmpd-64 (10 μM or 20 μM, 30 min), followed by chemotaxis towards CCL19 (100 nM, 2 h), a CCR7 agonist. Migration was quantified relative to the vehicle for CD4^+^ helper T cells. **d**, β-Arrestin modulators blunt β-arrestin-mediated cardiomyocyte contractility. Adult ventricular cardiomyocytes were paced at 1 Hz under field stimulation, pretreated with Cmpd-5, Cmpd-46 or Cmpd-64 (25 μM, 20 min), and then stimulated with buffer, ANGII or the β-arrestin-biased AT_1_R agonist TRV027 (10 μM). TRV027 enhanced sarcomere shortening, consistent with β-arrestin-mediated inotropic signalling, which was attenuated by β-arrestin inhibitors. Summary data from seven mice. Data are mean ± s.e.m. One-way ANOVA (**b**), two-way ANOVA with Dunnett’s post hoc test (**c**) and one-way ANOVA with Sidak’s post hoc test (**d**).

**Extended Data Fig. 7:**
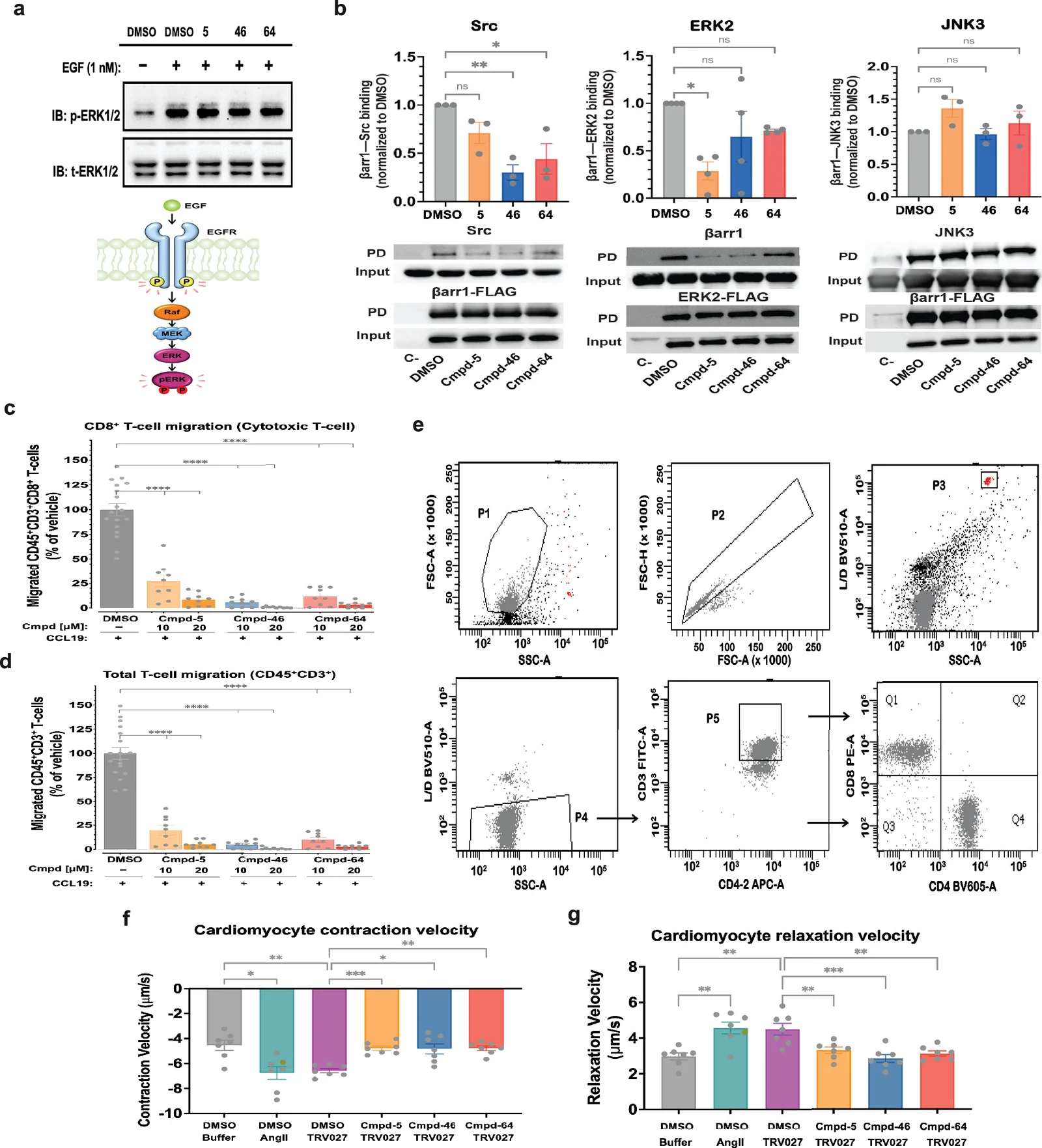
β-arrestin modulators alter effector interactions and impair T cell migration and reduce cardiomyocyte relaxation. **a**, βarr modulators do not affect EGFR-mediated ERK activation. HEK293 cells were pretreated with compounds (30 μM) and stimulated with EGF (1 nM). Immunoblots show phosphorylated and total ERK1/2 (for blot source data, see Supplementary Fig. 2). **b**, Modulation of βarr1 binding to intracellular effectors. Cmpd-5, Cmpd-46, and Cmpd-64 were tested for their effects on βarr1 interactions with Src, ERK2, and JNK3 using FLAG pull-down assays. Densitometric quantification (top) shows reduced βarr1 binding to Src and ERK2, with minimal change for JNK3 (n = 3 (Src), n = 4 (ERK2), n = 3 (JNK3); mean ± s.e.m.). Representative blots shown (bottom) (for blot source data, see Supplementary Fig. 3). **c, d**, Modulators impair chemokine-induced T cell migration. Wild-type mouse lymphocytes were pretreated with Cmpd-5, -46, or -64 (10 or 20 μM, 30 min), followed by chemotaxis toward CCL19 (100 nM, 2 h), a CCR7 agonist. Migration was quantified relative to the vehicle for (c) CD8^+^ cytotoxic T cells and (d) total CD45^+^CD3^+^ T cells expressed as % of vehicle control. CCR7 is a chemokine receptor widely expressed across T cell populations and essential for T cell migration. Average reductions in chemotaxis were approximately 95%, 99%, and 97% for helper, CD8^+^ cytotoxic, and total CD45^+^CD3^+^ T cells at 20 μM, respectively. **e**, Gating strategy for flow cytometric analysis of T cell migration. Sequential gating was performed for total CD3^+^ T cells and CD4^+^/CD8^+^ subsets: P1 (lymphocyte scatter), P2 (singlets), P3–P4 (live cell selection), P5 (CD3^+^), and Q1–Q4 (CD4 vs. CD8 quadrant gating). **f**, **g**, βarr modulators reduce TRV027-induced cardiomyocyte contraction and relaxation velocity. Adult ventricular cardiomyocytes were pretreated with DMSO or modulators (25 μM, 20 min) before stimulation with buffer, AngII (10 μM), or TRV027 (10 μM). TRV027 increased f, contraction velocity (μm/s); g, relaxation velocity (μm/s), which was attenuated by all modulators. n = number of hearts with each data point representing 7–12 cells per heart for each condition; mean ± s.e.m.; One-way ANOVA (b), two-way ANOVA with Dunnett’s post hoc test (c, d), and one-way ANOVA with Sidak’s post hoc test (f, g). ns, not significant; *P ≤ 0.05;**P < 0.01; ***P < 0.001; ****P < 0.0001.

GPCR-stimulated ERK1 and ERK2 (ERK1/2) activation can occur through a β-arrestin-dependent mechanism^3,34^. To probe the effect of the modulators on this pathway, we examined β-arrestin-mediated ERK activation in response to carvedilol, a β-arrestin-biased agonist that blocks Gα_s_ activation through β_1_AR and β_2_AR while selectively inducing β-arrestin-dependent ERK phosphorylation^24,34,35^ (Fig. 3a). Notably, Cmpd-5, Cmpd-46 and Cmpd-64 significantly attenuated βARR1/2-dependent ERK activation downstream of β_2_AR (Fig. 3b). By contrast, none of the modulators affected EGF-induced ERK activation (Extended Data Fig. 7a), indicating that they do not broadly suppress MAPK signalling. Notably, pulldown assays revealed that all modulators markedly reduced βARR1 binding to SRC and ERK2, while leaving JNK3 interactions unaffected (Extended Data Fig. 7b). Together, these findings indicate that the modulators selectively interfere with β-arrestin-dependent signalling downstream of GPCR activation.

β-Arrestins scaffold multiple proteins that regulate cell polarity and direct cellular migration downstream of GPCRs, including chemokine receptors^3,4,36–38^. To determine whether the modulators could disrupt this β-arrestin-dependent migratory function, we assessed CCL19-stimulated T cell migration towards the CCR7 agonist in wild-type mouse T cells^38^ (Fig. 3c and Extended Data Fig. 7c–e). Notably, all three modulators markedly disrupted T cell migration across total, helper (CD4^+^), and cytotoxic (CD8^+^) T cell populations (Fig. 3c and Extended Data Fig. 7c,d), with near-complete inhibition at 20 μM.

Beyond chemotaxis, we examined whether the modulators affect cardiomyocyte contractility driven by β-arrestin signalling downstream of AT_1_R activation. β-Arrestin signalling has been shown to enhance cardiomyocyte contractility and promote cell survival^39–41^, highlighting its role in cardiac physiology. In isolated adult mouse ventricular cardiomyocytes, the β-arrestin-biased ligand TRV027 significantly enhanced sarcomere shortening, contraction velocity and relaxation velocity compared with control (Fig. 3d and Extended Data Fig. 7f,g), consistent with its β-arrestin-mediated inotropic activity. Pretreatment with Cmpd-5, Cmpd-46 or Cmpd-64 (25 μM, 20 min) significantly inhibited these effects, reducing contractile parameters to near-baseline levels. Collectively, these findings establish the modulators as broadly applicable chemical probes of β-arrestin physiology that are capable of disrupting β-arrestin-dependent signalling across physiological contexts.

## Structural and molecular mechanism of inhibition

**Fig. 4:**
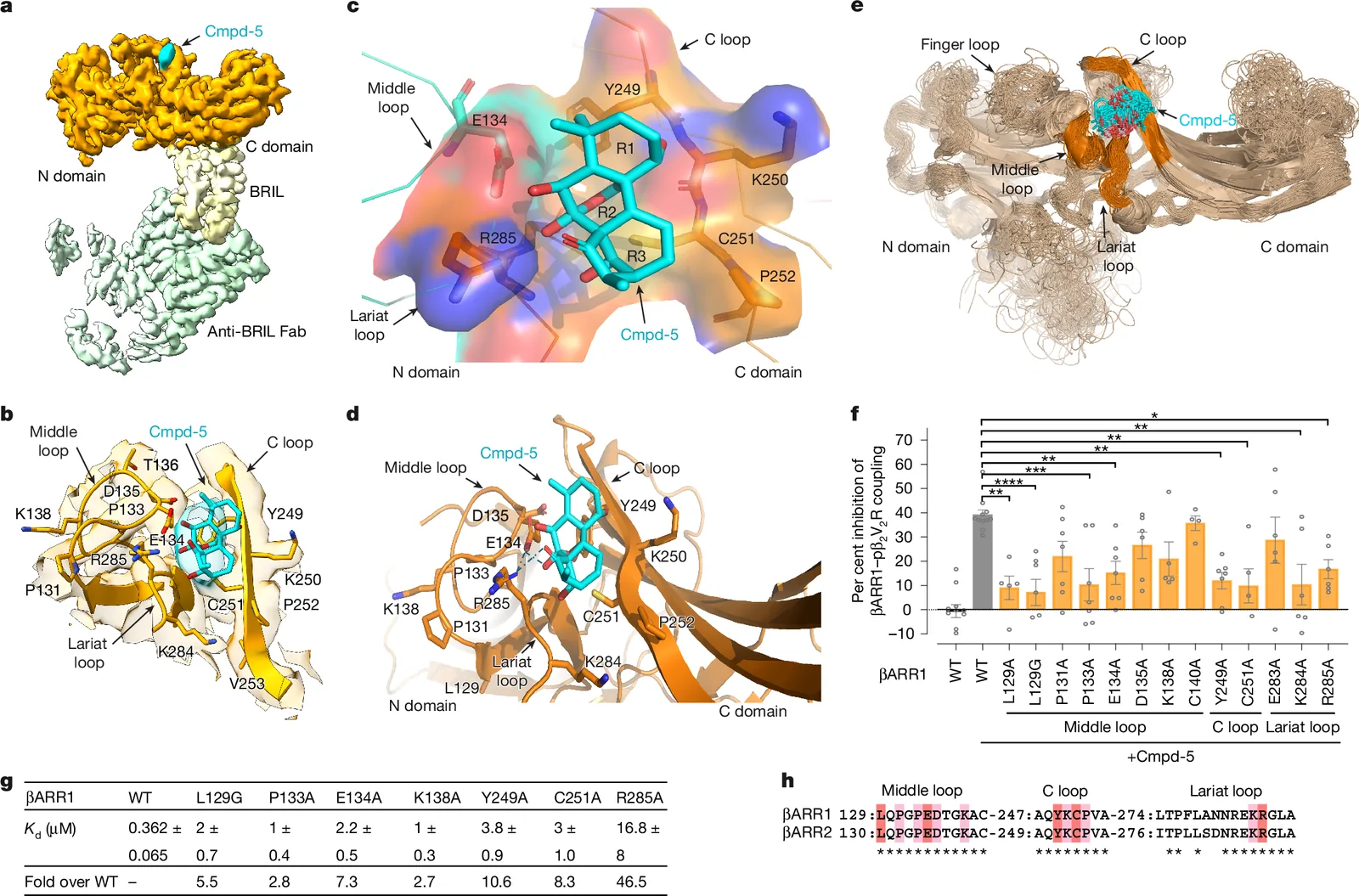
Cmpd-5 binds the MCL site in βARR1 and engages a structurally dynamic interface. **a**, Cryo-EM map of βARR1 bound to Cmpd-5 showing the N domain, C domain, BRIL and anti-BRIL Fab (BAG2). The map was post-processed with DeepEMhancer and contour level is 0.07. **b**, Close-up view of the cryo-EM structure of the MCL site, showing Cmpd-5 positioned among the middle loop, C-loop and lariat loop. Key residues are shown as sticks. Map contour level 0.07. **c**, Electrostatic surface representation of the binding pocket. Cmpd-5 is shown with three moieties: dimethylcyclohexanol (R1), oxabicyclo[2.2.2]octane-diol (R2) and 8-hydroxy-7-methylenebicyclo[3.2.1]octan-6-one (R3). **d**, Cmpd-5 interactions with βARR1, showing hydrogen bonds (dashed lines) inferred from structural data, molecular dynamics simulations, and mutagenesis. **e**, Snapshots of molecular dynamics simulations illustrating Cmpd-5 binding within its pocket while accommodating conformational flexibility both in its structure and in the surrounding middle loop, lariat loop, C-loop and finger loop. **f**, Functional effect of MCL site residue mutations on βARR1 modulation, assessed using [^3^H]-Fen binding assays. Cmpd-5 inhibited βARR1-mediated stabilization of the high-affinity β_2_V_2_R state by around 40%. Data are mean ± s.e.m. from at least 4 experiments. One-way ANOVA with Dunnett’s test. WT, wild type. **g**, ITC analysis of Cmpd-5 binding to wild-type and mutant βARR1. *K*_d_ values (mean ± s.e.m.) were derived from fits to a one-site binding model. **h**, Sequence alignment of rat βARR1 and βARR2 MCL site residues. Key residues involved in Cmpd-5 binding are highlighted in red (direct) and pink (indirect).

**Extended Data Fig. 8:**
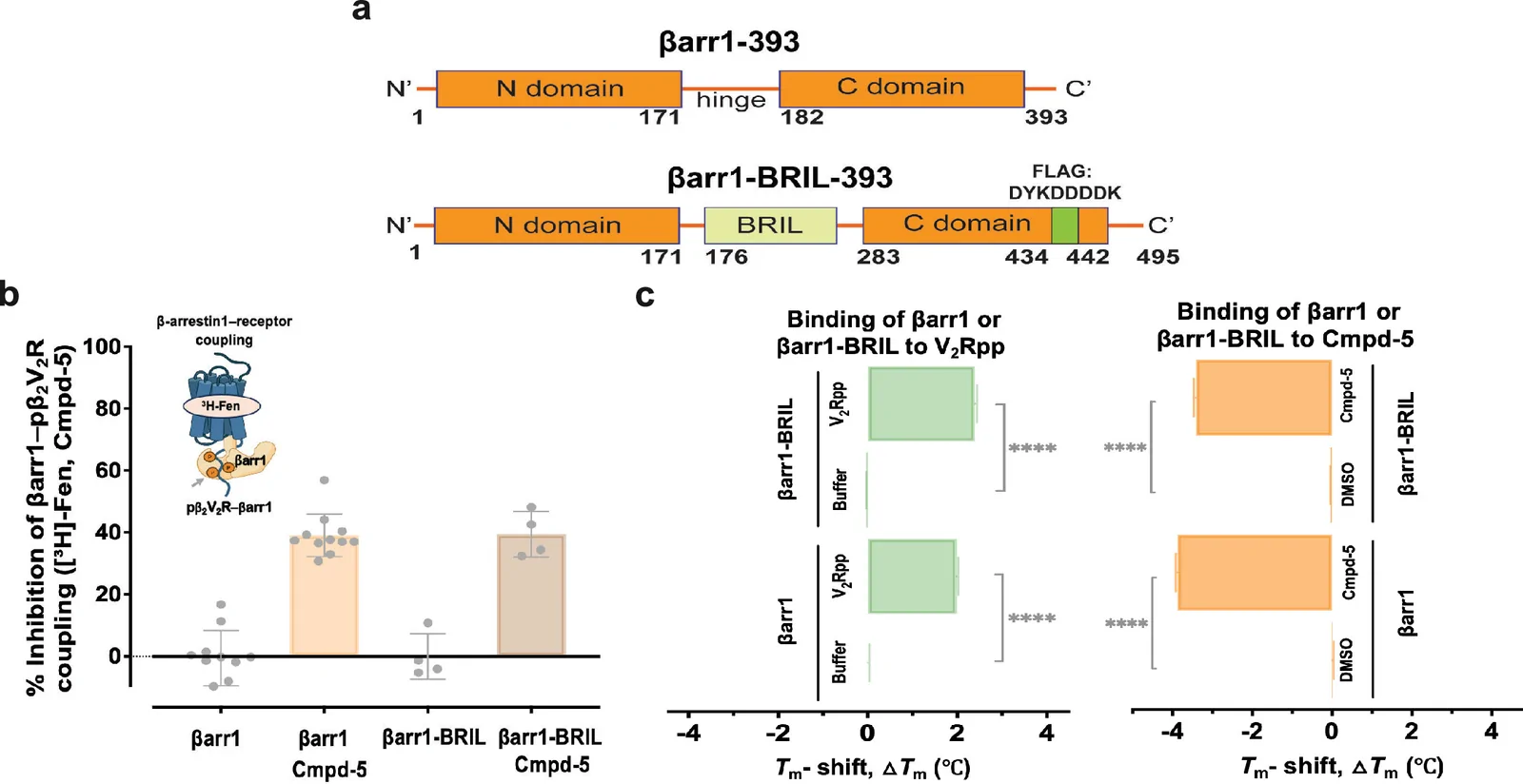
BRIL fusion to βarr1 preserves functional integrity for cryo-EM studies with Cmpd-5. **a**, Schematic of the βarr1–BRIL construct design. The BRIL domain was inserted into the hinge region of βarr1 as a fiducial marker for cryo-EM. Residue numbers shown in the schematic correspond to positions after insertion of the BRIL (apocytochrome b562) fusion protein. **b**, Effect of Cmpd-5 (100 μM) on βarr1 (2 μM) and βarr1–BRIL (2 μM) promoted high-affinity agonist ([^3^H]-Fenoterol, 6 nM) binding to β_2_V_2_R in vitro. The data show that BRIL fusion does not impair the functional interaction between βarr1 and the receptor or Cmpd-5-mediated inhibition. Inhibition is expressed as a percentage relative to control (n = 4). Schematic created in BioRender; Kahsai, A. https://BioRender.com/pw680be (2026). **c**, Binding of βarr1 and βarr1–BRIL to V2R phosphorylated peptide (V2Rpp) (left) and Cmpd-5 (right), assessed by thermal stability shifts (ΔTm) using DSF. The results confirm that BRIL fusion does not alter βarr1 binding to either the V_2_Rpp or Cmpd-5 (n = 3).

**Extended Data Fig. 9:**
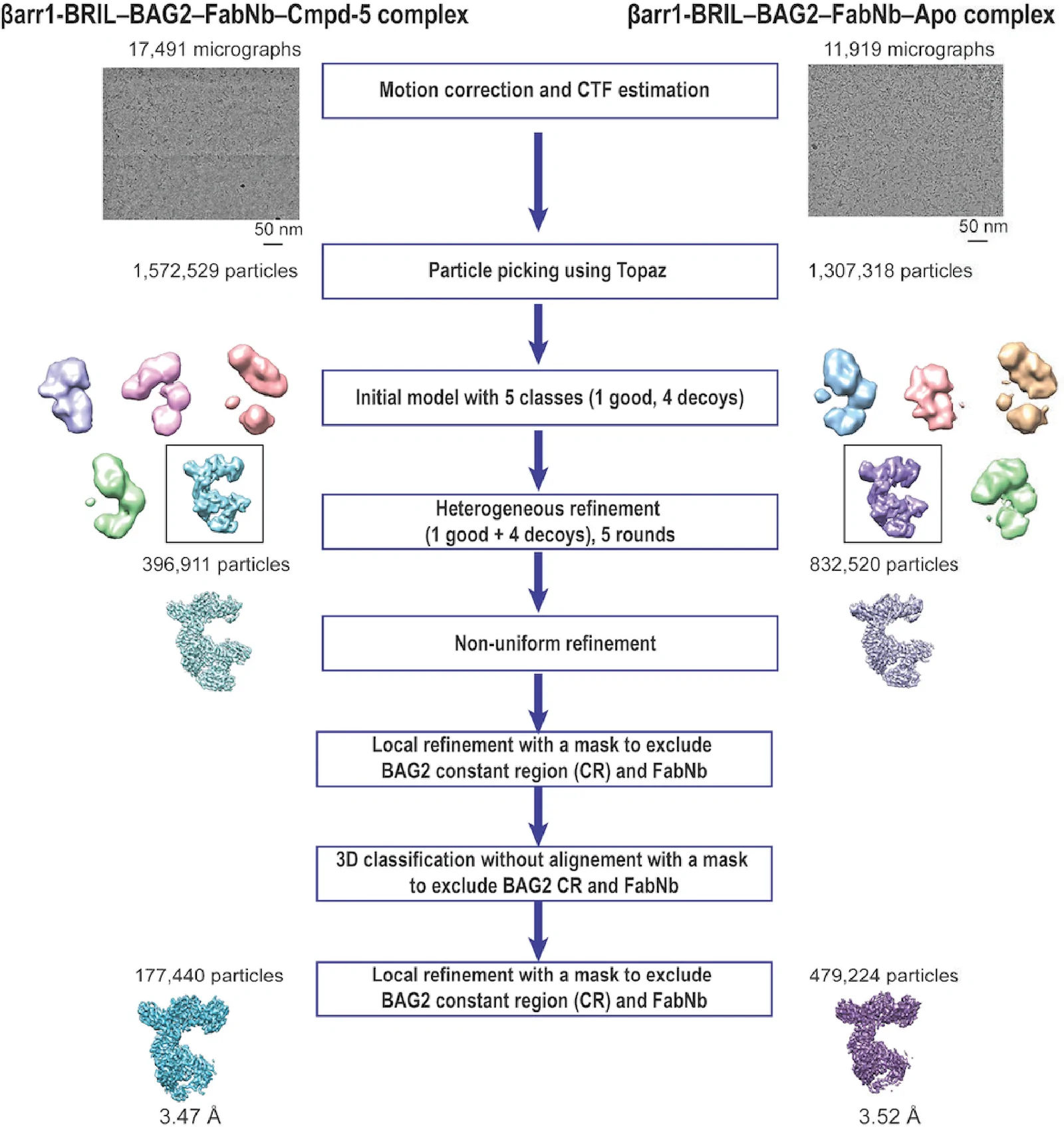
Flow chart of cryo-EM data processing for βarr1–BRIL–BAG2–aFabNb–Cmpd-5 and βarr1–BRIL–BAG2–aFabNb–Apo complexes. Representative motion-corrected micrographs and corresponding data processing flowcharts for the Cmpd-5-bound (left, 17,491 micrographs, 1,572,529 particles initially picked) and apo (right, 11,919 micrographs, 1,307,318 particles picked) complexes. Particles were picked using Topaz and subjected to initial model generation with 5 classes (1 good, 4 decoys), followed by multiple rounds of heterogeneous refinement. Particle stacks were further processed by non-uniform refinement, local refinement with masking to exclude the BAG2 constant region and FabNb, and 3D classification without alignment, yielding final reconstructions of 177,440 and 479,224 particles at 3.47 Å and 3.52 Å for the Cmpd-5-bound and apo complexes, respectively. All data processing was performed in cryoSPARC.

**Extended Data Fig. 10:**
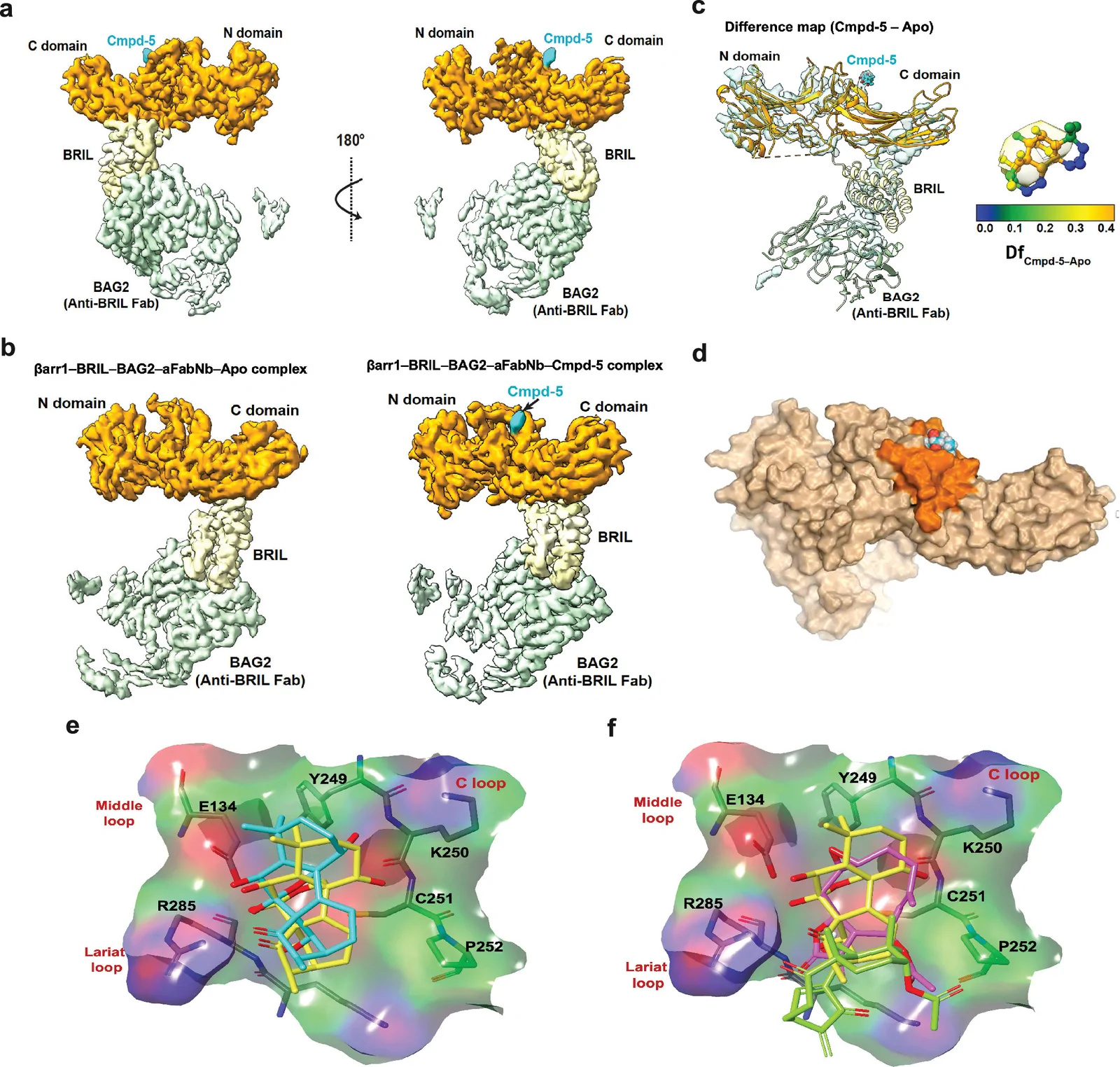
Cryo-EM density maps and structural insights into the βarr1–BRIL–BAG2–aFabNb–Cmpd-5 complex. **a**, Two views of the cryo-EM density map of the βarr1–BRIL–BAG2–aFabNb–Cmpd-5 complex, colored by subunit (βarr1, orange; BRIL, pale yellow; BAG2 (anti-BRIL Fab), light green; Cmpd-5, cyan). Post-processing was performed using DeepEMhancer with a map contour level of 0.07. **b**, Cryo-EM density maps comparing the βarr1–BRIL–BAG2–aFabNb complex in the apo (3.52 Å, left) and Cmpd-5-bound (3.47 Å, right) states. Subunits are colored as in panel a. Maps were post-processed with DeepEMhancer at a contour level of 0.07. **c**, Left, local density differences between the Cmpd-5-bound and apo states (difference map: Cmpd-5–Apo) calculated as described. The atomic model of the Cmpd-5-bound state shows βarr1 (orange), Cmpd-5 (cyan), BRIL (pale yellow), and BAG2 (light green). Right, density map of Cmpd-5 modeled with a ball-and-stick representation, colored by fractional density difference (Df, Cmpd-5–Apo) using a local scaling-based density difference approach. **d**, Molecular dynamics (MD) simulation snapshot showing Cmpd-5 binding within the MCL site of βarr1. The MCL site is colored orange, Cmpd-5 is cyan, and βarr1 is shown in wheat. This highlights the stable interaction of Cmpd-5 within the cleft. **e**, **f**, Docking of βarr modulators at the Cmpd-5 binding site. **e**, Cmpd-5 occupies the MCL site of βarr1, engaging the middle, lariat, and C loops. The cyan pose represents the cryo-EM–resolved binding mode; the yellow pose shows the best-scoring docking result from Schrödinger Glide (Suite 2025.2). Both poses align closely, with consistent orientations of key functional groups and amino acid contacts (docking score: –4.13). **f**, Docking of structurally distinct βarr modulators Cmpd-46 (green) and Cmpd-64 (magenta) into the same site resulted in poses lacking shape and contact complementarity, with lower predicted docking scores (–3.15 and –3.34, respectively).

To elucidate the molecular basis of β-arrestin inhibition, we determined the cryo-electron microscopy (cryo-EM) structure of the βARR1–Cmpd-5 complex. Cmpd-5 was prioritized for its ability to bind both β-arrestin isoforms with comparable affinity (Fig. 1e and Extended Data Fig. 3) and its aqueous stability, which supported reliable cryo-EM reconstruction. To improve particle alignment, we inserted the apocytochrome b562RIL (BRIL) domain at the flexible hinge region of βARR1 and used an anti-BRIL Fab (BAG2)^42^, along with an anti-Fab nanobody (aFabNb)^43^ as fiducial markers (Fig. 4a and Extended Data Fig. 8a). BRIL insertion preserved βARR1 function, with the modified construct retaining Cmpd-5 and V_2_R phosphorylated peptide (V_2_Rpp) binding and high-affinity agonist stabilization of pβ_2_V_2_R (Extended Data Figs. 8b,c and 9 and Extended Data Table 1). The resulting cryo-EM reconstruction resolved the βARR1–Cmpd-5 complex at 3.47 Å (Fig. 4a and Extended Data Fig. 10a). Notably, density corresponding to Cmpd-5 localized to a distinct pocket within the central crest of βARR1, formed by the middle loop (residues 129–140), the C-loop (residues 241–249, including β-strand XVII) and the lariat loop (residues 274–300), collectively referred to here as the MCL site (Fig. 4a–d). Unlike the canonical N domain groove that engages phosphorylated C-terminal tails of GPCRs^44^, the MCL site represents a spatially and structurally distinct pocket.

To validate the binding of Cmpd-5 within the MCL site, we determined the cryo-EM structure of apo βARR1–BRIL at 3.52 Å resolution (Extended Data Figs. 9 and  10b). No density was observed at the MCL site in the apo map, in marked contrast to the Cmpd-5-bound complex, providing unambiguous evidence for compound occupancy (Fig. 4a and Extended Data Fig. 10b, right). A difference density map^45^ generated by subtracting the apo density from the Cmpd-5-bound structure corroborated this, revealing additional density precisely at the MCL site (Extended Data Fig. 10c). Modelling the compound in a specific conformation remained challenging owing to its inherent conformational flexibility and peripheral positioning within the pocket. This flexibility, also evident in molecular dynamics simulations described below, suggests that the cryo-EM density may not fully capture all conformational states of Cmpd-5, a natural product with multiple chiral centres^46^ (Fig. 4e, Extended Data Fig. 10d and Extended Data Table 2). The structure revealed Cmpd-5 engaging both hydrophilic and hydrophobic residues at the βARR1 interface (Fig. 4b–d). Its cyclic hydrocarbon rings packed against hydrophobic patches in the C-loop near Y249 and C251, and E134 (middle loop), K250 (C-loop) and possibly R285 (lariat loop) contributed polar contacts with oxygen atoms of Cmpd-5. Although R285 side-chain density was unresolved, its position suggests accommodation of Cmpd-5 hydroxyl groups, consistent with binding dynamics that are likely to extend beyond the static cryo-EM snapshot (Fig. 4d,e and Extended Data Fig. 10d).

Molecular dynamics simulations confirmed stable Cmpd-5 residency within the MCL site, with the surrounding loops exhibiting dynamic flexibility while the compound continuously sampled polar contacts with R285 and hydrophobic interactions with Y249, C251 and L129 (Fig. 4e, Extended Data Fig. 10d and Supplementary Videos 1 and 2). Absolute binding free energy perturbation calculations estimated the binding free energy at –4.1 ± 0.1 kcal mol^−1^, consistent with the experimental binding affinity. Docking of Cmpd-5 at the MCL site further yielded favourable poses with consistent functional group orientations (Extended Data Fig. 10e). By contrast, a weak density near C269 showed poor binding complementarity, consistent with compound trapping near the BRIL insertion, and was not modelled. Cmpd-46 and Cmpd-64 showed disfavoured binding at the MCL site, with poor complementarity to the pocket (Extended Data Fig. 10f).

**Extended Data Fig. 11:**
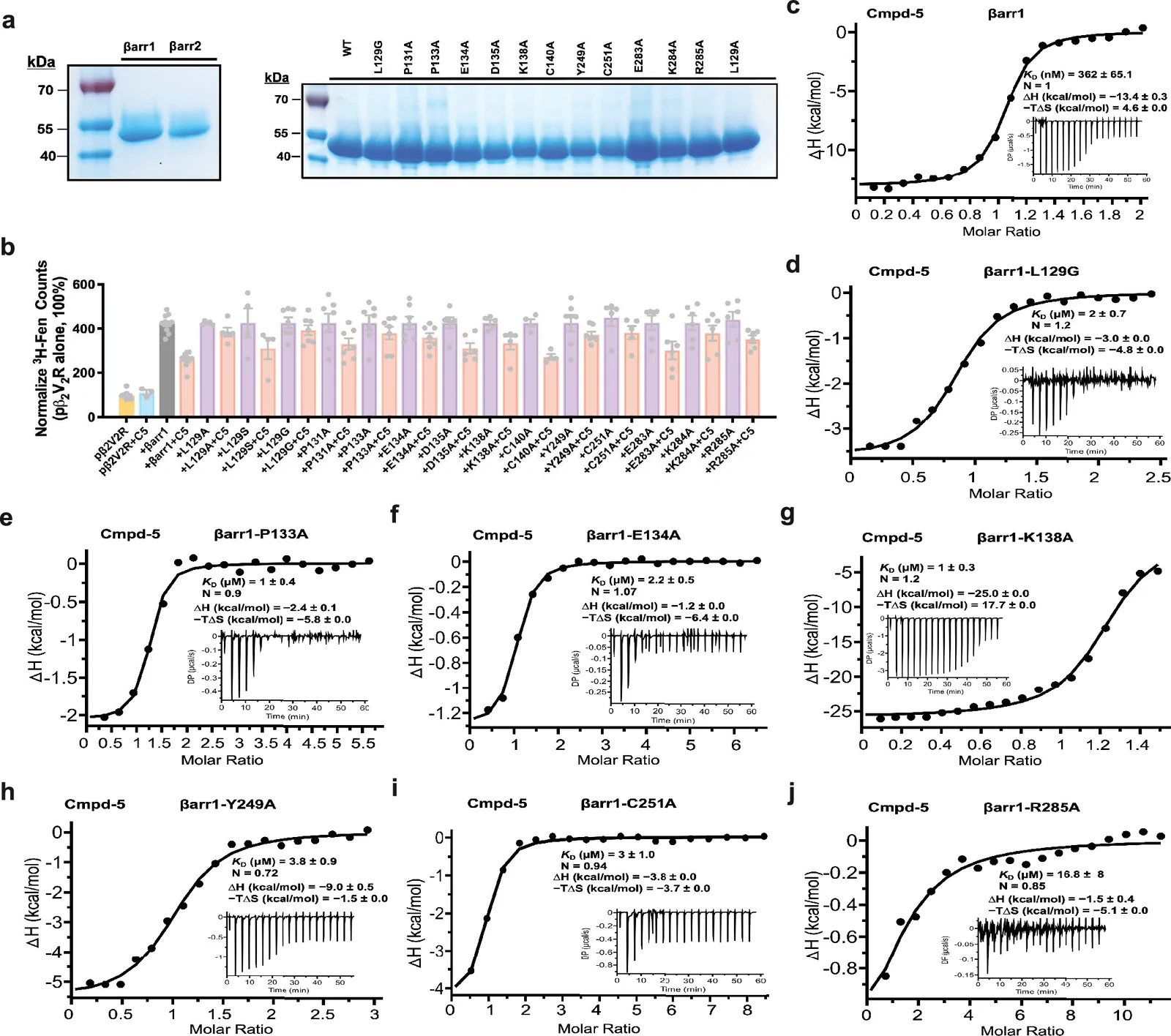
Mutagenesis, biochemical, functional, and biophysical validation of Cmpd-5 binding to βarr1. **a**, SDS–PAGE gels of purified recombinant βarr1–393, βarr2–394, and βarr1 point mutants targeting the Cmpd-5 binding pocket (for gel source data, see Supplementary Fig. 4 and for oligonucleotide primers, see Supplementary Table 1). Molecular weights were estimated using PageRuler™ Prestained Protein Ladder. **b**, Functional assay assessing Cmpd-5 inhibition of βarr1- and mutant-promoted high-affinity agonist binding to β_2_V_2_R using [^3^H]-Fenoterol (6 nM) in Sf9 cell membranes. Data are normalized to pβ_2_V_2_R alone (100%) and represent mean ± s.e.m. from at least four independent experiments. **c**–**j**, ITC analysis of Cmpd-5 binding to βarr1 and its mutants at 25 °C. Representative thermograms (insets) and isotherms for (**c)** WT, **(d**) L129G, (**e**) P133A, (**f**) E134A, (**g**) K138A, (**h**) Y249A, (**i**) C251A, and (**j**) R285A. Data were fitted using a one-site binding model, reporting *K*_d_, stoichiometry (n), ΔH, and –TΔS with s.e.m.

To explore the structural determinants of Cmpd-5 binding, we performed targeted mutagenesis guided by the cryo-EM structure, introducing point mutations at key residues lining the MCL pocket (Extended Data Fig. 11a). The effect of each mutation was assessed by measuring Cmpd-5-mediated inhibition of βARR1 coupling to pβ_2_V_2_R using [^3^H]-Fen, alongside ITC-based binding affinities for wild-type and mutant βARR1 variants (Fig. 4f,g and Extended Data Fig. 11b–j). Mutations at L129G, P133A, E134A, Y249A, C251A, K284A and R285A reduced Cmpd-5 inhibition of βARR1 coupling by 2.4- to 5.5-fold relative to wild type (Fig. 4f and Extended Data Fig. 11b), whereas P131A, D135A, K138A, C140A and E283A had negligible effects. A subset of mutants was further characterized by ITC to probe individual side-chain contributions to Cmpd-5 recognition (Fig. 4g and Extended Data Fig. 11c–j). Mutations at L129, P133, E134, K138, Y249, C251 and R285 reduced binding affinity by 5.5- to 46.5-fold relative to wild-type βARR1. Notably, E134A and R285A impaired both binding and functional activity, underscoring their role in stabilizing polar interactions. R285A caused a 46.5-fold loss in affinity, with the enthalpic contribution decreasing from −13.4 kcal mol^−1^ in wild type to −1.5 kcal mol^−1^ (Extended Data Fig. 11j), and E134A showed a similarly diminished enthalpy of −1.2 kcal mol^−1^ (Extended Data Fig. 11f), consistent with disruption of key polar contacts observed in the cryo-EM structure. Y249A and C251A weakened affinity by 10.6- and 8.3-fold, respectively, probably through loss of hydrophobic and secondary polar contacts. Binding was clearly retained upon C251A substitution, consistent with a non-covalent mode of engagement^46^ (Extended Data Fig. 11h,i). L129 and P133, though not in direct contact with Cmpd-5, influenced binding through a structural role in stabilizing the pocket conformation (Extended Data Fig. 11d,e). Together, the mutagenesis data validate the critical contacts mediating Cmpd-5 recognition and support the allosteric binding model derived from cryo-EM and molecular dynamics simulations. Sequence conservation of MCL site residues between βARR1 and βARR2 (Fig. 4h) suggests a shared mode of engagement across isoforms (Fig. 1f–i and Extended Data Figs. 2a,b, 3a and 5).

## A distinct inhibitor-bound conformational state

**Fig. 5:**
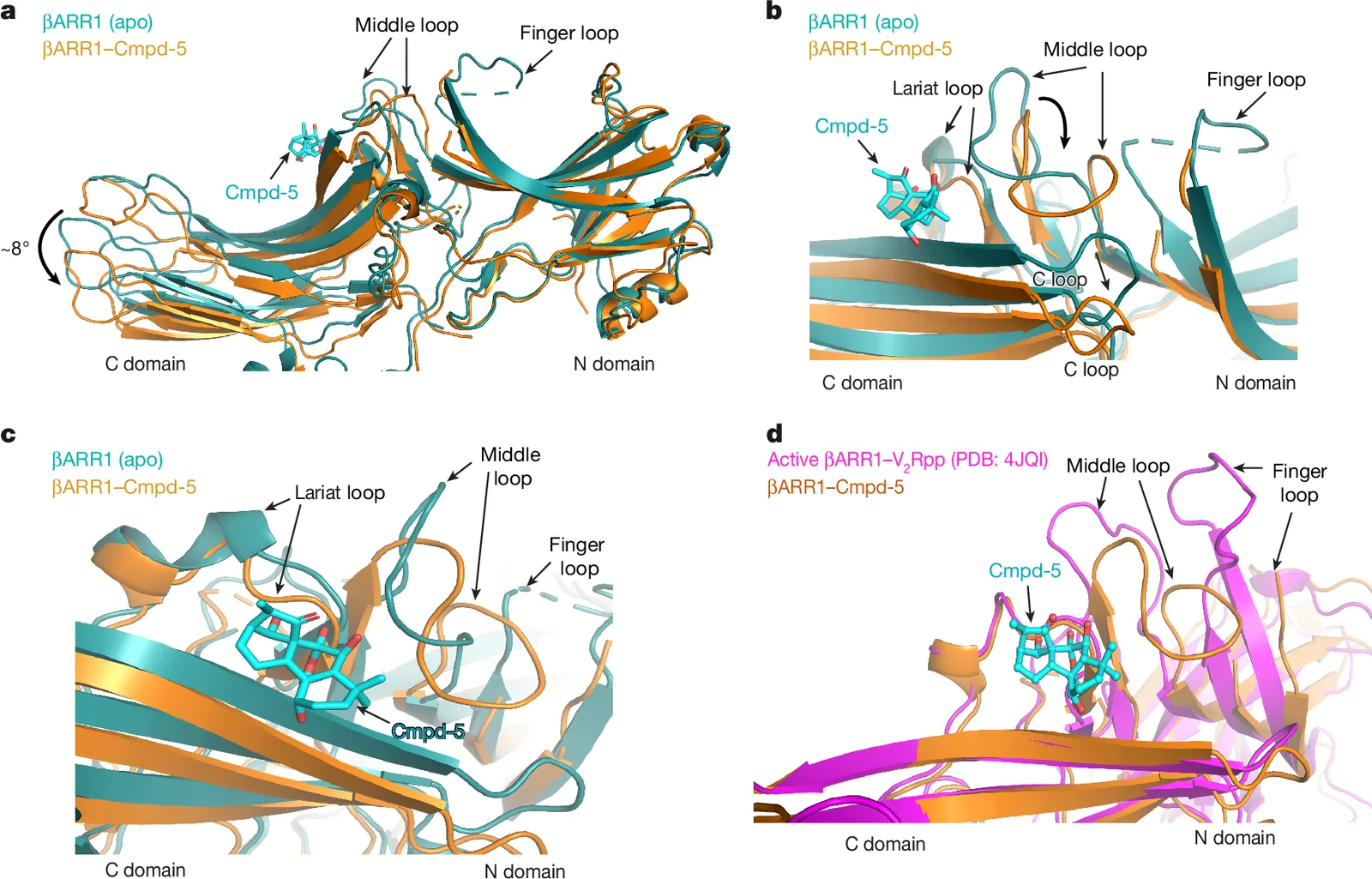
Structural insights into Cmpd-5–mediated inhibition of βARR1. **a**, Structural comparison of Cmpd-5-bound βARR1 with apo βARR1, superimposed by their N domains. Cmpd-5 binding induces an 8° interdomain twist, resulting in a poorly resolved finger loop. This twist stabilizes βARR1 in a conformation that is incompatible with receptor binding or activation. The Cmpd-5-bound and apo structures diverge with a Cα r.m.s.d. of 1.34 Å and *Q*-score of 0.652. **b**,**c**, Conformational rearrangements in the middle loop and C-loop (**b**) and lariat loop (**c**) within the MCL site. Cmpd-5 shifts the middle loop towards the N domain and induces a helix-to-loop transition (residues 282–285) in the lariat loop. Black arrows indicate structural changes. **d**, Structural overlay of the Cmpd-5-bound βARR1 cryo-EM structure with the V_2_Rpp-bound βARR1 complex (Protein Data Bank (PDB): 4JQI), highlighting conformational differences induced by Cmpd-5 binding at the MCL site relative to the receptor-bound state. Cmpd-5-bound βARR1 diverges from the active structure (r.m.s.d. = 1.64 Å, *Q*-score = 0.575), supporting a distinct, inactive-like state incompatible with receptor engagement.

**Extended Data Fig. 12:**
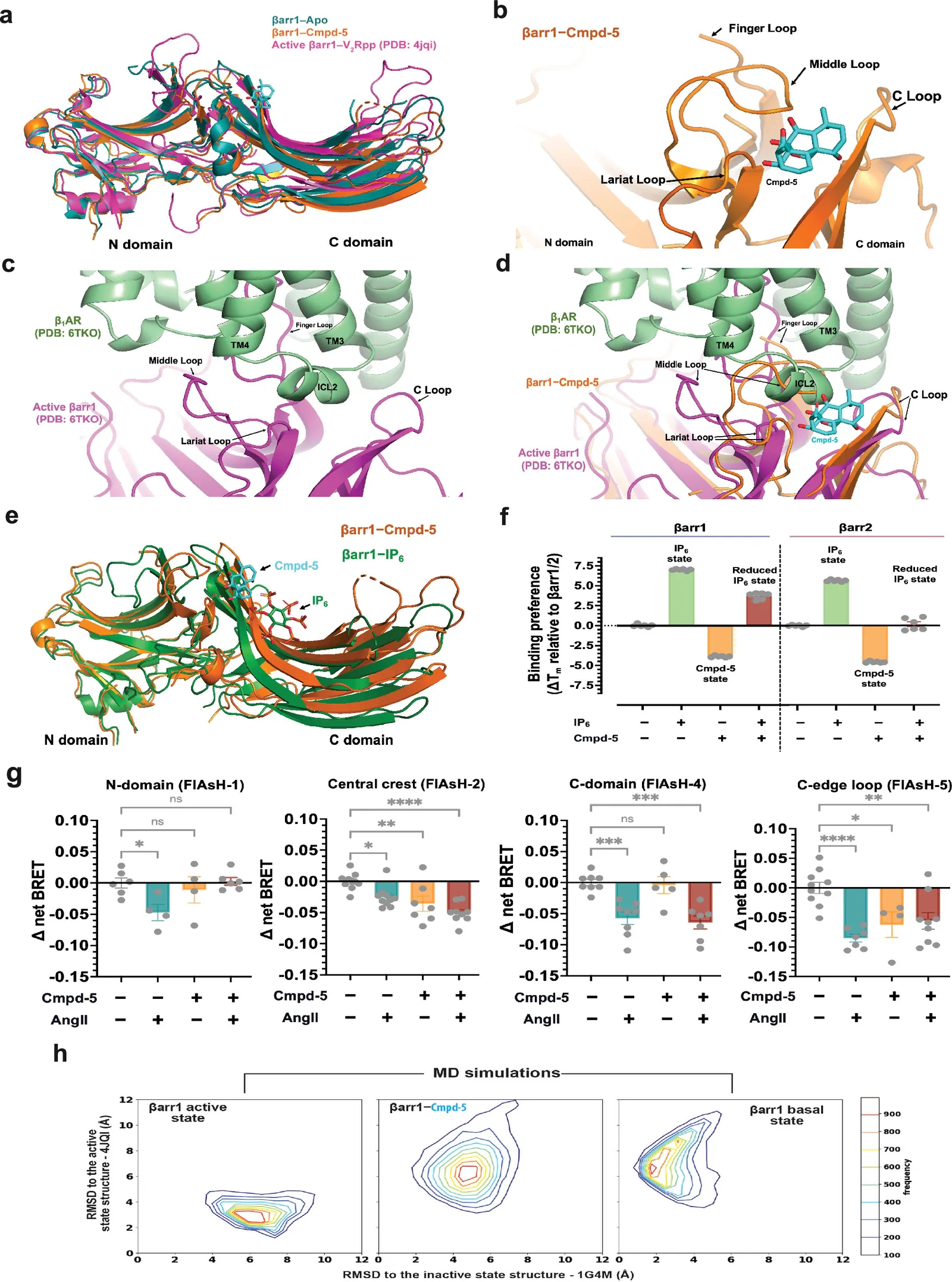
Structural basis and mechanism of Cmpd-5 binding and inhibition of βarr1. **a**, Structural superposition of Cmpd-5–bound βarr1 (orange), apo–βarr1 (deep teal), and the active-state βarr1–V_2_Rpp complex (magenta; PDB: 4JQI), superimpose by their N domains, highlights the unique conformation of the Cmpd-5–bound state. The Cmpd-5–bound structure deviates moderately from apo–βarr1 (root-mean-square deviation, RMSD = 1.338 Å, Q-score = 0.652), but more substantially from the V_2_Rpp-bound active conformation (RMSD = 1.636 Å, Q-score = 0.575). By comparison, apo and active βarr1 conformations differ most strongly (RMSD = 2.102 Å, Q-score = 0.487). These comparisons support that Cmpd-5 stabilizes a distinct, inactive-like βarr1 state incompatible with receptor engagement. RMSD and Q-score values were calculated using PDBeFold. **b**, Close-up view of the Cmpd-5 binding site within the central crest of βarr1 (orange), formed by the middle loop, C-loop, and lariat loop (MCL site). Cmpd-5 is shown in cyan. **c**, Close-up view of the pβ_1_V_2_R–βarr1 complex (PDB: 6TKO), with pβ_1_V_2_R in pale green and active βarr1 in magenta. **d**, Structural overlay of the cryo-EM structure of βarr1 bound to Cmpd-5 (cyan) with the β_2_V_2_R–βarr1 complex (PDB: 6TKO), highlighting conformational changes induced by Cmpd-5 binding. **e**, Structural superposition of βarr1–Cmpd-5 (orange) and IP6–bound βarr1 (green, PDB: 1ZSH), superimposed by their N domains, shows that Cmpd-5 and IP6 occupy distinct sites. **f**, DSF (ΔT_m_, right) in the presence of IP_6_ and/or Cmpd-5 demonstrate that Cmpd-5 shifts βarr1/2 away from the IP_6_-stabilized active state, favoring a distinct, inactive-like state when both ligands are present. **g**, Intramolecular FlAsH–BRET sensors reveal that Cmpd-5 stabilizes a distinct βarr2 conformation. ΔnetBRET was measured using four sensors (F1, F2, F4, F5) inserted at defined βarr2 locations. Cmpd-5 alone induces BRET signatures partially similar to basal (DMSO) and clearly distinct from the active conformation induced by AngII-stimulated AT_1_R. When co-applied with AngII, Cmpd-5 largely attenuates this active signature. These patterns support that Cmpd-5 favors a βarr2 conformation incompatible with receptor engagement. Data are mean ± s.e.m. One-way ANOVA with Dunnett’s test; ns, not significant; *P < 0.05; **P < 0.01; ***P < 0.001; ****P < 0.0001. **h**, Cmpd-5 binding and conformational effects on βarr1 revealed by MD simulations. Two-dimensional (2D) contour plots showing the distribution of active, basal, and Cmpd-5-bound βarr1 states. The RMSD plot compares Cmpd-5-bound βarr1 from MD simulations relative to the basal (x-axis) and active (y-axis) states. The Cmpd-5–bound βarr1 adopts a conformation distinct from both canonical active and inactive states, preventing receptor engagement.

To reveal the conformational basis of Cmpd-5-mediated inhibition of βARR1, we compared the Cmpd-5-bound and apo-state structures (root mean square deviation (r.m.s.d.) = 1.338 Å; Fig. 5a–c). The overall architecture was largely preserved, but Cmpd-5 binding induced marked rearrangements in the central crest, accompanied by a minor interdomain rotation of about 8° (Fig. 5a); difference density maps revealed additional density near the N domain, probably reflecting this conformational rearrangement (Extended Data Fig. 10c). Structural comparison with the V_2_Rpp-bound active state revealed pronounced differences in the central crest (r.m.s.d. = 1.64 Å; Fig. 5d) that were greater than those between the Cmpd-5-bound and apo structures, but smaller than the divergence between apo and active βARR1 (r.m.s.d. = 2.10 Å) (Extended Data Fig. 12a), placing the Cmpd-5-bound state as structurally closer to the basal state but distinct from both. In the canonical active β-arrestin conformation, the MCL site expands to accommodate receptor elements including intracellular loop 2 (ICL2), driven by middle loop displacement and an interdomain twist of approximately 20°. By contrast, Cmpd-5 promotes middle loop bending towards the C-domain, repositioning its tip to cap and constrict the MCL pocket (Fig. 5b), alongside displacement of the C-loop and a helix-to-loop transition in the lariat loop (residues 282–285) (Fig. 5c), collectively producing the modest interdomain twist of about 8°, distinct from the fully active state (Fig. 5a–d and Extended Data Fig. 12b). This rearrangement brought the rear-facing bend of the middle loop closer to the finger loop, probably contributing to its increased flexibility, consistent with the weaker density observed in the Cmpd-5-bound structure. Notably, although the finger loop does not directly contact Cmpd-5, molecular dynamics simulations showed that it frequently engages the N domain groove (Fig. 4e and Supplementary Video 1), potentially adopting conformations that would interfere with phosphorylated receptor tail engagement. Collectively, these coordinated rearrangements are consistent with Cmpd-5 stabilizing a β-arrestin conformation that limits GPCR engagement at both core and tail receptor interfaces (Extended Data Fig. 12b–d).

Converging evidence from structural overlays, DSF analysis and live-cell biosensors further supports a unified conformational model. Cmpd-5 and inositol hexaphosphate (IP_6_) occupy non-overlapping pockets, each stabilizing a distinct β-arrestin conformation (Extended Data Fig. 12e,f). Consistent with this, DSF analysis showed that Cmpd-5 shifts the conformational equilibrium away from the IP_6_-promoted active state (Extended Data Fig. 12f) and intramolecular fluorescein arsenical hairpin (FlAsH)-BRET biosensors in live cells revealed conformational signatures resembling the basal state, diverging from the ANGII-stimulated active βARR2 conformation (Extended Data Fig. 12g). Together, these findings indicate that Cmpd-5 drives βARR1 into a conformation that is distinct from both active and basal states—but closer to the basal state—and incompatible with full receptor engagement (Extended Data Fig. 12h).

## Discussion

β-Arrestins have a central role in regulating diverse GPCR signalling pathways, influencing both physiological functions and pathophysiological processes^3,4,12^. Their functions have largely been investigated through genetic approaches, including knockouts, gene silencing and more recently CRISPR–Cas9-based editing^3,4,24,47–49^, as well as indirect pharmacological strategies such as targeting AP-2 with barbadin^50^. Yet genetic approaches lack reversibility and temporal precision and can trigger compensatory responses that obscure β-arrestin function^24^, whereas indirect pharmacological tools lack the selectivity needed to isolate β-arrestin-specific effects. Here we report direct small-molecule inhibitors of β-arrestins, identified through biophysical screening and characterized across biochemical, pharmacological, structural and computational analyses. Cmpd-5, Cmpd-46 and Cmpd-64 disrupt interactions of β-arrestin with active GPCRs, impairing receptor desensitization, internalization and downstream β-arrestin-dependent signalling across diverse physiological contexts.

By selectively uncoupling β-arrestins from receptors, the modulators attenuated receptor desensitization^1,2,8,9^, thereby sustaining G-protein signalling and prolonging second messenger responses downstream of β_2_AR–Gα_s_ and AT_1_R–Gα_q_ coupling. Consistently, disruption of β-arrestin-stabilized high-affinity receptor states and impaired core coupling imply an allosteric mechanism of inhibition. Indeed, such functional uncoupling may carry therapeutic relevance in contexts where β-arrestin-mediated desensitization is itself detrimental. In asthma, for instance, β-arrestin-driven attenuation of β_2_AR signalling is thought to contribute to airway hyperresponsiveness, whereas G-protein signalling promotes bronchodilation^17,51,52^. Similarly, at GLP-1R, a clinically proven metabolic target, inhibiting βARR1 recruitment is proposed to raise the possibility of sustaining Gα_s_-mediated cAMP signalling linked to prolonged insulin secretion^53,54^. Collectively, these findings suggest that selective pharmacologic disengagement of the β-arrestin arm of GPCR signalling may represent a compelling strategy to fine-tune receptor output, with broader implications for pathway-specific biased agonism^3,12^.

Beyond desensitization, β-arrestins coordinate GPCR-associated signalling complexes to mediate G-protein-independent pathways, including ERK1/2 activation, by acting as scaffolds and allosteric regulators across diverse signalling networks^3,6,12,16,24,34^ and scaffolding proteins involved in cell polarity and actin dynamics downstream of GPCRs^3,36^. Consistent with this broad regulatory role, all three modulators inhibited β-arrestin-dependent ERK activation downstream of β_2_AR, an effect that extended to β-arrestin-mediated chemokine-induced T cell migration, consistent with previous reports of reduced CCR4-, CXCR3- and AT_1_R-mediated chemotaxis^3,37,55,56^. Similarly, in primary cardiomyocytes, the modulators further inhibited contractility downstream of AT_1_R activation driven by the β-arrestin pathway, extending the functional reach of β-arrestin inhibition to a cardiac context. Collectively, these findings underscore the broad physiological reach of β-arrestins and establish these modulators as chemical probes to interrogate β-arrestin-regulated signalling, opening prospects for fine-tuning GPCR pathways in pathophysiological contexts^3,4^. Of note, although the compounds bind β-arrestins with respectable affinity, cellular activity required higher concentrations, probably reflecting limitations in permeability or intracellular stability, properties that are amenable to medicinal chemistry optimization. Their favourable drug-like characteristics, including acceptable aqueous solubility, mark them as promising starting points for further development.

Structural analyses integrating cryo-EM, molecular dynamics simulations, mutagenesis and conformational biosensors converge on a unified model in which Cmpd-5 exploits the MCL site, a pocket formed by the middle loop, C-loop and lariat loops of βARR1, that critically overlaps with the ICL2-engaging receptor-binding interface of active β-arrestins^44,57–60^. In contrast to the approximately 20° interdomain twist that opens this pocket during canonical β-arrestin activation, Cmpd-5 stabilizes a distinct conformation marked by middle loop inward bending, C-loop displacement and a lariat loop helix-to-loop transition, producing a modest twist of about 8° that constricts the MCL pocket, effectively limiting the interdomain flexibility required for full β-arrestin activation. This is further compounded by molecular dynamics simulations, which reveal that the finger loop frequently engages the N domain groove, potentially occluding phosphorylated receptor tail engagement. Converging evidence from structural overlays, DSF analysis, molecular dynamics simulations and conformational biosensors in live cells collectively indicates that Cmpd-5 drives β-arrestins into an inactive-like conformation that is incompatible with full receptor engagement at both core and tail interfaces, favouring an allosteric mechanism of inhibition, although partial competitive inhibition cannot be excluded. Conservation of the MCL site across both β-arrestin isoforms suggests a shared mode of engagement, although modest isoform-specific differences in functional assays are likely to reflect local dynamics or distal coupling effects. Notably, its overlap with the ICL2-engaging interface of active β-arrestins raises the intriguing possibility that this pocket serves as an allosteric microswitch in β-arrestin activation, highlighting its potential as a tractable site for structure-guided drug discovery. Although this study centres on Cmpd-5, the structural framework established here lays a foundation for exploring how other small molecules modulate β-arrestins through alternative binding modes.

In uncovering small-molecule inhibitors of β-arrestins and delineating their structural and mechanistic basis, this work reveals a previously uncharacterized allosteric pocket that is central to β-arrestin–GPCR engagement. As well as introducing chemical tools to probe β-arrestin biology, these findings signal a conceptual shift from receptor-centric pharmacology towards direct modulation of intracellular transducers, opening a new route to selective regulation of GPCR signalling with pathway-level precision.

## Methods

### Cell culture, antibodies, and reagents

HEK-293 cells (ATCC), including transient and stable lines, as well as CRISPR–Cas9 βARR1/2-knockout and parental lines^61^, were maintained in Eagle’s Minimum Essential Medium (MEM) supplemented with 10% fetal bovine serum (FBS) and 1% penicillin-streptomycin at 37 °C and 5% CO_2_. U2OS cells (DiscoveRx PathHunter) were cultured in MEM containing 2 mM l-glutamine, 10% FBS, and 1% penicillin-streptomycin under similar conditions. U2OS-based β-arrestin recruitment and internalization assays were performed per the manufacturer’s protocol (DiscoveRx). For chemokine-induced migration assays, leukocytes were isolated from wild-type mice as described previously^18,37,61^. *Escherichia coli* strains DH5α and BL21 (DE3) (New England Biolabs) were cultured in LB or Terrific Broth (Fisher Scientific) at 37 °C. DH5α was used for plasmid amplification, and BL21 was used for recombinant protein expression. Sf9 insect cells (*Spodoptera frugiperda*, Expression Systems, 94-001F) were cultured in ESF 921 medium (Expression Systems) at 27 °C. Baculoviruses were generated and amplified according to the manufacturer’s instructions. For serum starvation experiments, cells were cultured in serum-free medium supplemented with 0.1% BSA, 10 mM HEPES, and 1% penicillin-streptomycin. Transfections were performed using FuGene 6 (Promega) or Lipofectamine 3000 (Invitrogen), according to manufacturers’ protocols. All cell lines were confirmed mycoplasma-free by routine testing through the Duke University cell culture facility. Monoclonal anti-Flag M2–horseradish peroxidase (HRP) (A8592) and anti-ERK1/2 (ABS44) antibodies were obtained from Sigma-EMD Millipore. HRP-conjugated secondary antibodies (NA9340-1ML and NA9310-1ML) were purchased from Cytiva. Protease and phosphatase inhibitor tablets (cOmplete, PhosSTOP) were obtained from Roche. Anti–phospho-p44/42 MAPK (Thr202/Tyr204) antibody (9101L) was sourced from Cell Signaling Technology.

### Recombinant protein expression and purification

The expression and purification of β-arrestins have been described previously^44,62–64^. In brief, *E. coli* BL21 (DE3) pLysS cells (New England Biolabs: C2527I) carrying pGEX4T1-*Rattus norvegicus* βARR1 or βARR2 construct, with their C terminus truncated (at amino acid 393 or 394, respectively), were cultured in Terrific Broth (Teknova) medium at 37 °C. After OD_600_ reached 0.6–0.8, the cells were induced with 0.1 mM isopropyl-β-d-thiogalactopyranoside (IPTG) at 18 °C overnight. Bacteria were collected by centrifugation (4,000 rpm), and cell pellets were resuspended in lysis buffer (20 mM HEPES, pH 8, 150 mM NaCl, 10% glycerol, 1 mM EDTA, 0.2 mM dithiothreitol (DTT), 1 mM phenylmethylsulfonyl fluoride, and 1 mM benzamidine) at 4 °C for 1 h. Cell suspensions were sonicated, centrifuged (14,000 rpm, 30 min, 4 °C), and the supernatant was loaded onto pre-equilibrated glutathione–agarose resin (GoldBio). After 3 h of binding at 4 °C, the beads were washed, and thrombin was added for overnight cleavage. The proteins were further purified by anion exchange chromatography followed by size-exclusion chromatography using a Superdex 200 Increase 10/300 GL column on an ÄKTA FPLC system (GE Healthcare). Eluted fractions were analysed by SDS–PAGE, pooled, concentrated using 30 kDa MWCO Amicon Ultra-15 Centrifugal Filter devices, flash-frozen in liquid nitrogen, and stored in aliquots at −80 °C. The same rat βARR1 constructs with mutations (L129A, L129S, L129G, P131A, P133A, E134A, D135A, K138A, C140A, Y249A, C251A, E283A, K284A and R285A) were generated and purified in a similar manner. Recombinant Gα subunits (Gα_s_, Gα_q_ and Gα_i_) were purified as GST fusion proteins using glutathione–agarose affinity chromatography as previously described^65^. The heterotrimeric G_s_ protein complex, composed of Gα_s_, Gβ1 and Gγ2 subunits, was expressed in Sf9 cells using the baculovirus expression system and purified as previously described^66^. M_2_R expression, purification and reconstitution into high-density lipoprotein (HDL) particles were performed as previously described^58^. ERK2, SRC, p38α and JNK3 kinases were expressed and purified using previously established protocols^6,16^. Protein concentrations for each protein were determined by ultraviolet absorption at 280 nm and extinction coefficients estimated using the ExPASy ProtParam tool^67^.

### Expression of β_2_AR constructs in Sf9 cells using the baculoviral system

The N-terminal Flag-tagged T4 lysozyme fusion-β_2_V_2_R construct (β_2_AR residues 1–341 fused to V_2_R residues 328–372), bearing a TEV cleavage site, was co-expressed with GRK2–CAAX (membrane-anchored GRK2) or wild-type β_2_AR in Sf9 insect cells using the Baculovirus Expression System, as described previously^44,62,63,68,69^. At 66 h after infection, cells expressing T4L–β_2_V_2_R were stimulated with 20 μM isoproterenol for 20 min at 37 °C to induce receptor phosphorylation (pβ_2_V_2_R), while β_2_AR-expressing cells remained untreated. All cells were washed extensively to remove residual agonist. For membrane preparation from these cells, briefly, cells were resuspended in cold homogenization buffer (75 mM Tris-HCl, pH 7.4, 2 mM EDTA, and cOmplete protease inhibitor) and collected by centrifugation at 500*g* for 5 min at 4 °C. After 2 additional rounds of centrifugation at 500*g* for 5 min at 4 °C, the supernatant was centrifuged at 21,000*g* for 30 min at 4 °C to collect crude membrane fractions. Pellets were then washed with resuspension buffer (75 mM Tris-HCl, pH 7.4, 2 mM EDTA, 12.5 mM MgCl_2_, cOmplete protease inhibitor, and PhosSTOP phosphatase inhibitor), passed through a 0.4 mm gauge needle 30 times using a syringe on ice, aliquoted, flash-frozen in liquid nitrogen, and stored at −80 °C.

### Small-molecule library and reagents

A collection of structurally diverse, drug-like small-molecule libraries used in this work was obtained from the NCI/DTP Open Chemical Repository. The compound library comprised approximately 3,500 compounds, representing the structural diversity of over 250,000 unique small molecules. This DTP library included the NCI Diversity Set, natural products, and FDA-approved oncogenic drugs. Most compounds were certified as >95% pure by the supplier (NCI DTP Discovery Services). Powdered compounds were dissolved in 100% DMSO and stored at −20 °C. Isoproterenol (Sigma, I2760-1G), ICI-118,551, carvedilol, angiotensin II (ANGII; Sigma, A9525), and human epidermal growth factor (EGF) were purchased from Sigma-Aldrich, and AVP was obtained from GenScript. All small-molecule compounds, including BI-167107 (ref. ^70^), were dissolved in DMSO and stored at −20 °C as 100 mM stock solutions. The C-terminal peptide of the GPCR vasopressin-2 receptor (V_2_R), known as V_2_Rpp, was synthesized by the Tufts University Analytical Core Facility. Cellular agonist stimulations were performed at 37 °C, as described in the figure legends.

### Differential scanning fluorimetry

DSF assay was performed to identify β-arrestin-binding small molecules from the drug-like compound library (DDLC) described above. The screen was conducted using the StepOnePlus Real-Time PCR System (Applied Biosystems) with the fluorescent reporter probe SYPRO Orange (Thermo Fisher Scientific) in a 96-well format. Proteins were buffered in 20 mM HEPES (pH 7.5) with 100 mM NaCl. Small molecules (100 μM) were screened with either βARR1 or βARR2 (5 μM). DMSO was used as a vehicle control, and V_2_Rpp (50 μM) served as a positive control.

For DSF experiments involving the three positive control ligands (V_2_Rpp, IP_6_ and heparin, each at 50 μM) binding to β-arrestins, HEPES buffer was used as the vehicle control. Excitation and emission filters for SYPRO Orange were set to 475 nm and 580 nm, respectively. The temperature was increased by 0.5 °C every 30 s, from 25 °C to 99 °C, with fluorescence readings taken at each interval. Raw DSF data were analysed using Applied Biosystems Protein Thermal Shift Software. Fluorescence intensities were plotted as a function of temperature, and the midpoint of transition, or melting temperature (*T*_m_), was calculated by plotting the first derivative of fluorescence emission as a function of temperature (d*F*/d*T*). The difference between the *T*_m_ of the protein–ligand complex and that of the protein alone represents the thermal shift (Δ*T*_m_), which indicates ligand binding to the protein of interest. For statistical analysis, experiments were conducted with at least three independent replicates per condition. The final selection of compounds for this study was guided by their efficacy in inhibiting βARR1/2 activity, along with favourable chemical properties, such as aqueous solubility and permeability, as described in the Results section. Three compounds were selected as candidate inhibitors: Cmpd-5 (NSC 250682), (1*S*,4a*R*,5*S*,6*S*,6a*R*,9*S*,11a*S*,11b*S*,14*R*)-1,5,6,14-tetrahydroxy−4,4-dimethyl-8-methylenedecahydro-1*H*-6,11b-(epoxymethano)-6a,9-methanocyclohepta[a]naphthalen-7(8*H*)-one; Cmpd-46 (NSC 302979), (*Z*)-3-ethoxy-6-hydroxy-4,4,13a-trimethyl-9-methylene-1,2,3,4,4a,5,6,9,10,11,12,13a-dodecahydro-7,10-(metheno)benzo[11]annulene-8,13-dione; and Cmpd-64 (NSC 22070), (*Z*)-4,10a-dimethyl-7-methylene-8-oxo-1a,2,3,6,6a,7,8,9a,10,10a decahydrooxireno[2’,3’:8,9]cyclodeca[1,2-b]furan-6-yl acetate.

### Measurement of β-arrestin recruitment using the PathHunter assay

β-Arrestin recruitment to the agonist-activated receptor (β_2_V_2_R) was measured using the DiscoveRx PathHunter β-arrestin assay^28^, which uses enzyme fragment complementation. In this assay, the β_2_V_2_R is fused to an inactive portion of β-galactosidase (ProLink tag), and βARR2 is fused to the complementary enzyme acceptor (EA) portion, each stably expressed in U2OS cells. Upon agonist-induced recruitment of βARR2 to β_2_V_2_R, the β-gal fragments complement to form a functional enzyme, generating a measurable chemiluminescent signal. The signal intensity correlates directly with the extent of βARR2 recruitment. U2OS cells co-expressing β_2_V_2_R and βARR2 were plated at a density of 25,000 cells per well in white, clear-bottom 96-well plates 24 h before treatment. On the day of the experiment, cells were treated with either a single dose (50 μM) or varying concentrations of a specific β-arrestin modulator or vehicle control in Hanks’ balanced salt solution (HBSS) (Sigma-Aldrich) with 20 mM HEPES (pH 7.4) and 0.05% BSA. Cells were incubated at 37 °C, 5% CO_2_, and about 100% relative humidity for about 30 min, followed by stimulation with either 10 nM Iso or a serial dilution of Iso for 60 min at 37 °C. After agonist stimulation, PathHunter reagents were added, and cells were incubated for another 60 min at ambient temperature. Luminescence signals were measured using a CLARIOstar microplate reader (BMG Labtech).

### NanoBiT luciferase complementation assays

For each NanoBiT assay, specific components are described below. For NanoBiT-based β-arrestin recruitment assays, CRISPR–Cas9 βARR1/2-knockout HEK293 cells were maintained in MEM supplemented with 10% FBS and 1% penicillin-streptomycin at 37 °C and 5% CO_2_. Cells were seeded in poly-d-lysine-coated white 96-well plates at 100,000 cells per well. β-Arrestin recruitment was monitored using the NanoBiT complementation system, which uses split NanoLuc luciferase fragments (LgBiT and SmBiT). Two assay formats were used. In the first, the LgBiT–CAAX (plasma membrane) and SmBiT–βARR1 format, cells were transfected with 125 ng Flag-tagged GPCR, 25 ng LgBiT–CAAX, 125 ng SmBiT–βARR1, and pcDNA filler using polyethylenimine (PEI) at a 3:1 PEI:DNA ratio. After 24 h, cells were lifted and replated in MEM with 1% FBS. The next day, cells were incubated with 2.5 μM fluorofurimazine and either modulator (50 μM) or DMSO for 20 min at 37 °C. Luminescence was recorded before and after agonist or vehicle addition using a Berthold Mithras LB 940 with a 480 nm filter (NanoLuc). For each condition, data were normalized to the mean baseline and vehicle-stimulated cells. Modulator responses were then normalized to the maximal signal observed in DMSO-pretreated cells stimulated with the highest agonist dose. In the second, the V_2_R–LgBiT and βARR1– or βARR2–SmBiT format, cells were transfected with 25 ng V_2_R–LgBiT and 125 ng βARR1– or βARR2–SmBiT using Lipofectamine 3000 and incubated for 48 h. Cells were washed and treated with coelenterazine-h and modulator (50 μM) or DMSO for 30 min at 37 °C. Baseline luminescence was recorded for 2 min followed by stimulation with 100 nM AVP or vehicle. NanoBiT luminescence was measured for 20 min using a PHERAstar FSX plate reader (BMG LabTech). AUC was used to quantify β-arrestin recruitment normalized to baseline and vehicle. NanoBiT assays between β_2_AR–LgBiT or CCR7–LgBiT and SmBiT–mini-G_i_^71^ were performed similarly as above. HEK293 βARR1/2-knockout cells were seeded into white poly-D-lysine-coated 96-well flat-bottom plates (Corning; 353296). Coelenterazine-h (NanoLight; 301) was added at a final concentration of 10 μM before measurement. After establishing a 3-min baseline, cells were stimulated with 10 μM Iso or 250 nM CCL19 in HBSS to measure mini-G_i_ recruitment in the presence of 50 μM modulator in HEK293 βARR1/2-knockout cells. Luminescence was recorded for 27 min using a CLARIOstar plate reader (BMG LabTech).

### FRET-based cAMP accumulation measurement

To measure cellular cAMP production in live cells mediated by stimulatory G protein, Gα_s_-coupled β_2_AR activation, FRET-based Epac sensors were used as described previously^30^. The Epac2 (ICUE2) sensor contains a CFP and YFP FRET pair. HEK293 cells stably expressing ICUE2 were plated in poly-d-lysine-coated, black, clear-bottom 96-well plates (Corning) at a density of 50,000 cells per well. At least 16 h after plating, cells were washed with PBS and incubated in HEPES-buffered saline solution (10 mM HEPES, 150 mM NaCl, 5 mM KCl, 1.5 mM MgCl_2_, 1.5 mM CaCl_2_, 10 mM glucose, 0.2% BSA, pH 7.4) for 1 h at 37 °C. Cells were then treated with either β-arrestin small-molecule modulators (40 μM) or DMSO for 5 min, and baseline fluorescence was monitored. Real-time cAMP measurement was initiated by stimulating cells with 10 μM Iso at 37 °C. FRET changes corresponding to cAMP accumulation were measured as changes in the background-subtracted 480 nm/535 nm fluorescence emission ratio (CFP/YFP), reflecting changes in cAMP levels. The entire cAMP accumulation profile was quantified by calculating the AUC for the time course. To assess the effect of modulators on the agonist-induced cAMP response, AUC values were expressed as a percentage of the response to Iso in the presence of DMSO (set as 100%), enabling comparison of the effect of each modulator relative to this agonist-alone control.

### Intracellular calcium measurement

Intracellular (Ca^2+^) release was measured using the FLIPR Calcium 6 assay kit with a FlexStation 3 microplate reader, following the manufacturer’s instructions (Molecular Devices) and as described previously^31^. In brief, HEK293 cells stably expressing human angiotensin II type 1 receptor (AT_1_R), parental HEK293 cells transiently expressing AT_1_R, or HEK293 CRISPR–Cas9 βARR1/2-knockout cells transiently expressing AT_1_R were seeded in poly-d-lysine-coated, black 96-well assay plates at a density of 40,000 cells per well and incubated for 24 h. On the day of the experiment, cell plates were loaded with FLIPR Calcium 6 reagents and treated with β-arrestin small-molecule modulators (10 μM) or vehicle (DMSO) for 30 min. After establishing basal fluorescence (*F*_0_), the cells were treated with the agonist ANGII (30 pM for stably expressing AT_1_R or 120 pM for transiently expressing AT_1_R) while fluorescence intensity (*F*) was monitored in real time. These sub-maximal ANGII concentrations were empirically optimized to elicit moderate, temporally resolved Ca^2+^ transients, allowing sufficient time for β-arrestin-mediated desensitization to develop prior to the peak response. The complete Ca^2+^ transient profile was quantified by calculating the AUC over time. AUC values were normalized to the response to ANGII alone (set as 100%) to compare the effects of modulators relative to the agonist-alone control.

### Measurement of receptor internalization by PathHunter assay

β-Arrestin-mediated receptor internalization was measured using the DiscoveRx PathHunter active receptor endocytosis assay according to the manufacturer’s protocol (DiscoveRx) and as previously described^28^. In brief, β_2_V_2_R was transiently transfected into U2OS cells stably expressing an EA-tagged βARR2 and an endosome-localized ProLink-tagged protein. The next day, cells were seeded at 25,000 cells per well in white, clear-bottom 96-well assay plates and incubated for 24 h before the experiment. On the day of the experiment, cells were treated with varying concentrations of a specific β-arrestin modulator or vehicle control in HBSS (Sigma-Aldrich) with 20 mM HEPES (pH 7.4) and 0.05% BSA. Cells were incubated at 37 °C, 5% CO_2_, and ~100% relative humidity for ~30 min, followed by stimulation with a series of concentrations of agonist (Iso) for 60 min at 37 °C. After agonist stimulation, PathHunter reagents were added, and cells were incubated for another 60 min at ambient temperature. Receptor–β-arrestin complex internalization was detected as luminescence resulting from the complementation of β-gal fragments (enzyme acceptor and ProLink) within endosomes. Luminescence signals were measured using a CLARIOstar microplate reader (BMG Labtech).

### BRET-based receptor internalization assay

BRET-based assays were conducted to measure receptor internalization using a bystander BRET format as previously described^33^. In brief, HEK293 cells transiently expressing V2R–RLucII (BRET donor) and the early endosome marker 2×FYVE-mVenus (BRET acceptor) were pretreated with vehicle or β-arrestin modulator (40 μM) for 30 min. Receptor association with the endosome marker was measured as a BRET signal after stimulation with a range of AVP concentrations. BRET measurements were performed using the Synergy2 (BioTek) microplate reader with filter sets of 410/80 nm and 515/30 nm to detect RLucII (donor) and mVenus (acceptor) emissions, respectively. The BRET signal was calculated as the ratio of light intensity emitted by the acceptor over the donor, and the ‘net BRET’ ratio was determined by subtracting the vehicle control ratio from the corresponding AVP-treated ratio.

### G_i_ activity assay

G_i_-mediated inhibition of adenylyl cyclase was assessed using the GloSensor cAMP bioluminescence biosensor (Promega). HEK293 cells were transiently transfected with D_2_R or M_2_R together with the GloSensor cAMP reporter construct, using FuGENE 6 per the manufacturer’s protocol. Cells were seeded into poly-d-lysine-coated white clear-bottom 96-well plates at 60,000 cells per well and cultured for 24 h. GloSensor reagent was added per the manufacturer’s instructions and cells were equilibrated for 1 h at room temperature. Where indicated, pertussis toxin (PTX; 100 ng ml^−1^) was applied overnight as a positive control for G_i_ blockade. Compounds (Cmpd-5, Cmpd-46 or Cmpd-64; 50 μM) or vehicle (DMSO) in HBSS supplemented with 20 mM HEPES (pH 7.4) and 0.05% BSA were applied for 15 min at room temperature. Cells were stimulated with forskolin (2 μM, 5 min), followed by concentration-response stimulation with quinpirole (D_2_R) or acetylcholine (M_2_R). Luminescence was recorded every 5 min over 30 min using a CLARIOstar microplate reader (BMG Labtech). Forskolin-stimulated cAMP, quantified at 10 min after agonist addition, was normalized to the maximal forskolin response per condition. G_i_ activity was derived from the inhibitory plateau and expressed as a percentage of the DMSO plus agonist control.

### Intramolecular FlAsH-BRET assay

Intramolecular FlAsH-BRET assays^72^ were performed in HEK293 βARR1/2-knockout cells transiently transfected using FuGENE4K with 2 μg total DNA per 15 cm dish. Cells were transfected with 1.5 μg of pcDNA3.1 encoding human AT_1_R and 0.4 μg of a plasmid encoding the indicated RLuc–β-arrestin2–FlAsH biosensors. Twenty-four hours after transfection, cells were replated into poly-d-lysine-coated 96-well white, clear-bottom plates at 100,000 cells per well in MEM supplemented with 10% FBS. Forty-eight hours after transfection, cells were labelled with FlAsH-EDT_2_ (FlAsH II In-Cell Tetracysteine Detection Kit, Thermo Fisher) at a final concentration of 2.5 μM for 30 min at room temperature. Following labelling, cells were washed twice with BAL wash buffer (250 μM 2,3-dimercapto-1-propanol in HBSS), then equilibrated in HBSS supplemented with 20 mM HEPES (pH 7.4). Modulators were applied at 50 μM for 15 min, followed by addition of Prolume Purple substrate (Nanolight Technologies). Cells were then stimulated with 10 μM angiotensin II. BRET signals were recorded using a CLARIOstar Plus plate reader in kinetic mode (480 nm donor, 530 nm acceptor) for 30 min, and peak values were used for quantification. ΔNet BRET was calculated by subtracting baseline values from from each condition, including agonist alone, modulator alone, or agonist plus modulator.

### TRUPATH BRET assay

TRUPATH assays^73^ were performed in HEK293 βARR1/2-knockout cells transiently transfected using FuGENE4K with a total of 4 μg per 15 cm dish plasmid DNA, including pcDNA3.1 constructs encoding human GPCRs (β_2_AR, AT_1_R, M_2_R or NTSR1), Gα–RLuc8, Gβ3 and Gγ9–GFP2 at a 1:1:1:1 ratio. TRUPATH components were obtained from B. Roth via Addgene (1000000163). Twenty-four hours after transfection, cells were replated into poly-d-lysine-coated 96-well white, clear-bottom plates at 20,000–30,000 cells per well in phenol red-free DMEM supplemented with 2% FBS. BRET signals were acquired 48 h after transfection. On the day of the assay, cells were washed and incubated in HBSS containing 20 mM HEPES (pH 7.4). Modulators were applied at 50 μM for 15 min, followed by addition of Prolume Purple substrate (Nanolight Technologies). Cells were then stimulated with increasing concentrations of agonist (angiotensin II, neurotensin, isoproterenol or acetylcholine). BRET2 was recorded in kinetic mode for 30 min using a CLARIOstar Plus plate reader (480 nm/510 nm), and peak signals were used for quantification. ΔNet BRET ratios were calculated by subtracting the BRET ratio of the vehicle-treated control from each condition, agonist alone or agonist plus modulator. Data represent the mean of three technical replicates across 3–5 independent experiments and were fitted using a three-parameter logistic model in GraphPad Prism.

### Chemotaxis assays

Chemotaxis assays were performed similarly to previously described protocols^37^. In brief, T cells were isolated from the spleens of wild-type mice, subjected to erythrocyte lysis, and filtered through a 70-μm filter. The cells were then suspended in RPMI 1640 medium containing 0.5% BSA and treated for 30 min with β-arrestin modulators or vehicle. A total of 1 × 10^6^ cells in 100 μl of medium were added to the upper chamber of 6.5-mm diameter, 5-μm pore polycarbonate Transwell filters (Corning Costar), and cells migrated towards 100 nM CCL19 in the lower chamber for 2 h at 37 °C. T cells that migrated to the lower chamber were collected, resuspended, washed, and stained for flow cytometry analysis using a Live/Dead marker (Aqua Dead, Thermo Fisher) and antibodies for cell surface markers (CD45^+^, CD3^+^, CD4^+^ and CD8^+^) prior to paraformaldehyde fixation. The total live T cell population (CD45^+^ and CD3^+^) and subset populations (CD45^+^, CD3^+^ and CD4^+^, or CD45^+^, CD3^+^ and CD8^+^) were measured using a BD LSR Fortessa machine from the Flow Cytometry Shared Resource (FCSR) at the Duke Cancer Institute (Durham, NC). CountBright beads (Thermo Fisher) were added immediately after resuspension of the lower chamber contents to correct for volume differences and any cell loss during wash steps. Per cent migration was calculated by determining the percentage of migrated cells treated with compounds relative to control wells, with migration in CCL19-treated wells alone set as 100%. The use of mouse splenocytes ex vivo for this T cell migration was conducted under institutional guidelines for the care and use of laboratory animals. No live animal procedures were performed. A representative gating tree is shown in Extended Data Fig. 5.

### Adult ventricular cardiomyocyte isolation and contractility analysis

All animal experiments were approved by the Institutional Animal Care and Use Committee (IACUC) at Duke University Medical Center and performed in accordance with relevant guidelines and regulations. Ventricular cardiomyocytes were freshly isolated from 12- to 16-week-old C57BL/6J wild-type mice using a standard Langendorff perfusion system^39,74^. The heart was initially cannulated through the aorta and then perfused with an oxygenated buffer at 37 °C (120 mM NaCl, 14.8 mM KCl, 0.6 mM KH_2_PO_4_, 0.6 mM Na_2_HPO_4_, 1.2 mM MgSO_4_·7H_2_0, 10 mM HEPES, 4.6 mM NaHCO_3_, 30 mM taurine, and 5.6 mM glucose, pH 7.3). After 2 min, the buffer was switched to a digestion solution containing 2.4 mg ml^−1^ collagenase (Worthington). Following 8 min of digestion, ventricles were transferred to a stopping buffer that included 10% calf serum. Myocytes were then manually dissociated and gradually brought to a physiological Ca^2+^ concentration (1.2 mM). Isolated cardiomyocyte was first pretreated with DMSO (0.1%), C5, C46 or C64 (25 µM) for 20 min followed by treatment with HBSS (basal), angiotensin II (positive control, 10 µM) or TRV027 (10 µM). Cells were plated in a chamber (Ionoptix) on a Nikon Eclipse TE300 inverted microscope (40× 0.9 NA objective, MRF00400, Nikon). Cardiomyocytes were paced at 1 Hz and 20 V (MyoPacer, Ionoptix), and sarcomere length was recorded by a MyoCam-S camera (Ionoptix). Ten consecutive contractions per cell (7–12 cells per condition) were averaged to quantify contractility and kinetic parameters (IonWizard 7.2). Only those cells exhibiting proper single cardiomyocyte morphology and responsiveness to electrical stimulation were included. Sample size represents the number of hearts at each condition derived from an average of 7–12 cells per heart for each condition. Statistical comparisons between conditions were performed using one-way ANOVA followed by Sidak post hoc test.

### Isothermal titration calorimetry

ITC measurements were performed on a MicroCal iTC200 system at 25 °C. Prior to ITC experiments, all proteins (βARR1, βARR2 or βARR1 mutants) were extensively dialysed against 20 mM HEPES (pH 7.4), 100 mM NaCl, and 3 μM TCEP. Protein concentrations were determined by spectrophotometry at 280 nm, using extinction coefficients calculated from each protein sequence via the ProtParam program^67^. The dialysis buffer was used to dilute DMSO stock solutions of β-arrestin small-molecule modulators to their final concentrations for measurements. For each ITC experiment, β-arrestin modulators (Cmpd-5 at 350 μM, Cmpd-46 at 450 μM, and Cmpd-64 at 450 μM) were loaded into the syringe and titrated into the calorimetric cell containing βARR1, βARR1 mutants or βARR2 (at about 30–40 μM, depending on the protein and experiment). Additional ITC experiments were also performed using purified GST-fused Gα subunits (Gα_s_, Gα_q_ and Gα_i_; about 30 μM) and Gα_i_βγ heterotrimer (about 30 μM). Gα subunits were prepared in the same buffer (20 mM HEPES, pH 7.4, 100 mM NaCl, 3 μM TCEP), whereas the Gα_i_βγ heterotrimer was maintained in buffer supplemented with 0.05% DDM and 10 μM GDP, with matched DMSO carrier conditions. Modulators (300 μM) or corresponding DMSO controls were loaded and titrated as described above. The reference cell was filled with distilled water. In all experiments, the titration sequence typically consisted of an initial 0.4 μl injection, followed by 19 injections of 2 μl each, with a 180-s interval between injections to allow thermal power to return to baseline. During the experiment, the reference power was set to 7 μcal s^−1^, and the sample cell was stirred continuously at 750 rpm. Raw data, excluding the first injection, were baseline-corrected, integrated, and normalized. Data were analysed and fit using a one-site independent binding model to obtain the equilibrium dissociation constant, *K*_d_ (from the association constant *K*_a_ = 1/*K*_d_), stoichiometry (*N*), and thermodynamic parameters including enthalpy (Δ*H*) and entropy (−*T*Δ*S*) of binding. Data were analysed using MicroCal PEAQ-ITC analysis software (v1.1.0.1262).

### Radioligand-binding experiments

To assess the effects of β-arrestin modulators on β-arrestin- or Gα_s_–βγ-promoted high-affinity agonist binding, [^3^H]-Fen binding assays were performed using membranes from Sf9 cells expressing phosphorylated β_2_V_2_R (pβ_2_V_2_R) or β_2_AR^28^. Membranes were prepared from Sf9 cells co-expressing Flag-tagged T4L–β_2_V_2_R and GRK2–CAAX under agonist stimulation, or unstimulated β_2_AR alone as described above^28^. Reactions (150 μl) contained 6 nM [^3^H]-Fen (12.6 Ci mmol^−1^), pβ_2_V_2_R membranes, βARR1/2 or βARR1 mutants (2 μM), and β-arrestin modulators (100 μM) or vehicle in HN100 buffer (20 mM HEPES, pH 7.4, 100 mM NaCl, 12.5 mM MgCl_2_). For Gα_s_–βγ assays, β_2_AR membranes were incubated with 4.3 nM [^3^H]-Fen and Gα_s_–βγ heterotrimer (200 nM) in G protein assay buffer with or without modulators. Nonspecific binding was defined using propranolol (25 μM). Following 90 min incubation at room temperature, reactions were filtered onto PEI-soaked GF/B filters and washed with cold buffer. Bound [^3^H]-Fen was extracted overnight in scintillation fluid and quantified by scintillation counting. Specific binding was calculated by subtracting nonspecific from total binding.

### Live-cell ERK1/2 activation assay

To evaluate the effect of small-molecule modulators on β-arrestin-dependent ERK1/2 signalling, HEK293 cells stably expressing β_2_AR were serum-starved for 6 h (refs. ^34,75^), then pretreated with β-arrestin modulators (30 μM) or vehicle for 20 min at 37 °C. Cells were subsequently stimulated with carvedilol (10 μM, 5 min), a β-arrestin-biased agonist known to promote ERK1/2 phosphorylation via β-arrestin scaffolding of RAF–MEK–ERK signalling components. Cells were lysed, sonicated (15 s), and centrifuged at 14,000*g* for 15 min at 4 °C. Equal amounts of total protein were resolved by SDS–PAGE on 4–20% Tris-Glycine gels (Thermo Fisher Scientific), transferred to nitrocellulose or PVDF membranes, and immunoblotted with anti–phospho-ERK1/2 (1:2,000; Cell Signaling) and total ERK1/2 (1:10,000; Millipore Sigma). HRP-conjugated secondary antibodies were used for detection. Protein bands were visualized using Pierce SuperSignal West Pico ECL substrate (Thermo Fisher Scientific), imaged on a ChemiDoc XRS system (Bio-Rad) and quantified by densitometry using ImageLab (Bio-Rad). Data were analysed using GraphPad Prism.

### Pulldown analysis of β-arrestin–effector interactions

To assess the effects of the compounds on βARR1 interactions with its effector proteins SRC and JNK3, βARR1–Flag (5 μM) was incubated with a 20-fold molar excess of Cmpd-5, Cmpd-46 or Cmpd-64 for 30 min at room temperature. Subsequently, 15 μM of SRC or JNK3 was added. To evaluate compound effects on βARR1–ERK2 binding, βARR1 (15 μM) was pre-incubated with a 20-fold molar excess of Cmpd-5, Cmpd-46 or Cmpd-64 for 30 min at room temperature, followed by the addition of 5 μM ERK2–Flag. Afterward, 50 μl of anti-Flag M2 affinity gel (Millipore Sigma) was added, and the mixtures were incubated for 1 h at room temperature with rotation. The resin was then collected by centrifugation and washed 3 times with 1 ml of 20 mM HEPES (pH 7.5), 100 mM NaCl. Bound proteins were eluted using 0.2 mg ml^−1^ Flag peptide in 20 mM HEPES (pH 7.5), 100 mM NaCl, mixed with Laemmli sample buffer (Bio-Rad), and analysed by SDS–PAGE followed by western blotting. Detection was performed using monoclonal anti-Flag M2-peroxidase (HRP) antibody (1:2,000, Sigma-Aldrich A8592, RRID:AB_439702) for βARR1–Flag and ERK2–Flag; polyclonal A1CT antibody^76^ (1:5,000) for βARR1; anti-SRC monoclonal antibody (1:10,000, EMD Millipore 05-184, RRID:AB_2302631) for SRC; and anti-JNK3 monoclonal antibody (1:5,000, Cell Signaling 2305, RRID:AB_2281744) for JNK3. Western blot images were taken using Bio-Rad ChemiDoc system and the densitometry analysis was performed by ImageLab v6.1 and statistical differences were determined by one-way ANOVA with Dunnett’s post hoc test in GraphPad Prism software.

### G protein GTPase assay

GTPase activity of purified heterotrimeric G_i_ protein was measured in vitro using the GTPase-Glo assay (Promega). HDL-reconstituted M_2_R (100 nM) was pre-incubated with iperoxo (10 μM) or DMSO for 15 min at room temperature in assay buffer (20 mM HEPES, pH 7.4, 100 mM NaCl, 10 mM MgCl_2_). β-arrestin modulators (Cmpd-5, Cmpd-46 or Cmpd-64; 10 μM each) or DMSO control were subsequently added together with G_i_ heterotrimer (500 nM) and GTP (2.5 μM), and reactions were incubated for 1 h at room temperature in the continued presence of iperoxo or DMSO. Reactions were terminated by addition of GTPase-Glo reagent, followed by detection reagent, according to the manufacturer’s instructions. Luminescence was measured using a CLARIOstar plate reader (BMG Labtech).

### Cytotoxicity assay

MTT assay was performed according to the manufacturer’s instructions (Roche Diagnostics). HEK293 and U2OS cells were seeded in 96-well plates. The following day, cells were treated with the indicated concentration of modulator or vehicle for 8 h. To evaluate the cytotoxic effects of the compounds, cells were incubated with 3-(4,5-dimethylthiazol-2-yl)-2,5-diphenyltetrazolium bromide (MTT) reagent at 37 °C for 4 h. The optical density was measured at 595 nm (the absorbance of each sample was measured at 560 and 670 nm). The optical density values of blue formazan formed in live cells based on the reduction of MTT were determined at 595 nm. Cell viability was expressed as the percentage of MTT reduction in compound-treated cells compared to vehicle-treated cells.

### Cryo-EM sample preparation and data acquisition

The expression construct for βARR1–BRIL was designed by inserting the thermostabilized apocytochrome b562 (M7W, H102I, K106L) from *E. coli* (BRIL) between residues 176–182 at the hinge region of rat βARR1 (ref. ^64^) truncated at residue 393 and purified as wild-type βARR1. βARR1–BRIL was incubated with 1.5-fold molar excess of anti-BRIL Fab (BAG2)^42^ and twofold molar excess of aFabNb^43^ for 1 h at room temperature. The complex was subjected to size-exclusion chromatography on a Superdex 200 Increase column (Cytiva Life Sciences) in 20 mM HEPES 7.5, 150 mM NaCl buffer. Peak fractions were concentrated to 2 mg ml^−1^ using Vivaspin 6 column with molecular weight cut-off of 30,000 kDa (Sartorius). The complex was incubated for 2 h on ice with 50-fold molar excess of Cmpd-5 solubilized in DMSO (βARR1–BRIL–BAG2–aFabNB–Cmpd-5) or equivalent amount of DMSO (βARR1–BRIL–BAG2–aFabNB–Apo), and then concentrated to 7–8 mg ml^−1^. The sample was applied to glow-discharged 300-mesh holey-carbon grids (Quantifoil R1.2/1.3, Electron Microscopy Sciences) using a Vitrobot Mark IV (Thermo Fisher Scientific) at 4 °C and 100% humidity. The data were collected on a Titan Krios transmission electron microscope (Thermo Fisher) operating at 300 kV equipped with a K3 direct electron detector (Gatan) in counting mode with a BioQuantum GIF energy filter (slit width of 20 eV) at a magnification of 81,000× corresponding to a pixel size of 1.08 Å at the specimen level. Sixty-frame movies with a dose rate of about 15 e^−^ pixel^−1^ s^−1^ and a total accumulated dose of about 54–60 e^−^ Å^−2^ were collected using the Latitude-S (Gatan) single-particle data acquisition program. The nominal defocus values were set from −0.8 to −2.5 µm.

### Cryo-EM data processing

Movies were subjected to beam-induced motion correction using Patch Motion Correction in CryoSPARC v4.0.1 (ref. ^77^) followed by determination of contrast transfer function parameters in Patch CTF. Micrographs with contrast transfer function fit better than 3.5 Å were used for further analysis. Particles manually selected from 15 micrographs were used to train a model in the particle picking tool Topaz v0.2.5a^78^. The trained model was used to pick particles in all micrographs generating 1,572,529 particle projections for βARR1–BRIL–BAG2–aFabNB–Cmpd-5 and 1,307,318 particle projections for βARR1–BRIL–BAG2–aFabNB–Apo. The particles were rescaled to the pixel size of 1.3824 Å for further processing. A subset of particles (500,000) was subjected to ab initio model generation with 5 classes. All particles were then subjected to five rounds of heterogeneous refinement. The resulting particle stacks (396,911 particles for βARR1–BRIL–BAG2–aFabNB–Cmpd-5 and 832,520 particles for βARR1–BRIL–BAG2–aFabNB–Apo) were used in non-uniform refinement and local refinement with a mask excluding aFabNb and the constant region of BAG2. Then particles were subjected to 3D classification without alignment in CryoSPARC with a mask excluding aFabNb and the constant region of BAG2. Finally, the best classes with 177,430 particles (βARR1–BRIL–BAG2–aFabNB–Cmpd-5) and 479,224 particles (βARR1–BRIL–BAG2–aFabNB–Apo) were subjected to another round of local refinement in CryoSPARC, generating a map with a global resolution of 3.47 Å and 3.52 Å, respectively. The cryo-EM maps were post-processed using DeepEMhancer with highRes deep learning model^79^.

### Model building and refinement

The initial models were built manually by fitting the crystal structure of βARR1 (PDB: 1G4M)^80^ into the experimental electron densities using UCSF Chimera v1.15 (ref. ^81^). The BRIL–BAG2–aFabNb part of the model was derived from the cryo-EM structure of Frizzled5–BRIL–BAG2–aFabNb (PDB: 6WW2)^82^. The structures were refined by combining manual adjustments in Coot v0.9.8.3 (ref. ^83^) and ISOLDE^84^ in UCSF ChimeraX 1.6.1 (ref. ^81^), followed by real-space refinement in PHENIX v1.20.1-4487 (ref. ^85^) with Ramachandran, rotamer, torsion and secondary structure restraints enforced. The models were validated with MolProbity v4.5.1 (ref. ^86^). Difference density maps between the Cmpd-5-bound and apo states were calculated using a local scaling-based density difference approach^45^. Structural superpositions and pairwise r.m.s.d. and *Q*-score calculations were performed using the PDBeFold server^87^. Cryo-EM refinement statistics are summarized in Extended Data Table 1.

### Modelling and molecular dynamics simulations

The apo βARR1 model in the basal state and the β_2_V_2_R–βARR1 complex (PDB code: 6TKO)^60^ model were built and simulated as described previously^88^. Due to a considerable number of missing residues in the Cmpd-5-bound βARR1 cryo-EM structure, we constructed a Cmpd-5-bound βARR1 model by placing Cmpd-5 in the MCL cleft of an equilibrated basal βARR1 model according to the experimentally derived location of the bound Cmpd-5. A βARR1 model in the active state was constructed by homology modelling with Modeller (v10.0)^89^ using the crystal structure of the V_2_Rpp-bound βARR1 in the active state (PDB code: 4JQI)^44^ as the template. Parts without any template—that is, residues 1 to 5 and 307 to 311—were ab initio modelled with Modeller. The resulting model with the lowest DOPE score was selected for the following steps. The selected βARR1 models were processed by the Protein Preparation Wizard of Schrodinger Suite (v2023-1). The first and last residues of this model are in positively and negatively charged states, respectively, without capping, as assumed in their natural condition. The prepared model was then immersed in a simulation water box using the System Builder of Schrodinger Suite (v2023-1). A simple point charge water model was used to solvate the system, and Na^+^ and Cl^−^ ions were added to neutralize the system and the salt concentration of the system was increased to 0.15 M. The total system size was about 148,000 atoms.

Molecular dynamics simulations were carried out using Desmond MD System (v6.1; D.E. Shaw Research)^90^ with the OPLS4 force field^91^. The simulation systems were minimized and equilibrated with restraints on the ligand heavy atoms and the protein backbone. For both the equilibrations and the following production runs, the constant temperature 310 K was maintained by Langevin dynamics, 1 atm constant pressure was achieved with the Langevin piston method^92^. A cut-off distance of 9 Å was used for the nonbonded interactions, and the particle-mesh Ewald summation method was used for the electrostatics interactions. The integration timestep was set to 2.5 fs. The isothermal-isobaric (NPT) ensemble was used in a periodic boundary condition. The production runs of β-arrestin simulations are without any restraints. The analysis and visualization were performed with VMD^93^ and PyMol (Schrodinger).

### Absolute protein–ligand binding free energy perturbation calculations

We used absolute protein–ligand binding free energy perturbation (AB-FEP) approach as implemented within Schrodinger suite (release 2025-2)^94^ which is based on the originally proposed double decoupling scheme^95^. In this method, starting from the ligand in the water, the van der Waals and electrostatic interactions within the ligand, and between the ligand and water, were first gradually turned off, yielding a dummy ligand. The dummy ligand was then restrained to the protein binding site through a set of cross-link restraints. Finally, the van der Waals and electrostatic interactions within the ligand, and between the ligand and protein, were gradually turned on, while the cross-linked restraints were relaxed. The starting point for the AB-FEP run was a representative frame from the trajectory shown in Supplementary Videos 1 and 2.

### Molecular docking of β-arrestin modulators

Molecular docking was performed to evaluate the binding of Cmpd-5, Cmpd-46 and Cmpd-64 at the βARR1 allosteric site identified in the cryo-EM structure of the βARR1–Cmpd-5 complex. The βARR1 structure was prepared from the cryo-EM model, with the Cmpd-5 binding site defined by residues forming the middle loop, lariat loop and C-loop. Docking was carried out using Schrödinger Maestro (suite 2025-2). Ligands were prepared using LigPrep with ionization states assigned for physiological pH. A receptor grid was generated centred on the Cmpd-5-binding pocket. Cmpd-5 docked with a pose that closely aligned to the experimentally resolved conformation, validating the identified binding pocket. By contrast, Cmpd-46 and Cmpd-64 produced lower-scoring poses with less favourable shape and contact complementarity, consistent with their reduced binding and functional activity.

### Graphing and statistical analyses

All graphs were generated and analysed using GraphPad Prism 10.0 (GraphPad Software). Dose–response curves were fitted to a log(agonist) versus response model with parameters for span, baseline, and half-maximal effective concentration (EC_50_), and minimum baseline was corrected to zero. For statistical comparisons, one-way ANOVA with Dunnett’s post hoc test was generally used for comparisons across more than two groups, and two-way ANOVA with Sidak’s post hoc test or similar was used for comparisons involving multiple conditions, as specified in the figure legends. Most experiments were conducted with three biological replicates, and additional replicates served as controls. Replicates in the figure legends refer to biological replicates, with technical replicates included in some experiments for intra-replicate variation. Differences with *P* values < 0.05 were considered significant. Further statistical details and replicate information are provided in the figure legends.

### Inclusion and ethics statement

All authors meet the authorship criteria required by Nature Portfolio journals and contributed meaningfully to the study. Authorship was determined collaboratively and was not influenced by gender, seniority, or institutional affiliation. Roles were agreed upon in advance, and the work was conducted responsibly and ethically, following institutional standards and inclusive research practices.

### Reporting summary

Further information on research design is available in the Nature Portfolio Reporting Summary linked to this article.

## Online content

Any methods, additional references, Nature Portfolio reporting summaries, source data, extended data, supplementary information, acknowledgements, peer review information; details of author contributions and competing interests; and statements of data and code availability are available at 10.1038/s41586-026-10683-5.

## Supplementary information

Supplementary Information Supplementary Figs. 1–4 and Supplementary Table 1

Reporting Summary

Peer Review file

Supplementary Video 1 Stable binding of Cmpd-5 to the MCL site of βarr1

Supplementary Video 2 Stable interactions between Cmpd-5 and MCL site residues

## Figures and Tables

**Extended Data Table 1:**
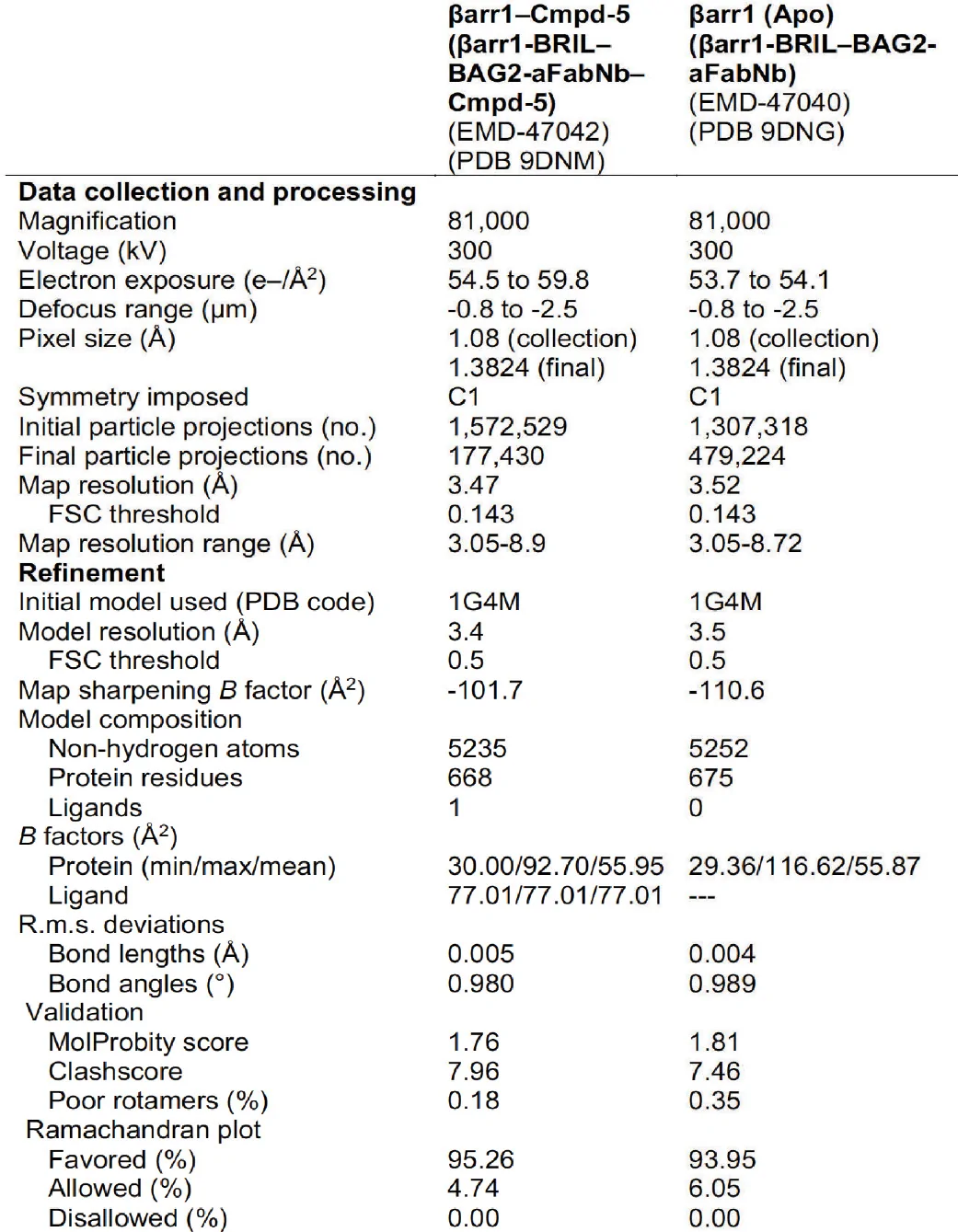
Cryo-EM data collection, refinement, and validation statistics

**Extended Data Table 2:**
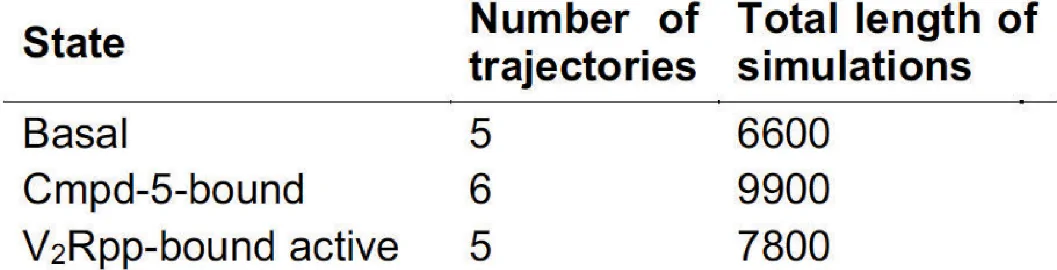
Summary of molecular dynamics simulations

## Data Availability

All data supporting the findings of this study are included in the Article, the Extended Data, the Supplementary Information (including Supplementary Tables) or the Supplementary Videos. Coordinates and cryo-EM maps have been deposited in the Protein Data Bank (PDB) and the Electron Microscopy Data Bank (EMDB) under accession codes 9DNM and EMD-47042 for βARR1–Cmpd-5, and 9DNG and EMD−47040 for apo βARR1. Additional data and materials, including those supporting the supplementary information, are available upon request.
