# Supplementary material for: Small-molecule modulation of β-arrestins
Source: Nature. 2026 Jun 24;656(8128):770–9. doi: 10.1038/s41586-026-10683-5 (PMC13489962; doi:10.1038/s41586-026-10683-5)
Supplement: Supplementary file 1 [file 41586_2026_10683_MOESM1_ESM.pdf]

nature portfolio
https://doi.org/10.1038/s41586-026-10683-5

Supplementary information

Small-molecule modulation of β-arrestins

In the format provided by the
authors and unedited

Supplementary information for

Small-molecule modulation of β-arrestins

Alem W. Kahsai[1,#,*], Natalia Pakharukova[1,#], Henry Y. Kwon[1,7], Kunal S. Shah[1,5,8], Caroline T. del Real[1], Bowie N. Shreiber[1], Jason G. Liang-Lin[1], Paul J. Shim[1], Mason A. Lee[1], Van A. Ngo[9], Allison M. Schwalb[1,5], Uyen Pham[1,2], Anand Chundi[1,5], Haoran Jiang[1,5], Emmanuel Flores-Espinoza[10], Samuel Liu[1], Preston C. Nibley[1,3], Dana K. Bassford[1], Hyunggu Hahn[10], Cal A. Kunzle[1], Brittany N. Thomas[1], Jihee Kim[1], Yang Zhou[3], Jialu Wang[1,3], Xingdong Zhang[1], Jeffrey S. Smith[1,11], Lindsay A.M. Rein[1], Alex R. B. Thomsen[10], Sudha K. Shenoy[1,3], Sudarshan Rajagopal[1,2], Lei Shi[12], Seungkirl Ahn[1], Howard A. Rockman[1,3], Ali Masoudi[1], Robert J. Lefkowitz[1,2,4,6,*]

Departments of [1]Medicine, [2]Biochemistry, [3]Cell Biology, and [4]Chemistry, and [5]Duke University School of Medicine and the [6]Howard Hughes Medical Institute, Duke University Medical Center, Durham, NC, 27710, USA.

[7] Department of Surgery, Henry Ford Hospital, Detroit, MI, 48202, USA.

[8]Cedars-Sinai Medical Center, Los Angeles, CA 90048, USA.

[9]U.S. Department of Energy, National Center for Computational Sciences, Oak Ridge National Laboratory, Oak Ridge, TN 37830, USA.

[10]Department of Molecular Pathobiology, New York University College of Dentistry, New York, NY, 10010, USA.

[11]Departments of Dermatology, Massachusetts General Hospital and Harvard Medical School, Boston, MA 02114–02115, USA.

[12]Computational Chemistry and Molecular Biophysics Section, National Institute on Drug Abuse – Intramural Research Program, National Institutes of Health, Baltimore, MD 21224, USA.

Author notes: # A.W.K. and N.P. contributed equally to this work.

* Corresponding authors: alem@receptor-biol.duke.edu (A.W.K.) and lefko001@receptor-biol.duke.edu (R.J.L.)

Table of Contents:

Supplementary Figure 1. Uncropped blot source data for Fig. 3b ………………………… p. 3
Supplementary Figure 2. Uncropped blot source data for Extended Data Fig. 7a ……… p. 4
Supplementary Figure 3. Uncropped blot source data for Extended Data Fig. 7b ……… p. 5
Supplementary Figure 4. Uncropped gel source data for Extended Data Fig. 11a ……… p. 6
Supplementary Table 1. Oligonucleotide primers for βarr1–Cmpd-5 site point mutations p. 7
Supplementary Video 1. Stable binding of Cmpd-5 to the MCL site of βarr1
Supplementary Video 2. Stable interactions between Cmpd-5 and MCL site residues

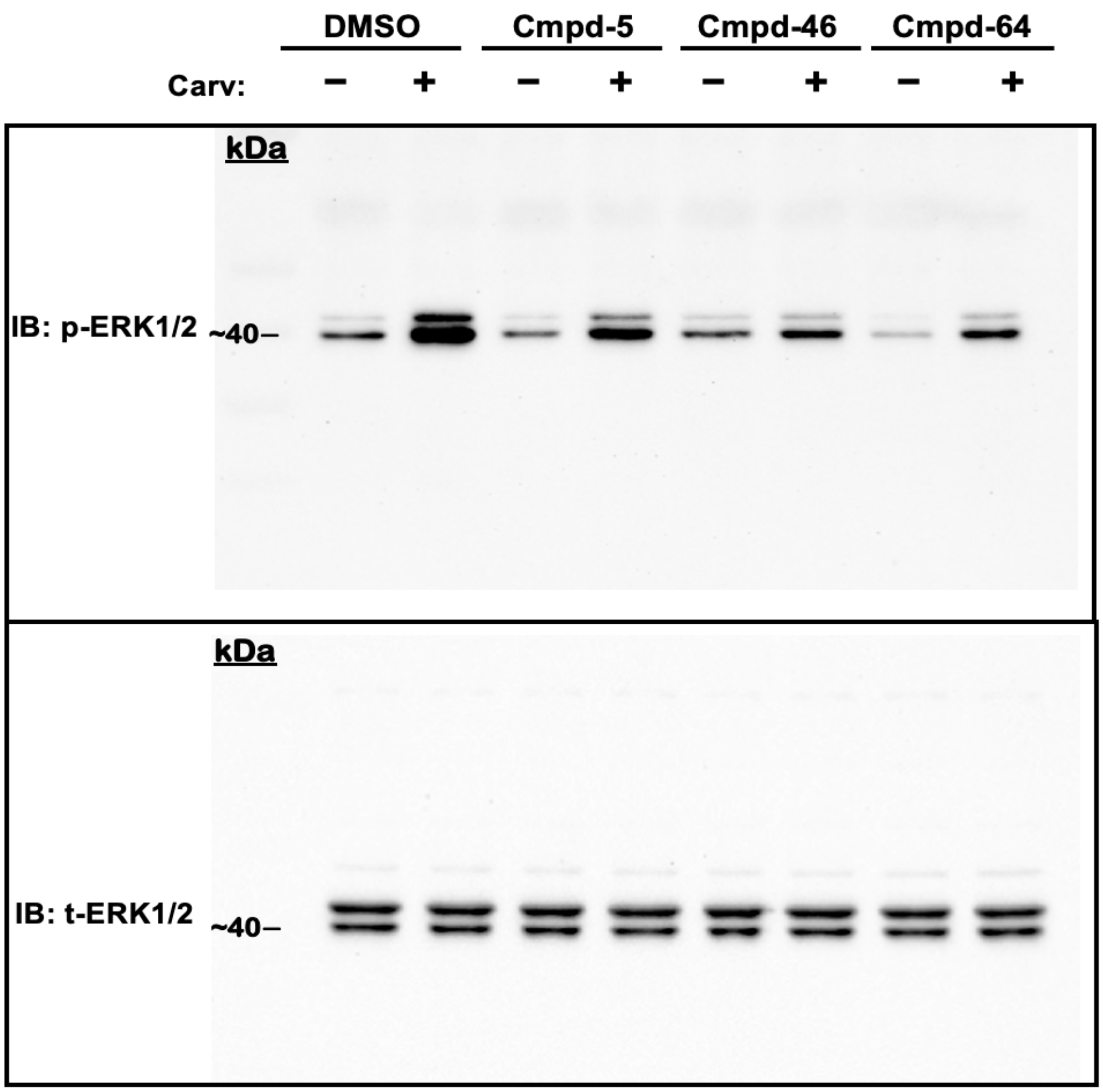

Supplementary Figure 1. Uncropped blot source data for Fig. 3b. Full scans of western blots showing IB: p-ERK1/2 (top) and IB: t-ERK1/2 (bottom) from β2AR-expressing HEK293 cells pretreated with Cmpd-5, -46, or -64 and stimulated with carvedilol (Carv). pERK1/2 and t-ERK1/2 were run on separate gels. Molecular weight marker (~40 kDa) is indicated.

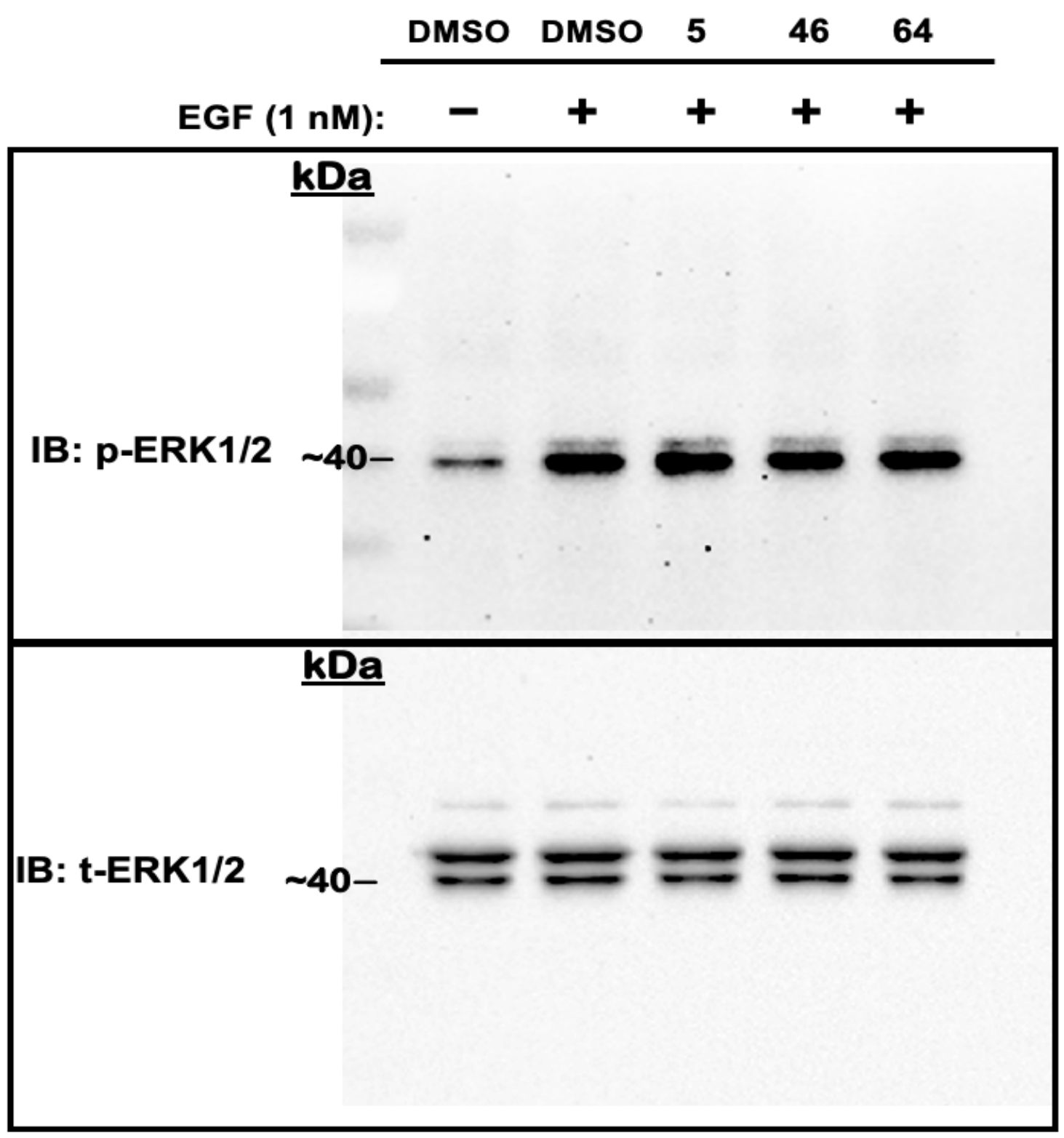

Supplementary Figure 2. Uncropped blot source data for Extended Data Fig. 7a. Full scans of western blots showing IB: p-ERK1/2 (top) and IB: t-ERK1/2 (bottom) from HEK293 cells pretreated with Cmpd-5, -46, or -64 (30 μM) and stimulated with EGF (1 nM). p-ERK1/2 and t-ERK1/2 were run on separate gels. Molecular weight marker (~40 kDa) is indicated.

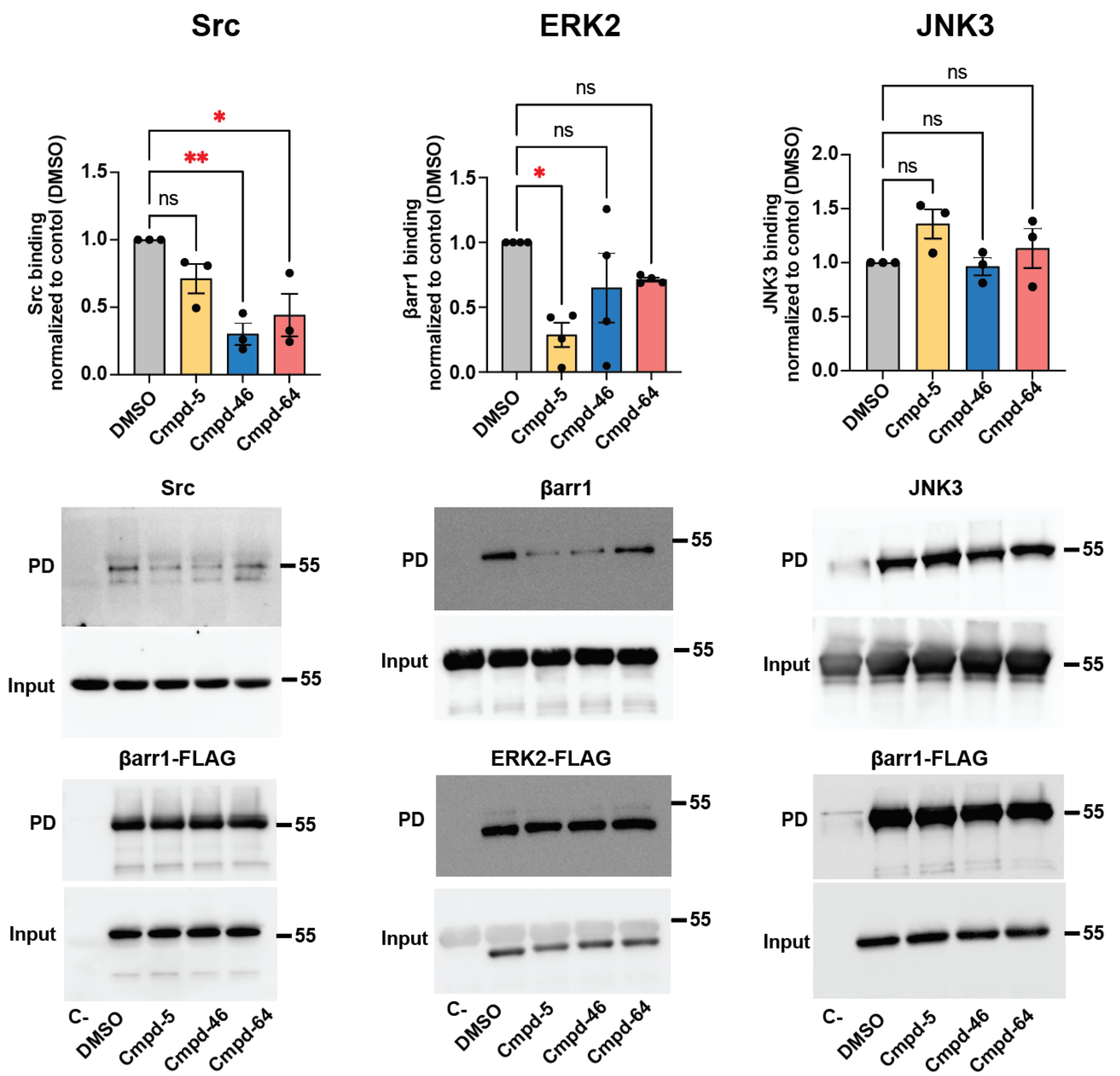

Supplementary Figure 3. Uncropped blot source data for Extended Data Fig. 7b. Full scans of FLAG pull-down immunoblots showing βarr1 interactions with Src, ERK2, and JNK3 in the presence of Cmpd-5, -46, or -64. For each effector, pull-down (PD) and input fractions are shown for both the effector and βarr1-FLAG (or ERK2-FLAG).

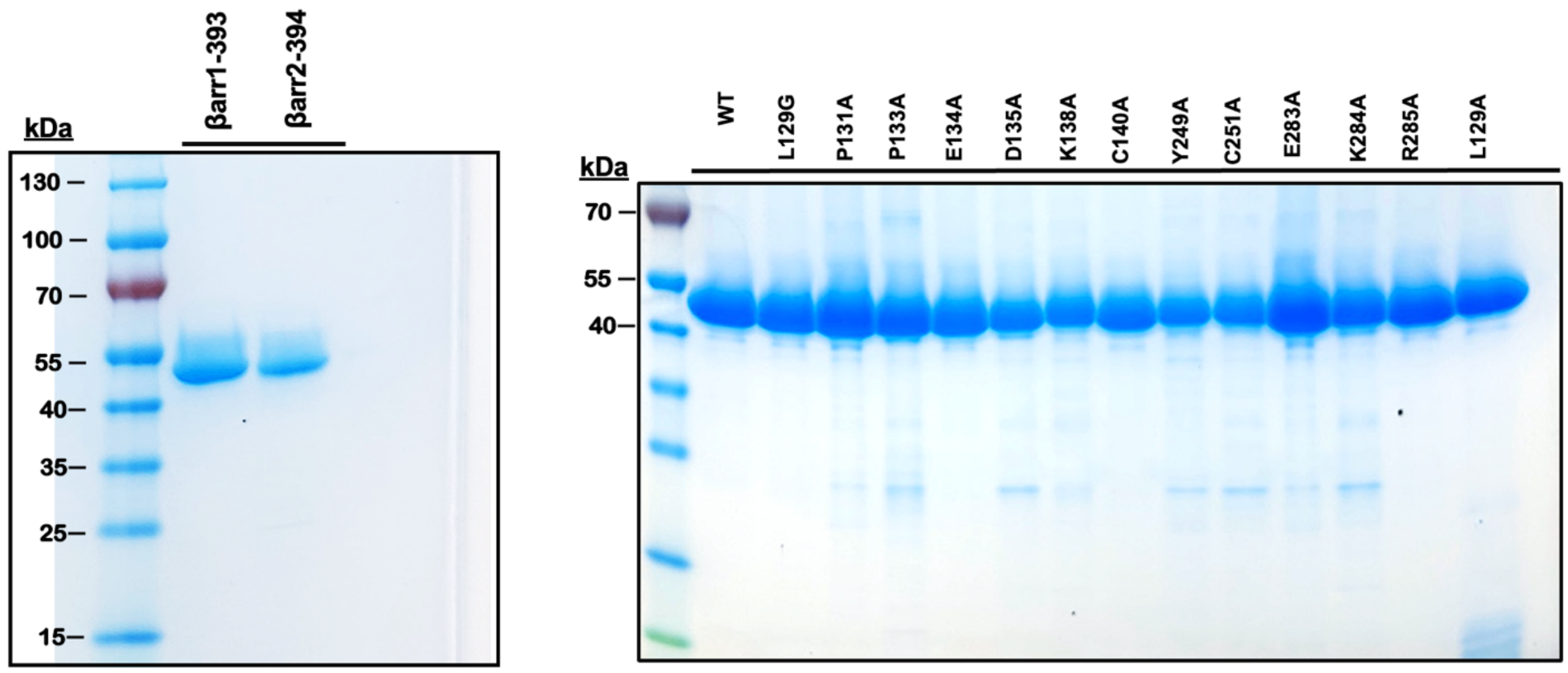

Supplementary Figure 4. Uncropped gel source data for Extended Data Fig. 11a. Full scans of Coomassie-stained SDS–PAGE gels showing purified recombinant βarr1-393 and βarr2-394 (left) and βarr1 point mutants (WT, L129G, P131A, P133A, E134A, D135A, K138A, C140A, Y249A, C251A, E283A, K284A, R285A, and L129A; right). Molecular weights estimated using PageRuler™ Prestained Protein Ladder.

Supplementary Table 1 | Oligonucleotide primers for βarr1–Cmpd-5 site point mutations

Oligonucleotides	
**Primer name**	**Sequence (5' to 3')**
βarr1-L129A_F	GCCCAGGTTGCGCTGTGACTGAGCACGGAAGGTT
βarr1-L129A_R	AACCTTCCGTGCTCAGTCACAGCGCAACCTGGGC
βarr1-L129S_F	GGCCCAGGTTGCGATGTGACTGAGCACG
βarr1-L129S_R	CGTGCTCAGTCACATCGCAACCTGGGCC
βarr1- L129G_F	GCCCAGGTTGCCCTGTGACTGAGCACGGAAGGTT
βarr1-L129G_R	AACCTTCCGTGCTCAGTCACAGGGCAACCTGGGC
βarr1-P131A_F	CTCAGGCCCAGCTTGCAATGTGACTGAGCAC
βarr1-P131A_R	GTGCTCAGTCACATTGCAAGCTGGGCCTGAG
βarr1-P133A_F	CCCTGTGTCCTCAGCCCCAGGTTGCAAT
βarr1-P133A_R	CATTGCAACCTGGGGCTGAGGACACAGGG-
βarr1-E134A_F	CTTCCCTGTGTCCGCAGGCCCAGGTTG
βarr1-E134A_R	CAACCTGGGCCTGCGGACACAGGGAAG
βarr1-D135A_F	GCCTTCCCTGTGGCCTCAGGCCCAG
βarr1-D135A_R	CTGGGCCTGAGGCCACAGGGAAGGC
βarr1-K138A_F	CCACACCGCAGGCCGCCCCTGTGTCCTCAG
βarr1-K138A_R	CTGAGGACACAGGGGCGGCCTGCGGTGTGG
βarr1-C140A_F	TTCATAATCCACACCGGCGGCCTTCCCTGTGTCC
βarr1-C140A_R	GGACACAGGGAAGGCCGCCGGTGTGGATTATGAA
βarr1-Y249A_F	GGCCACTGGGCACTTGGCCTGAGCTGTGTTGAAG
βarr1-Y249A_R	CTTCAACACAGCTCAGGCCAAGTGCCCAGTGGCC
βarr1-C251A_F	CTCCATGGCCACTGGGGCCTTGTACTGAGCTGTG
βarr1-C251A_R	CACAGCTCAGTACAAGGCCCCAGTGGCCATGGAG
βarr1-E283A_F	CAAGCCCCCGCTTCGCTCTGTTGTTTGCCA
βarr1-E283A_R	TGGCAAACAACAGAGCGAAGCGGGGGCTTG
βarr1-K284A_F	AGGGCAAGCCCCCGCGCCTCTCTGTTGTTTGC
βarr1-K284A_R	GCAAACAACAGAGAGGCGCGGGGGCTTGCCCT
βarr1-R285A_F	CGAGGGCAAGCCCCGCCTTCTCTCTGTTGT
βarr1-R285A_R	ACAACAGAGAGAAGGCGGGGCTTGCCCTCG

Supplementary Video 1. Stable binding of Cmpd-5 to the MCL site of β-arrestin1. This video shows the stable binding of Cmpd-5 at the MCL site of βarr1, based on a 3.0 μs molecular dynamics simulation trajectory. βarr1 is shown in wheat, with the MCL region highlighted in orange. Cmpd-5 is represented as spheres, with carbon atoms colored cyan.

Supplementary Video 2. Stable interactions between Cmpd-5 and MCL site residues. This video illustrates key interactions between Cmpd-5 and residues within the MCL site, using the same color scheme as Supplementary Video 1. Interacting residues (L129, P131, P133, E134, Y249, C251, and R285) are shown as sticks.
