# Supplementary material for: Small-molecule modulation of β-arrestins
Source: Nature. 2026 Jun 24;656(8128):770–9. doi: 10.1038/s41586-026-10683-5 (PMC13489962; doi:10.1038/s41586-026-10683-5)
Supplement: Supplementary file 2 [file 41586_2026_10683_MOESM2_ESM.pdf]

nature portfolio

Corresponding author(s): Alem W. Kahsai and Robert J. Lefkowitz

Last updated by author(s): 04/20/2026

Reporting Summary

Nature Portfolio wishes to improve the reproducibility of the work that we publish. This form provides structure for consistency and transparency in reporting. For further information on Nature Portfolio policies, see our Editorial Policies and the Editorial Policy Checklist.

Statistics

For all statistical analyses, confirm that the following items are present in the figure legend, table legend, main text, or Methods section.

n/a	Confirmed	
☐	☒	The exact sample size (*n*) for each experimental group/condition, given as a discrete number and unit of measurement
☐	☒	A statement on whether measurements were taken from distinct samples or whether the same sample was measured repeatedly
☐	☒	The statistical test(s) used AND whether they are one- or two-sided<br>*Only common tests should be described solely by name; describe more complex techniques in the Methods section.*
☒	☐	A description of all covariates tested
☒	☐	A description of any assumptions or corrections, such as tests of normality and adjustment for multiple comparisons
☐	☒	A full description of the statistical parameters including central tendency (e.g. means) or other basic estimates (e.g. regression coefficient) AND variation (e.g. standard deviation) or associated estimates of uncertainty (e.g. confidence intervals)
☐	☒	For null hypothesis testing, the test statistic (e.g. *F*, *t*, *r*) with confidence intervals, effect sizes, degrees of freedom and *P* value noted<br>*Give P values as exact values whenever suitable.*
☒	☐	For Bayesian analysis, information on the choice of priors and Markov chain Monte Carlo settings
☒	☐	For hierarchical and complex designs, identification of the appropriate level for tests and full reporting of outcomes
☒	☐	Estimates of effect sizes (e.g. Cohen's *d*, Pearson's *r*), indicating how they were calculated

Our web collection on statistics for biologists contains articles on many of the points above.

Software and code

Policy information about availability of computer code

Data collection	Cryo-EM data was collected on the ThermoFisher Krios G3i Cryo TEM at the Duke University Shared Materials Instrumentation Facility (SMIF), a member of the North Carolina Research Triangle Nanotechnology Network (RTNN), which is supported by the National Science Foundation (award number ECCS-2025064) as part of the National Nanotechnology Coordinated Infrastructure (NNCI). Data for Cryo-EM imaging was collected in automated mode, using Latitude-S Software. Data for DSF experiments was collected on the StepOnePlus RT-PCR system (Thermo Fisher Scientific; Cat# 4376600; RRID:SCR_015805) using Applied BiosystemsTM Protein Thermal Shift™ Software v1.4 (Thermo Fisher Scientific, Cat# 15681792). Data for ITC experiments was collected using MicroCal PEAQ-ITC analysis software (Malvern).
Data analysis	Cryo-EM data processing and 3D reconstruction were performed using CryoSPARC v4.0.1 (Punjani et al., 2017; RRID:SCR_016501). Further refining was performed in DeepEMhancer (Sanchez-Garcia et al., 2021), Coot v0.9.8.3 (Emsley et al., 2010.; RRID:SCR_014222), ISOLDE (Croll, 2018.; RRID:SCR_025577), and Topaz v0.2.5a (Bepler et al., 2019). Models were generated in software from UCSF ChimeraX 1.6.1 (Pettersen et al., 2004; RRID:SCR_015872), PHENIX v1.20.1-4487 (Adams et al., 2010; RRID:SCR_014224), MolProbity v4.5.1 (Chen et al., 2010; RRID:SCR_014226), Modeller v10.0 (Webb and Sali, 2016; RRID:SCR_008395), Life Science Schrodinger Suite v2023-1 (Schrödinger; RRID:SCR_014879), Desmond Molecular Dynamics System v6.1 (D.E. Shaw Research; RRID:SCR_014575), OPLS4 force field (Lu et al., 2021), Visual Molecular Dynamics (VMD) v1.9.4 (Humphrey et al., 1996; RRID:SCR_001820), and PyMol (Schrödinger; RRID:SCR_000305). Densiometric data from western blots was analyzed with Image Lab (BioRad; RRID:SCR_014210). Statistical analyses and graphs were generated in GraphPad Software, v. 10 (GraphPad Prism Inc.; RRID:SCR_002798).

For manuscripts utilizing custom algorithms or software that are central to the research but not yet described in published literature, software must be made available to editors and reviewers. We strongly encourage code deposition in a community repository (e.g. GitHub). See the Nature Portfolio guidelines for submitting code & software for further information.

Data

Policy information about availability of data

All manuscripts must include a data availability statement. This statement should provide the following information, where applicable:

- Accession codes, unique identifiers, or web links for publicly available datasets
- A description of any restrictions on data availability
- For clinical datasets or third party data, please ensure that the statement adheres to our policy

The authors declare that all data supporting the findings of this study are included in the article, Extended Data, Supplementary Information (including Supplementary Tables), or Supplementary Videos. The coordinates and cryo-EM maps have been deposited in the Protein Data Bank (PDB) and the Electron Microscopy Data Bank (EMDB) under accession codes 9DNM and EMD-47042 for βarr1–Cmpd-5, and 9DNG and EMD-47040 for apo βarr1. Additional data and materials, including those supporting the Supplementary Information, are available upon request.

Research involving human participants, their data, or biological material

Policy information about studies with human participants or human data. See also policy information about sex, gender (identity/presentation), and sexual orientation and race, ethnicity and racism.

Reporting on sex and gender	Data involving sex/gender was not collected.
Reporting on race, ethnicity, or other socially relevant groupings	Data involving race, ethnicity, or social grouping was not collected.
Population characteristics	Data involving human population characteristics was not collected.
Recruitment	No participants were involved in this study.
Ethics oversight	This study did not involve human participants, animal experiments, or the use of identifiable human data or tissue. Therefore, no ethics approval was required.

Note that full information on the approval of the study protocol must also be provided in the manuscript.

Field-specific reporting

Please select the one below that is the best fit for your research. If you are not sure, read the appropriate sections before making your selection.

☒ Life sciences ☐ Behavioural & social sciences ☐ Ecological, evolutionary & environmental sciences

For a reference copy of the document with all sections, see nature.com/documents/nr-reporting-summary-flat.pdf

Life sciences study design

All studies must disclose on these points even when the disclosure is negative.

Sample size	Sample sizes were determined based on standard practices in the field for in vitro pharmacology and biophysical assays. Each key experiment was conducted with a minimum of three biologically independent replicates, unless otherwise specified in the figure legends.
Data exclusions	No data were excluded from analysis. All measurements and replicates were included and are reported in full.
Replication	To verify reproducibility of our findings, experiments were performed with at least three biological replicates. Additional experimental or technical replicates are noted in figure legends where applicable.
Randomization	Not relevant because no human or animal subjects were used in the study.
Blinding	Experimental results were quantitative and did not require subjective analysis. Therefore, blinding was not performed.

Reporting for specific materials, systems and methods

We require information from authors about some types of materials, experimental systems and methods used in many studies. Here, indicate whether each material, system or method listed is relevant to your study. If you are not sure if a list item applies to your research, read the appropriate section before selecting a response.

Materials & experimental systems

n/a	Involved in the study
☐	☒ Antibodies
☐	☒ Eukaryotic cell lines
☒	☐ Palaeontology and archaeology
☒	☐ Animals and other organisms
☒	☐ Clinical data
☒	☐ Dual use research of concern
☒	☐ Plants

Methods

n/a	Involved in the study
☒	☐ ChIP-seq
☐	☒ Flow cytometry
☒	☐ MRI-based neuroimaging

Antibodies

Antibodies used

Rabbit polyclonal anti-phospho-p44/42 MAPK antibody (Cell Signaling, Cat# 9101; RRID:AB_331646).
Rabbit polyclonal anti-MAPK1/2 antibody (Sigma-Aldrich, Cat# ABS44).
Anti-β-arrestin1/2 antibody (A1CT) in rabbit (Lefkowitz Lab, Attramadal et al., 1992).
Monoclonal ANTI-FLAG M2 antibody produced in mouse (Sigma-Aldrich, Cat# A8592; RRID:AB_439702).
Anti-p38 alpha/beta MAPK, mouse monoclonal antibody (Santa Cruz Biotechnology, Cat# sc-7972; RRID:AB_628079).
Anti-phospho-p38 MAPK (Thr180/Tyr182), rabbit antibody (Cell Signaling, Cat# 9211; RRID:AB_331641).
Anti-Rabbit IgG, HRP-linked $F(ab')_2$ fragment from donkey (Cytiva, Cat# NA9340-1ML).
Anti-Mouse IgG, HRP-linked $F(ab')_2$ fragment from sheep (Cytiva , Cat# NA9310-1ML).
Anti-mouse CD3-AF488 (BioLegend, Cat# 10032; RRID:AB_389300).
Anti-mouse CD3-APC (eBioscience, Cat# 17-0031-82).
Anti-mouse CD4-Pacific Blue (BioLegend, Cat# 100427; RRID:AB_493646).
Anti-mouse CD8-APC-Cy7 (BD Biosciences, Cat# 557654; RRID:AB_396769).
Anti-mouse CD45-QD605 (eBioscience, Cat# 93-0451-42).

Validation

Antibodies are commercially available, validated by manufacturers; or when applicable, in literature referenced above.

Eukaryotic cell lines

Policy information about cell lines and Sex and Gender in Research

Cell line source(s)

Human embryonic kidney 293 (HEK293) cells (ATCC, CRL-1573; RRID:CVCL_0045).
Human HEK293 CRISPR/Cas9 βarr1/2-knockout cells (Namkung et al., 2016).
Sf9 (Spodoptera frugiperda) Insect Cells (Expression Systems, Cat# 94-001F; RRID:CVCL_0549).
Human HEK293 cells stably expressing FLAG-tagged β2AR (Shenoy et al., 2006).
Human U2OS cells co-expressing PK-β2V2R and βarr2-EA (DiscoveRx, Ahn et al., 2017).
Human Activated endocytosis U2OS parental line (DiscoveRx, Ahn et al., 2017).
Human HEK293 cells stably expressing ICUE2 (CFP-Epac2-YFP), (Violin et al., 2008).
Human: HEK293 cells stably expressing AT1R, (Hunton et al., 2005).
Mus musculus T lymphocytes isolated from murine wild-type whole spleen, (Rein et al., 2017; Smith et al., 2018)

Authentication

Cell line identities were verified by the original providers or collaborators and maintained under standard culture conditions. Lines were handled separately to avoid cross-contamination.

Mycoplasma contamination

All mammalian cell lines used in this study were routinely tested and confirmed to be free of mycoplasma contamination, including by DNA staining (Hoechst method) and PCR-based assays, performed by the Cell Culture Facility.

Commonly misidentified lines
(See ICLAC register)

No commonly misidentified cell lines were used in this study.

Plants

Seed stocks

Not applicable. No plant material was used in this study.

Novel plant genotypes

Not applicable. The study did not involve any genetically modified or transgenic plants.

Authentication

Not applicable.

Flow Cytometry

Plots

Confirm that:

☒ The axis labels state the marker and fluorochrome used (e.g. CD4-FITC).

☒ The axis scales are clearly visible. Include numbers along axes only for bottom left plot of group (a 'group' is an analysis of identical markers).

☒ All plots are contour plots with outliers or pseudocolor plots.

☒ A numerical value for number of cells or percentage (with statistics) is provided.

Methodology

Sample preparation	Primary splenocytes were prepared from wild-type C57BL/6 mice and used in a single ex vivo T cell migration assay. Cells were pretreated with βarr modulators (Cmpd-5, -46, or -64) for 30min prior to migration toward 100nM CCL19 in a transwell system. Migrated populations were collected and analyzed by flow cytometry.
Instrument	Flow cytometry was performed using a BD LSRFortessa™ analyzer.
Software	Data were analyzed using FlowJo (v10) software.
Cell population abundance	The frequency of migrated T cell subpopulations was quantified relative to vehicle-treated controls. Populations were defined based on CD45+CD3+ (total T cells), CD4+ (helper T cells), and CD8+ (cytotoxic T cells) markers.
Gating strategy	Gating strategy is detailed in Extended Data Fig. 5. Initial gates excluded debris and doublets (FSC/SSC), followed by live/dead exclusion and T cell identification using CD3-FITC and CD45.2-APC. Subsets were further resolved using CD4-BV605 and CD8-PE.

☒ Tick this box to confirm that a figure exemplifying the gating strategy is provided in the Supplementary Information.
