# Supplementary material for: Small-molecule modulation of β-arrestins
Source: Nature. 2026 Jun 24;656(8128):770–9. doi: 10.1038/s41586-026-10683-5 (PMC13489962; doi:10.1038/s41586-026-10683-5)
Supplement: Supplementary file 3 [file 41586_2026_10683_MOESM3_ESM.pdf]

nature portfolio

Peer Review File

Small-molecule modulation of β-Arrestins

Corresponding Author: Professor Robert Lefkowitz

Any redactions in this file are there to maintain patient confidentiality, the confidentiality of unpublished data, or to remove third-party material.

This file contains all reviewer reports in order by version, followed by all author rebuttals in order by version.

Version 1:

Reviewer comments:

Referee #1

(Remarks to the Author)
Kahsai and colleagues describe in detail the discovery and validation of small molecule modulators of beta arrestins. Starting with in vitro thermostability measurements and working up through cell-based assays of arrestin recruitment, signaling and physiological function the authors demonstrate three small molecules with nearly equal partial efficacy. One of these molecules (Cmpd-5) is then further characterized by solving a cryo-EM structure to determine the binding site, which is further validated with site-directed mutagenesis and molecular dynamics simulations. The only significant shortcoming experimentally is inadequate assessment of possible effects on G protein signaling (see below). The large amount of work is beautifully done, the manuscript is clearly written, and the tools described here will certainly find many uses for further preclinical studies. At the same time the study has not uncovered any novel biology, chemistry or technology. For this reason it is my opinion the manuscript would be more appropriate for a more specialized journal with a focus on chemical biology.

Specific comments:

While the blocking effects of these compounds on arrestin recruitment and function are clear the absence of effects on G protein recruitment and signaling is barely addressed; only one experiment looks at total high affinity radioligand binding to the betaAR. There are several simple assays for G protein recruitment and activation that the authors could employ, ideally in arrestin-deficient cells, to demonstrate the absence of effects on the four major families of G proteins.

Are the effects of the 3 compounds studied in detail additive or occlusive? Knowing this might have some practical as well as conceptual value.

Arrestin recruitment assays focused on a few receptors with very robust arrestin recruiting activity, and none of the compounds completely blocked recruitment. Would these blockers completely block arrestin recruitment to receptors that are a bit more average?

30 pM or 120 pM angiotensin II were used for calcium experiments (Figure 2), presumably to produce slow/weak activation of the calcium release machinery, thus allowing time for arrestin-mediated desensitization to occur prior to the response peak. This must have taken some “optimization”. It might be worth mentioning this somewhere, so that readers understand why blocking arrestin makes an initial peak response larger.

The authors might also note that in their recruitment assay these concentrations of angiotensin II do not detectably recruit arrestin (Figure 1i). Presumably the recruitment assay is less sensitive than physiological signaling for some reason?

On lines 322-325 the authors claim that effects on signaling downstream of Gs and Gq are “minimal in Barr1/2 knockout cells”. I believe only Gq signaling was studied in arrestin-deficient cells (Figure 2). It would be great to see experiments on cAMP desensitization in arrestin-deficient cells, especially since some cAMP is now thought to originate from endosomes. In lieu of this the authors should at least correct this paragraph of the discussion.

For the sake of completeness the authors should mention the existence of barbadin (PMID: 28416805), a small molecule that apparently binds to the AP-2 complex and indirectly blocks the functions of beta arrestins that depend on binding to AP-

2.

Referee #2

(Remarks to the Author)
This work describes the discovery and characterization of small-molecule inhibitors of arrestins. I do not need many words to confirm that the G protein–coupled receptor (GPCR) community is in desperate need of such a tool, and this molecule has the potential for broad application in numerous studies in molecular and cellular biology. The topic is of interest to a wide readership, and the significance of the discovery aligns well with the aims of this journal.
Summary: Using differential scanning fluorimetry, authors identify 26 compounds that bind arrestins from a library of 3500 compounds. From this subset, they further select three compounds that reduce arrestin binding to activated b2AR-V2R chimera (here b2V2R). These were characterized in detail. Author demonstrate that the three compounds bind to both arrestins with similar high nm-low um affinities, they do not bind to Galpha s, nor to arr-recruited kinases, they reduce to about 25% arr recruitment to b2V2R, and in a quite similar extent barr2 recruitment to angiotensin II receptor. A more modest reduction of binding is observed at the V2R, where arrestin recruitment in the presence of inhibitors averages about half compared to recruitment in their absence. Functional characterization neatly shows that the compounds increase the extent of Gs and Gq induced second messenger formation by either b2AR or At1R activation, and this effect is related to the presence of arrestin in the system. Further, they substantially reduce internalization of b2V2R and V2R, attenuate arrestin-mediated ERK phosphorylation following b2AR activation. The compounds also significantly affected cell migration in a dose-dependent manner. Authors solve the cryo-EM structure of a barr1-BRIL fusion either in the apo state or bound to compound 5. The position of the small molecule is validated by SAR studies.

The manuscript is well written, structured according to a clear and logical flow (with a small exception, see below), and reads clearly and pleasantly. The figures are good. The experimental design is straightforward, and the data look overall solid. Overall, the authors draw the right conclusions from their findings. I have only a few concerns.

Major:
1) I think authors should better explain the selection process that led to the choice of the three compounds. As the data are presented now, this choice is difficult to follow (detailed comments below).

2) From the comparison of the apo- and Compound 5-bound arrestin structures, as well as previously solved active arrestin conformations, the authors identify structural features that explain impaired binding of arrestin mainly to intracellular loop 2 (ICL2). I would expect this impaired contact to interfere with the formation of a fully engaged complex (core + tail). However, I am not sure what the structural explanation is for why the tail conformation also cannot form, since the binding cleft on the N-terminal domain seems largely unaffected by the inhibitor. This issue should at least be addressed in the discussion.

3) I am not sure about the general applicability of the tool. The authors characterize the molecules using well-established model systems, such as the β2V2R chimera; even the angiotensin and vasopressin receptors are fairly “standard.” What about GPCRs that engage arrestin in fundamentally different ways — for example, GPCRs lacking a C-terminal tail? Do the inhibitors also work in those systems? I miss a broader evaluation of the inhibitor across a wider set of GPCRs, including, for instance, secretin-like GPCRs, NTS1R, or M2R (wt). A bystander BRET assay for arrestin recruitment should be sufficient to address this without too much experimental effort, especially for such an experienced lab.

Minor:
Page 4 line 58-60. I am not sure which secondary DSF assays are meant here, as Extended figure 2 does not contain DSF data. The same sentence is also in the legend of Extended Data Fig. 2. I guess authors refer to panel d of Extended Data Fig. 1?

Beginning of Page 5. Could the authors explain a bit more why these three hits were chosen? What is meant with “tractability”? For instance, why was C74 discarded?

Extended Data Fig. 2: I am wondering why βarr2 recruitment (panel b) does not correlate with the decrease in ligand binding (panel c). I find the statement “Modulator-induced decreases in binding reflect the inhibition of βarr-mediated receptor engagement” difficult to follow. For instance, C18 appears to completely abolish βarr2 binding in panel b, yet it only reduces ligand binding by about 30%, whereas C29 seems to abolish both arrestin and ligand binding (but was not selected for further analysis). Another example, C59 and C60 show comparable effects on arrestin recruitment but have quite different impacts on ligand binding… Do authors have any explanation?

Page 5, lines 84–86: As above, I find the logic difficult to follow. Based on Extended Data Fig. 2, it seems to me that other candidates might have been more promising. I would also suggest that the authors rearrange the information slightly to improve the flow. The section from lines 65–86 on page 5 feels like a jump back to data the reader has already encountered (without explanation) at the bottom of page 4.

Extended Data Fig.3 how many independent experiments?

Page 9, Line 201: I am not sure it is immediately clear to the broader readership what the "hinge region" of arrestin refers to. A brief clarification in the main text would help — for example, something like "which connects the N-terminal and C-terminal domains".

Referee #3

(Remarks to the Author)
This paper represents a significant advancement in GPCR signaling research by reporting the first small-molecule inhibitors that directly target β-arrestins (βarrs), key regulatory proteins in GPCR signaling. The authors employ an impressive multi-faceted approach combining biophysical screening, biochemical/cellular assays, cryo-EM structural analysis, and molecular dynamics simulations to identify and characterize these novel inhibitors. Their discovery of compounds that selectively modulate βarr function opens new possibilities for manipulating GPCR signaling pathways with potential therapeutic applications.

The work is particularly significant because while there are numerous modulators for GPCRs and G proteins, chemical tools to directly target βarrs have been lacking, forcing researchers to rely primarily on genetic approaches. The identification of the MCL pocket as a druggable site on βarrs and the elucidation of the structural mechanism of inhibition provide valuable insights for future drug development targeting the βarr pathway.

Overall, this manuscript presents a significant advance in the field of GPCR signaling by identifying the first small-molecule inhibitors of β-arrestins. Addressing the concerns below, particularly regarding the mechanistic characterization of active/inactive state stabilization, competition with known βarr binders, and physiological relevance to therapeutically important receptor systems, would further strengthen this important work.

Major Questions/Concerns
1. Active vs. Inactive State Stabilization: The authors propose that Cmpd-5 stabilizes an inactive-like conformation of βarr1, but this conclusion needs stronger experimental validation. The cryo-EM structure shows conformational changes compared to both active and inactive states, but direct biophysical measurements comparing how these compounds affect the equilibrium between active and inactive states would strengthen this claim. Additional experiments, such as FRET-based conformational sensors, could monitor βarr activation state in the presence of inhibitors.

2. Prevention of Inactive-to-Active Transition: While the authors suggest that these compounds prevent βarr transition from inactive to active states, this is primarily inferred from structural data rather than demonstrated experimentally. The authors should consider experiments directly measuring the kinetics of βarr activation in the presence of compounds, perhaps using real-time conformational biosensors or hydrogen-deuterium exchange mass spectrometry (HDX-MS) to track conformational changes.

3. Effects on GLP-1R Signaling and Weight Reduction: The paper lacks investigation into the physiological effects of these βarr modulators on clinically relevant GPCR systems like GLP-1R, which is important for metabolic regulation and weight control. Given the therapeutic importance of GLP-1 agonists, demonstrating how these compounds affect GLP-1R signaling, particularly on the balance between G protein and βarr pathways, would significantly enhance the pharmacological relevance of this work.

4. IP6 Competition Studies: The authors should determine whether inositol hexakisphosphate (IP6), a known βarr binder used as a control, competes with Cmpd-5 for binding. Competition binding assays (ITC, DSF, or radiolabeled displacement) would clarify whether these ligands share overlapping binding sites or regulate βarr allosterically. This would provide additional validation of the binding site and insight into potential endogenous regulation.

5. MD Simulation and Binding Energy: While the authors performed MD simulations, more extensive analysis of the low-energy binding states would strengthen the structural conclusions, especially given the moderate resolution of the cryo-EM data and the relatively weak binding affinity. Calculating binding free energies and performing longer simulations with multiple runs would provide more confidence in the proposed binding mode.

6. Binding Mode for Other Compounds: The authors focus structural analysis on Cmpd-5 but do not sufficiently explain how the identified binding site accommodates the structurally distinct Cmpd-46 and Cmpd-64. Docking studies or additional experimental data for these compounds would strengthen the generalizability of the proposed binding mechanism.

7. ITC Validation with Mutated Proteins: While the authors performed some mutagenesis studies to validate the binding site, a more comprehensive set of ITC experiments with strategically selected mutants would provide stronger validation of the binding interactions. Particularly, examining the thermodynamic parameters of binding for key mutations would better characterize the energetic contributions of specific residues.

Minor Questions/Concerns
1. The dose-response curves for βarr inhibition show relatively shallow slopes, suggesting potential complex binding mechanisms or multiple binding sites. This deserves discussion.
2. The authors should clarify whether these compounds show any selectivity between different GPCR systems or whether the inhibition is universal across all βarr-GPCR interactions.
3. Figure 2 data shows substantial enhancement of G protein signaling upon βarr inhibition, but the mechanisms underlying this enhancement (beyond preventing desensitization) should be explored more thoroughly.
4. The authors should clarify whether these compounds affect βarr interactions with non-GPCR partners, since βarrs have

numerous binding partners beyond GPCRs.
5. The manuscript would benefit from more quantitative analysis of the structural changes induced by Cmpd-5, including RMSD calculations between different states.
6. The manuscript occasionally uses "modulator" and "inhibitor" interchangeably, but it should be consistent with terminology throughout.
7. More detailed discussion of the pharmacokinetic properties and potential for in vivo use would strengthen the paper's translational aspects.
8. The observed differences in βarr1 vs. βarr2 selectivity for different compounds deserve more mechanistic explanation based on the structural data.
9. Several typos and language issues need attention:
o Line 128: "CFP-Epac2-YFP)46" missing space before reference
o Line 133: "relative to DMSO control (100%)" - clarify if this is set to 100% or represents raw data
o Line 197: "pβ2V2R" - formatting inconsistency with receptor naming
o Line 387: "divergent mechanisms of βarr modulation" - unclear what divergent mechanisms are being referred to
Lines 208-237: Some redundancy in describing the binding interactions of Cmpd-5

Version 3:

Reviewer comments:

Referee #1

(Remarks to the Author)
For the most part the authors have done an excellent job responding to my suggestions. The one exception is the assessment of off-target inhibition of G protein signaling, specifically the possible inhibition of Gi activity shown in extended data Figure 4d. The approximately 40% decrease is described as “mild reduction in Gi signaling” (line 133), whereas decreases in arrestin recruitment as small as 35% are described as “robust” (line 156). The conclusion that there is “minimal detectable off-target activity” (line 141) is contradicted by the data. The authors provide ITC data that failed to detect compound binding to purified Gαi (notably not a heterotrimer), but this negative result does not clarify what happened in extended data Figure 4d. Given the EC50 for Ach in this figure there is likely a large receptor reserve, therefore the compounds are probably not acting as orthosteric antagonists, but they could certainly be NAMs at M2R or inhibiting Gi heterotrimers directly. The obvious remedy is to assess other Gi-coupled receptors, possibly with more than one assay, to isolate this consistent (across compounds) inhibition to either the receptor or the components of the TRUPATH assay. If it turns out this is a receptor-specific off-target effect, it will be good to know and a cautionary warning to readers that 50 μM concentrations of small molecules can do unexpected things. If it turns out this is a direct effect on Gi heterotrimers it will still not render the compounds useless, but it will be critically important for potential users to know.

Referee #2

(Remarks to the Author)
The authors have substantially improved a manuscript that was already very good. I am satisfied with their responses to my comments and recommend publication of the manuscript in its current form.

Referee #3

(Remarks to the Author)
The authors have provided a thoughtful, comprehensive, and high-quality revision. All major scientific concerns have been fully addressed with new data, and the manuscript now presents a compelling, rigorous, and field-advancing study.

I fully support publication of this manuscript in its current revised form.

It represents a significant contribution to GPCR signaling research and introduces the first well-validated small-molecule probes that directly modulate β-arrestin function—a toolset the field has long needed.

Version 4:

Reviewer comments:

Referee #1

(Remarks to the Author)
The authors have added additional experiments to address my only remaining reservation. A second receptor has been added to the TRUPATH experiment showing that the inhibitory effect of the compounds in this assay are not restricted to the M2R. This is useful information. However, to follow up on this they choose an assay that does not assess Gi activation at all (mini-G recruitment), and a second Gi activation assay that is so odd that it raises questions. The authors assess Rap1GAP recruitment to a tagged beta adrenergic receptor (the manuscript says beta2, the figure says beta1?). The Rap1GAP assay developed by the Bouvier group (ref. 44) showed no significant activation of Gi1 or Gi2 by either beta adrenergic receptor,

making either of these receptors an exceptionally strange choice. Moreover, it is impossible to assess the validity of the data because they are normalized, use a single concentration of agonist, and not validated with beta receptor antagonists or pertussis toxin. The fact that it takes 5 minutes or more for the noisy signals to reach something like a steady state is not consistent with robust Gi activation. Any reader would naturally expect to see data with this assay and the M2R, D2R, or both (or even CCR7), i.e. bona fide Gi-coupled receptors.

Open Access This Peer Review File is licensed under a Creative Commons Attribution 4.0 International License, which permits use, sharing, adaptation, distribution and reproduction in any medium or format, as long as you give appropriate credit to the original author(s) and the source, provide a link to the Creative Commons license, and indicate if changes were made.
In cases where reviewers are anonymous, credit should be given to 'Anonymous Referee' and the source.
The images or other third party material in this Peer Review File are included in the article's Creative Commons license, unless indicated otherwise in a credit line to the material. If material is not included in the article's Creative Commons license and your intended use is not permitted by statutory regulation or exceeds the permitted use, you will need to obtain permission directly from the copyright holder.
To view a copy of this license, visit https://creativecommons.org/licenses/by/4.0/

November 12, 2025

Small-molecule modulation of β-arrestins

Tracking number: Nature-2025-04-08404A

We thank the reviewers for their thoughtful comments and suggestions. Below is our point-by-point response, with changes marked in blue and referenced by line numbers throughout the manuscript. We addressed all concerns through detailed explanations, new experimental data, and revisions to the main text and figure legends. Several new main and extended figures were generated based on reviewer input, covering key structural, biochemical, and functional analyses. Reviewer Figures R1–R2 were included only to clarify one specific comment and are not part of the revised manuscript. We believe these additions and clarifications have substantially strengthened the work.

Referees' comments:

Referee #1 (Remarks to the Author):

Kahsai and colleagues describe in detail the discovery and validation of small molecule modulators of beta arrestins. Starting with in vitro thermostability measurements and working up through cell-based assays of arrestin recruitment, signaling and physiological function the authors demonstrate three small molecules with nearly equal partial efficacy. One of these molecules (Cmpd-5) is then further characterized by solving a cryo-EM structure to determine the binding site, which is further validated with site-directed mutagenesis and molecular dynamics simulations. The only significant shortcoming experimentally is inadequate assessment of possible effects on G protein signaling (see below). The large amount of work is beautifully done, the manuscript is clearly written, and the tools described here will certainly find many uses for further preclinical studies. At the same time the study has not uncovered any novel biology, chemistry or technology. For this reason, it is my opinion the manuscript would be more appropriate for a more specialized journal with a focus on chemical biology.

We thank the reviewer for their thoughtful and constructive evaluation of our study. We appreciate the recognition of the experimental rigor, the progression from in vitro screening to cellular and structural validation, and the potential utility of the modulators in preclinical research. We have carefully addressed all comments, including additional experiments and clarifications, and we believe the revised manuscript is substantially strengthened.

Specific comments:

1. While the blocking effects of these compounds on arrestin recruitment and function are clear the absence of effects on G protein recruitment and signaling is barely addressed; only one experiment looks at total high affinity radioligand binding to the betaAR. There are several simple assays for G protein recruitment and activation that the authors could employ, ideally in arrestin-deficient cells, to demonstrate the absence of effects on the four major families of G proteins.

We appreciate the reviewer's suggestion to more directly assess the impact of the βarr modulators on G protein–related signaling assays. To this end, we performed TRUPATH BRET based biosensor assays in βarr1/2 CRISPR knockout HEK293 cells, as requested, to evaluate GPCR mediated G protein activation across representatives of all four canonical Gα families:

Gαs (β_2AR), Gαq (AT_1R), Gαi (M_2R), and Gα12 (NTSR1). No appreciable effect was observed for Gαs, Gαq, or Gα12, and only a mild reduction in Gαi activation was noted (Extended Data Fig. 4b-e). To investigate whether this reflected direct compound–Gi protein engagement, we first assessed Gαi binding using ITC, a well-established biophysical assay used to quantify direct bimolecular interactions commonly employed in drug discovery. We detected no measurable affinity for Cmpd-5 or Cmpd-46, and only very weak binding for Cmpd-64, a value substantially lower than their binding affinities for βarr1/2, which range from high nanomolar to low micromolar (Extended Data Fig. 4f). These results support the conclusion that the modulators exhibit minimal off target activity on Gαi, and any modest effect observed likely reflects assay specific interference with Gαi rather than true engagement. To further evaluate potential off target binding, we assessed physical interaction of other G proteins with the modulators using DSF, a complementary approach that does not require high recombinant protein concentrations. We found no binding to Gαs or Gαq, or even to known βarr associated kinases including ERK2, c Src, and p38α (Extended Data Fig. 4g,h). Consistent with these findings, our pharmacological radioligand binding studies confirmed that the modulators do not disrupt high affinity Gαsβγ coupling to β_2AR, indicating preserved receptor–G protein interaction and agonist stabilized ternary complex formation (Extended Data Fig. 4a). More convincingly, our Ca^{2+} signaling assay results in wild type and βarr1/2 null cells further support the βarr specificity of these compounds. In HEK293 cells expressing AT_1R, all three βarr modulators enhanced AngII stimulated Ca^{2+}mobilization; however, this effect was abolished in βarr deficient cells, indicating that enhanced signaling depends on βarrs and not altered G protein activity (Fig. 2g–h). Altogether, these results confirm that the βarr modulators selectively engage βarrs while sparing their G protein and βarr binding kinase counterparts, with little to no detectable off target effects across multiple orthogonal assays. These text updates are now included in the revised manuscript (*Main text, page 6, lines 126–141; page 8, lines 189–197; and page 15, lines 415–419*).

2. Are the effects of the 3 compounds studied in detail additive or occlusive? Knowing this might have some practical as well as conceptual value.

We thank the reviewer for this insightful comment. We examined whether the three βarr modulators act additively or occlusively by assessing βarr activity in the context of βarr2 recruitment to agonist-activated β2V2R. As shown in Extended Data Fig. 2d and described in the main text (*page 5, lines 115–129*), combinations of compounds (25 μM + 25 μM) produced inhibition comparable to single treatments at 50 μM. This behavior is functionally consistent with dose additivity without synergy. Functionally, this pattern aligns with their shared inhibitory properties observed across diverse βarr-related responses and with their ability to disrupt βarr engagement with agonist-activated receptors via stabilization of an inactive-like conformation, as observed for Cmpd-5 in complex with βarr1 by cryo-EM. Together, these findings support functionally convergent, non-occlusive mechanisms among the modulators.

3. Arrestin recruitment assays focused on a few receptors with very robust arrestin recruiting activity, and none of the compounds completely blocked recruitment. Would these blockers completely block arrestin recruitment to receptors that are a bit more average?

We thank the reviewer for this insightful comment, which has strengthened the manuscript. We agree that our initial assays focused on strong βarr recruiters (β_2V_2R, V_2R, AT_1R), representative of Class B GPCRs. In response, we broadened our analysis to receptors with

diverse βarr engagement modes, including transient ($NTSR_1$, $CXCR_3$, μOR, β_1AR), βarr-biased (ACKR3), ICL3-coupled receptors lacking a canonical C-terminal tail (M_2R), and high-affinity Class B βarr recruiters (GLP-1R, V_2R, AT_1R, β_2V_2R). In other words, beyond the original three receptors, we expanded our analysis to include βarr1/2 recruitment across 11 total receptor–modulator pairs.

Across this range, the modulators produced strong βarr1/2 recruitment inhibition ranging (~35–94%), with Class B receptors GLP-1R and AT_1R showing the highest effects (~93% and ~82%), comparable to β_2V_2R. Complete blockade was not observed, particularly among Class A-like receptors, which may reflect constitutive, phosphorylation-independent βarr engagement at the receptor core that is less sensitive to the modulators. These findings suggest that the compounds stabilize an inactive-like βarr conformation that primarily inhibits productive coupling rather than abolishing all pre-existing engagement. Although βarr recruitment inhibition did not reach 100%, downstream βarr-dependent processes such as receptor desensitization, internalization, and cell migration were markedly reduced, confirming strong functional inhibition. Together, these findings demonstrate that the modulators act broadly across GPCRs with varying βarr engagement strengths, producing potent and mechanistically insightful inhibition. We have incorporated these new data and interpretations into the revised manuscript (*main text, pages 6–7, lines 143–172; Fig. 1e–g, i, k–m; Extended Data Fig. 5*).

4. 30 pM or 120 pM angiotensin II were used for calcium experiments (Figure 2), presumably to produce slow/weak activation of the calcium release machinery, thus allowing time for arrestin-mediated desensitization to occur prior to the response peak. This must have taken some “optimization”. It might be worth mentioning this somewhere, so that readers understand why blocking arrestin makes an initial peak response larger.

We thank the reviewer for this insightful observation. Indeed, the Ang II concentrations (30 pM for stably and 120 pM for transiently expressed AT_1R) were deliberately optimized to elicit moderate, temporally resolved Ca^{2+} transients, thereby allowing sufficient time for βarr-mediated desensitization to occur before the response peak. This clarification has been added to the Methods section of the *Supplementary Information* (*page 31*) to better explain the experimental design rationale and its relevance to the observed modulation of the calcium signaling peak.

5. The authors might also note that in their recruitment assay these concentrations of angiotensin II do not detectably recruit arrestin (Figure 1i). Presumably the recruitment assay is less sensitive than physiological signaling for some reason?

We thank the reviewer for this helpful comment. The difference in apparent sensitivity between assays is now clarified in the main text (*page 7-8, lines 187-188*), noting that Ca^{2+} signaling is inherently more sensitive than βarr recruitment readouts as in Fig. 1i.

6. On lines 322-325 the authors claim that effects on signaling downstream of Gs and Gq are “minimal in Barr1/2 knockout cells”. I believe only Gq signaling was studied in arrestin-deficient cells (Figure 2). It would be great to see experiments on cAMP desensitization in arrestin-deficient cells, especially since some cAMP is now thought to originate from endosomes. In lieu of this the authors should at least correct this paragraph of the discussion.

We thank the reviewer for catching this oversight in our discussion. Indeed, only Gq-mediated Ca^{2+} signaling was examined in βarr1/2 knockout cells (Fig. 2g,h), whereas the Gs–cAMP signaling was assessed only in wild-type cells. The text has been corrected in the Discussion (*page 15, lines 410–412*) to clarify that the observed effects in βarr1/2 knockout cells refer specifically to Ca^{2+} signaling.

7. For the sake of completeness, the authors should mention the existence of barbadin (PMID: 28416805), a small molecule that apparently binds to the AP-2 complex and indirectly blocks the functions of beta arrestins that depend on binding to AP-2.

We thank the reviewer for this suggestion. We now mention barbadin as an example of an indirect strategy that targets AP-2 to inhibit βarr-mediated endocytosis (page14, lines 395–397).

Referee #2 (Remarks to the Author):
This work describes the discovery and characterization of small-molecule inhibitors of arrestins. I do not need many words to confirm that the G protein–coupled receptor (GPCR) community is in desperate need of such a tool, and this molecule has the potential for broad application in numerous studies in molecular and cellular biology. The topic is of interest to a wide readership, and the significance of the discovery aligns well with the aims of this journal.

Summary: Using differential scanning fluorimetry, authors identify 26 compounds that bind arrestins from a library of 3500 compounds. From this subset, they further select three compounds that reduce arrestin binding to activated b2AR-V2R chimera (here b2V2R). These were characterized in detail. Author demonstrate that the three compounds bind to both arrestins with similar high nm-low um affinities, they do not bind to Galpha s, nor to arr-recruited kinases, they reduce to about 25% arr recruitment to b2V2R, and in a quite similar extent barr2 recruitment to angiotensin II receptor. A more modest reduction of binding is observed at the V2R, where arrestin recruitment in the presence of inhibitors averages about half compared to recruitment in their absence. Functional characterization neatly shows that the compounds increase the extent of Gs and Gq induced second messenger formation by either b2AR or At1R activation, and this effect is related to the presence of arrestin in the system. Further, they substantially reduce internalization of b2V2R and V2R, attenuate arrestin-mediated ERK phosphorylation following b2AR activation. The compounds also significantly affected cell migration in a dose-dependent manner. Authors solve the cryo-EM structure of a barr1-BRIL fusion either in the apo state or bound to compound 5. The position of the small molecule is validated by SAR studies.

The manuscript is well written, structured according to a clear and logical flow (with a small exception, see below), and reads clearly and pleasantly. The figures are good. The experimental design is straightforward, and the data look overall solid. Overall, the authors draw the right conclusions from their findings. I have only a few concerns.

We thank the reviewer for their clear and constructive evaluation of our manuscript. We appreciate the recognition of the need for β-arrestin–targeting small molecules and the potential broad impact of our findings. The reviewer's summary of our structural, biophysical, and pharmacological results aligns well with the goals of this study. We have carefully addressed all comments, including through new experiments. In particular, we expanded the

βarr recruitment assays to a broader range of GPCRs with distinct engagement mechanisms, clarified the compound selection process, and further elaborated the structural interpretation. We believe these revisions refine our conclusions and strengthen the manuscript.

Major:

1. I think authors should better explain the selection process that led to the choice of the three compounds. As the data are presented now, this choice is difficult to follow (detailed comments below).

Thank you for the comment. As noted in our responses to Minor points 4–7, we have clarified how the final three compounds were selected. Briefly, we prioritized modulators with consistent activity, good solubility, and relatively respectable functional properties across assays. While some other hits showed promise, they had features that made them less suitable for detailed follow-up. Please refer to Minor points 4–7 below for additional context.

2. From the comparison of the apo- and Compound 5-bound arrestin structures, as well as previously solved active arrestin conformations, the authors identify structural features that explain impaired binding of arrestin mainly to intracellular loop 2 (ICL2). I would expect this impaired contact to interfere with the formation of a fully engaged complex (core + tail). However, I am not sure what the structural explanation is for why the tail conformation also cannot form, since the binding cleft on the N-terminal domain seems largely unaffected by the inhibitor. This issue should at least be addressed in the discussion.

We appreciate the reviewer's point and have addressed this in the revised manuscript. Although the binding cleft on the N-terminal domain is largely unaffected by Cmpd-5, the compound stabilizes a distinct βarr conformation that prevents its transition to the fully active state. Consequently, while the tail-binding site remains structurally intact, Cmpd-5 locks the relative twist motion between the βarr domains, thereby rendering formation of the βarr–receptor tail complex incompatible with the Cmpd-5–bound conformation. To further explore potential competition between IP_6 (a known active-state stabilizing ligand) and Cmpd-5, we performed DSF assays and structural comparisons. These showed that IP_6 and Cmpd-5 stabilize distinct conformational states of βarr1/2, and that Cmpd-5 shifts βarr away from the IP_6-induced active state when both ligands are present. Structural overlays confirmed that the two ligands engage distinct, non-overlapping pockets: IP_6 binds the canonical phosphate groove across the N and C domains, with the N-domain groove known to interact with phosphorylated GPCR tails, whereas Cmpd-5 occupies the MCL cleft formed by the middle, C, and lariat loops. Although these sites are spatially separate, Cmpd-5 allosterically locks βarr in a conformation that is incompatible with both core and tail engagement, thereby preventing formation of the fully engaged complex. These results have been incorporated into the revised manuscript (Main text, pages 13-14, lines 352–390; page 16-17, lines 453–482; Extended Data Fig. 12; Fig.5e).

3. I am not sure about the general applicability of the tool. The authors characterize the molecules using well-established model systems, such as the β2V2R chimera; even the angiotensin and vasopressin receptors are fairly "standard." What about GPCRs that engage arrestin in fundamentally different ways — for example, GPCRs lacking a C-terminal tail? Do the inhibitors also work in those systems? I miss a broader evaluation of the inhibitor across a wider set of GPCRs, including, for instance, secretin-like GPCRs, NTS1R, or M_2R (wt). A

bystander BRET assay for arrestin recruitment should be sufficient to address this without too much experimental effort, especially for such an experienced lab.

We appreciate this thoughtful and important comment and fully agree on the importance of assessing the generalizability of βarr modulators across GPCRs with diverse coupling mechanisms. Accordingly, we expanded our analysis beyond the receptors included in the original study (AT_1R, V_2R, and the chimeric β_2V_2R) to encompass receptors representing distinct βarr engagement modes, including GLP-1R (Class B1; tail-mediated βarr recruitment and of notable metabolic and therapeutic relevance), NTS1R (Class A-like; transient βarr engagement), M_2R (ICL3-mediated; lacking a canonical tail), $ACKR_3$ (βarr-biased, G-protein–independent), β_1AR, μOR, and $CXCR_3$. Together, these ten receptors with divergent properties added to the original three provide a comprehensive framework covering constitutive, canonical C-tail–, and ICL3/core phosphorylation–driven mechanisms across GPCR classes. βarr recruitment was quantified using a rapid NanoBiT complementation assay in βarr1/2-null HEK293 cells. Across this expanded receptor panel, all three modulators consistently and strongly attenuated βarr engagement, with potency differences reflecting receptor-specific βarr coupling profiles. Collectively, these findings demonstrate the broad applicability of the modulators across distinct βarr engagement mechanisms and highlight their value as chemical probes to dissect β-arrestin-dependent signaling. This expanded analysis is now included in the revised manuscript (*main text, pages 6–7, lines 143–171; Fig. 1e–g, i, k–m; Extended Data Fig. 5*).

Minor:

1. Page 4 line 58-60. I am not sure which secondary DSF assays are meant here, as Extended figure 2 does not contain DSF data. The same sentence is also in the legend of Extended Data Fig. 2. I guess authors refer to panel d of Extended Data Fig. 1?

We agree that the term "secondary" may be unclear in this context, and we have removed it now and clarified the surrounding text to improve clarity, referring to Extended Data Fig. 1d, which we were referencing, as correctly inferred by the reviewer.

2. Beginning of Page 5. Could the authors explain a bit more why these three hits were chosen? What is meant with "tractability"? For instance, why was C74 discarded?

The final selection of the three prioritized compounds (Cmpd-5, Cmpd-46, and Cmpd-64) was based on multiple factors, including consistent inhibition of βarr activity across diverse orthogonal assays and favorable chemical properties such as solubility, stability, and minimal assay interference. Several other compounds, including C74, showed promising activity. For example, C74 robustly inhibited βarr2 recruitment (Reviewer Fig. R1a) and ERK signaling. However, it was not selected for detailed follow-up due to specific chemical and physicochemical considerations. C74 exhibited a yellowish color and visible turbidity at working concentrations (Reviewer Fig. R1b), raising the possibility of assay interference. In addition, it contains a nitro group, a functional moiety sometimes flagged in early drug discovery due to concerns about potential toxicity or metabolic instability. While C74 remains a valuable hit, we chose to focus on a tractable subset of modulators better suited for detailed mechanistic and structural analysis. This clarification has been incorporated into the revised manuscript (Main text, pages 4–5, lines 87–115; see also Extended Data Fig. 2 legend).

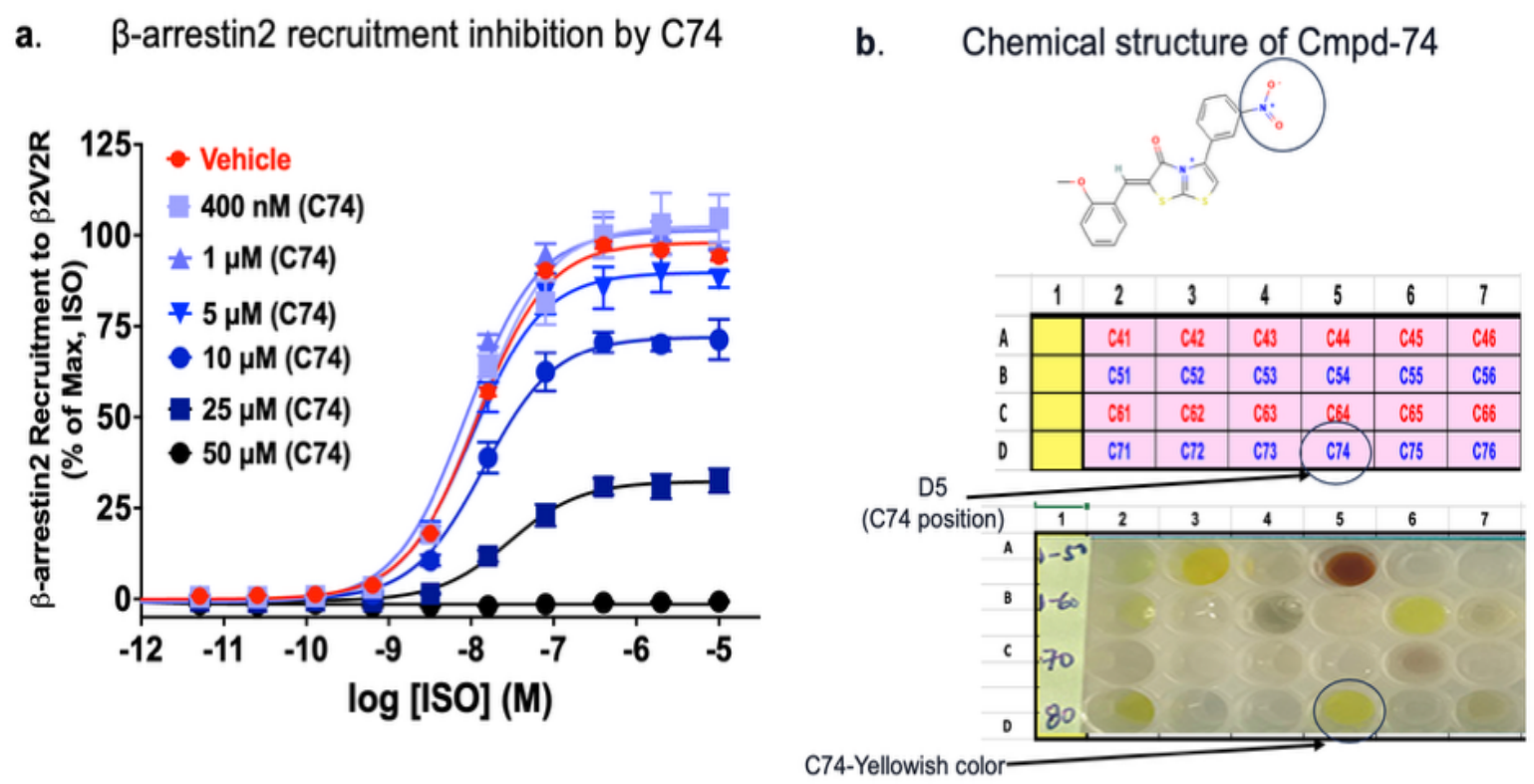

Reviewer Fig. R1 | Inhibition profile and structural features of Cmpd-74. **a**, Dose-dependent inhibition of βarr2 recruitment to β_2V_2R by Cmpd-74, measured in U2OS-PathHunter cells. Cells were treated with increasing concentrations of C74 prior to isoproterenol (ISO) stimulation. Recruitment was quantified as % of maximal ISO response (red curve). **b**, Chemical structure of C74 (top), 96-well layout showing compound plate position (D5), and plate image highlighting its yellowish appearance and turbidity at working concentrations. C74 contains a nitro group (circled), a chemical feature that can raise early-stage development considerations.

3. Extended Data Fig. 2: I am wondering why βarr2 recruitment (panel b) does not correlate with the decrease in ligand binding (panel c). I find the statement "Modulator-induced decreases in binding reflect the inhibition of βarr-mediated receptor engagement" difficult to follow. For instance, C18 appears to completely abolish βarr2 binding in panel b, yet it only reduces ligand binding by about 30%, whereas C29 seems to abolish both arrestin and ligand binding (but was not selected for further analysis). Another example, C59 and C60 show comparable effects on arrestin recruitment but have quite different impacts on ligand binding. Do author have any explanation?

As noted, Extended Data Fig. 2b and 2c examine related but mechanistically distinct aspects of βarr function. Panel 2c measures βarr2 recruitment to agonist-activated β_2V_2R in live U2OS cells using the PathHunter assay, a cellular context influenced by compound permeability, intracellular stability, distribution, and even potential off-target effects. In contrast, panel 2b evaluates βarr1/2-facilitated high-affinity agonist binding to phosphorylated β_2V_2R ($p\beta_2V_2R$) in a defined, cell-free system, providing a direct readout of βarr–receptor engagement. Given these differences, strict correlation between assays is not expected. For example, C18 strongly inhibits βarr2 recruitment at 10 nM ISO (~EC_{50}) but shows only partial inhibition in the binding assay, likely due to isoform selectivity or compound-specific limitations such as physicochemical constraints. Similarly, C29 shows dual activity but was not pursued further due to limited solubility. To illustrate this further with an example, we now include previously generated dose response curves (Reviewer Fig. R2) comparing βarr2 recruitment and receptor internalization. These data confirm that while C18 and C29 were active at a single agonist dose (10 nM ISO, ~EC_{50}), their inhibition across a full range was modest. In contrast, Cmpd-5 showed robust and consistent activity across orthogonal assays, supporting its advancement for detailed characterization. We also appreciate the reviewer's note on the figure legend text. We have clarified that the red dashed reference line in panel 2b now reflects βarr-promoted radioligand-agonist binding, and that reductions below this line indicate inhibition of βarr-mediated receptor engagement. Lastly, while we agree hits such as C18, C29, C59, and C60 remain of interest, our focus was to prioritize modulators with consistent activity, profiles, favorable chemical properties, and suitability for mechanistic and structural analysis. We have revised the manuscript to reflect these distinctions as also indicated in other comments above (Main text, pages 4–5, lines 87–115).

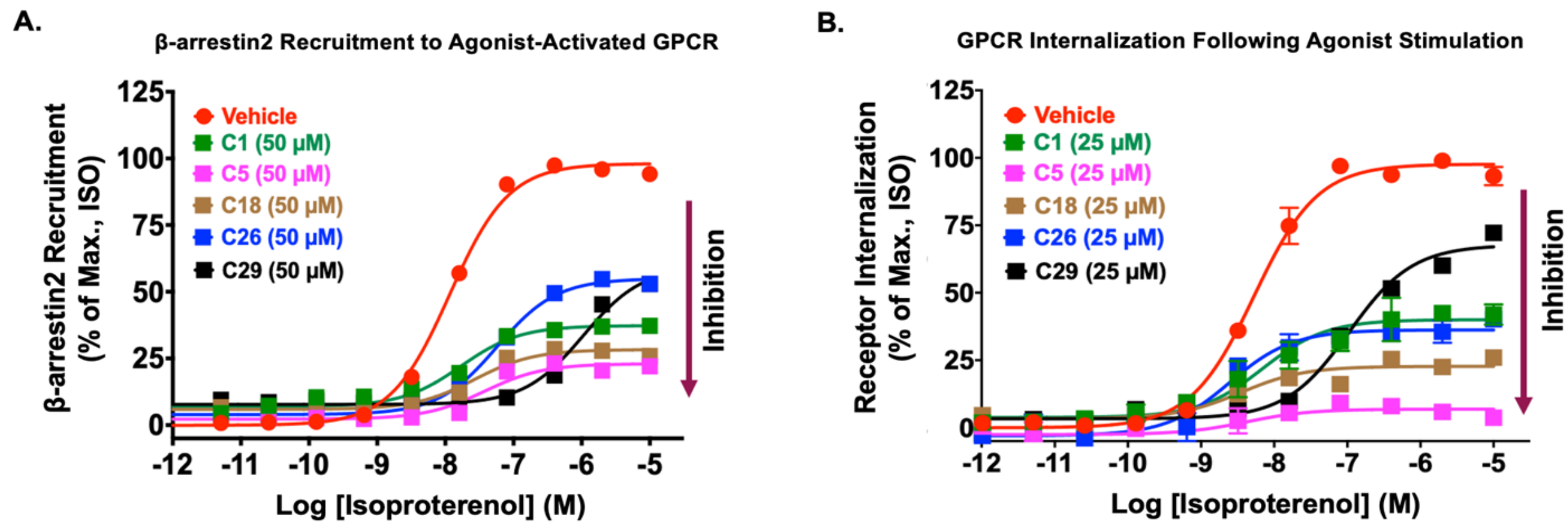

Reviewer Fig. R2 | Differential dose–response effects of βarr modulators on βarr2 recruitment and receptor internalization. **a**, βarr2 recruitment assessed in U2OS-PathHunter cells co-expressing β_2V_2R and βarr2. Cells were pretreated with putative βarr modulators (50 µM, 30 min) and stimulated with increasing doses of isoproterenol (ISO). Data represent % recruitment relative to maximum ISO response (red curve). **b**, GPCR internalization measured in U2OS-PathHunter cells expressing untagged β_2V_2R, EA-tagged βarr2, and a PK-endosomal marker. Modulators (25 µM) were applied prior to ISO stimulation. Internalization was quantified via β-gal complementation. Arrows highlight the degree of inhibition relative to the ISO-only control. Note: Data shown for visualization; n = 1–2 (not included in main or extended figures).

4. Page 5, lines 84–86: As above, I find the logic difficult to follow. Based on Extended Data Fig. 2, it seems to me that other candidates might have been more promising. I would also suggest that the authors rearrange the information slightly to improve the flow. The section from lines 65–86 on page 5 feels like a jump back to data the reader has already encountered (without explanation) at the bottom of page 4.

We thank the reviewer for the helpful suggestion. In response, we have revised the text to clarify the rationale and improve the flow of compound selection, ensuring that the logic now follows directly from the screening data in Extended Data Figs. 1–2 and Fig. 1. As previously noted (see our response to Comment 3 above), final compound prioritization reflects not only functional performance but also chemical properties and overall tractability, which are now described with improved continuity in the revised manuscript (Main text, pages 4–5, lines 83–115).

5. Extended Data Fig.3 how many independent experiments?

The ITC experiments were independently performed 2–3 times for each modulator–βarr pair, with representative curves shown in the figure, which has now been clarified in the legend.

6. Page 9, Line 201: I am not sure it is immediately clear to the broader readership what the "hinge region" of arrestin refers to. A brief clarification in the main text would help — for example, something like "which connects the N-terminal and C-terminal domains".

We have clarified in the main text that the hinge region refers to the flexible linker connecting the N- and C-terminal domains of βarr (pages 10, lines 268-269 and 278-279).

Referee #3 (Remarks to the Author):

This paper represents a significant advancement in GPCR signaling research by reporting the first small-molecule inhibitors that directly target β-arrestins (βarrs), key regulatory proteins in GPCR signaling. The authors employ an impressive multi-faceted approach combining biophysical screening, biochemical/cellular assays, cryo-EM structural analysis, and molecular dynamics simulations to identify and characterize these novel inhibitors. Their discovery of compounds that selectively modulate βarr function opens new possibilities for manipulating GPCR signaling pathways with potential therapeutic applications. The work is particularly significant because while there are numerous modulators for GPCRs and G proteins, chemical tools to directly target βarrs have been lacking, forcing researchers to rely primarily on genetic approaches. The identification of the MCL pocket as a druggable site on βarrs and the elucidation of the structural mechanism of inhibition provide valuable insights for future drug development targeting the βarr pathway. Overall, this manuscript presents a significant advance in the field of GPCR signaling by identifying the first small-molecule inhibitors of β-arrestins. Addressing the concerns below, particularly regarding the mechanistic characterization of active/inactive state stabilization, competition with known βarr binders, and physiological relevance to therapeutically important receptor systems, would further strengthen this important work.

We thank the reviewer for their clear and constructive evaluation of our manuscript. We appreciate the recognition of this work as a significant advance in GPCR signaling and the acknowledgement of both its mechanistic depth and the broader utility of direct β-arrestin modulators. The reviewer's comments on the multidisciplinary strategy, including structural, biophysical, and pharmacological analyses, align with the goals of this study. We have carefully addressed all comments. Several points prompted clarification and, in select cases, new experiments, particularly relating to mechanistic interpretation and the physiological effects of the compounds. We believe these additions further refine our conclusions and strengthen the manuscript.

Major
Questions/Concerns
1. Active vs. Inactive State Stabilization: The authors propose that Cmpd-5 stabilizes an inactive-like conformation of βarr1, but this conclusion needs stronger experimental validation. The cryo-EM structure shows conformational changes compared to both active and inactive states, but direct biophysical measurements comparing how these compounds affect the equilibrium between active and inactive states would strengthen this claim. Additional experiments, such as FRET-based conformational sensors, could monitor βarr activation state in the presence of inhibitors.

In addition to our cryo-EM structures (apo- and Cmpd-5–bound βarr1) and MD simulations, comparative analysis with the active V_2RRpp-bound βarr1 structure (PDB: 4JQI) shows that Cmpd-5 stabilizes an inactive-like conformation of βarr, which is incompatible with receptor engagement (Main Fig. 5; Extended Data Fig. 12). To directly support this conclusion, we now provide two complementary functional readouts. First, DSF analysis shows that Cmpd-5 prevents stabilization of βarr1/2 by IP_6, shifting the equilibrium in favor of the inactive-like, Cmpd-5–stabilized state (Extended Data Fig. 12f). Second, live-cell FlAsH–BRET conformational sensors reveal position-specific βarr2 signatures: Cmpd-5 induces a pattern

that resembles the basal (inactive) state and clearly diverges from the active conformation triggered by AngII at AT_1R (Fig. 5e). Together with our structural and simulation data, these findings demonstrate that Cmpd-5 stabilizes an inactive-like βarr conformation that functionally opposes activation. All supporting data are now included in as indicated and in Main text as in (pages 13–14, lines 352–390; pages 16–17, lines 453–482).

2. Prevention of Inactive-to-Active Transition: While the authors suggest that these compounds prevent βarr transition from inactive to active states, this is primarily inferred from structural data rather than demonstrated experimentally. The authors should consider experiments directly measuring the kinetics of βarr activation in the presence of compounds, perhaps using real-time conformational biosensors or hydrogen-deuterium exchange mass spectrometry (HDX-MS) to track conformational changes.

This point is now addressed through additional experiments that directly probe βarr conformational dynamics in addition to our MD simulations. To determine whether Cmpd-5 impedes the shift toward an active state, we employed two independent, functional approaches. First, DSF assays reveal that Cmpd-5 inhibits IP_6-induced stabilization of βarr1/2 *(where IP_6 is known to promote active-like conformations of βarr1/2)*, effectively biasing the equilibrium toward a Cmpd-5–bound inactive-like conformation (*Extended Data Fig. 12f*). Second, FlAsH–BRET βarr2 sensors demonstrate that Cmpd-5 induces position-specific conformational changes resembling the basal (inactive) state, and clearly distinct from the active signature evoked by AngII stimulation of AT_1R (*Fig. 5e*). Combined with our structural and simulation data, these results demonstrate that Cmpd-5 functionally restricts βarr activation by stabilizing an inactive-like conformation. These updates are included in the revised manuscript (pages 13-14, lines 352–390; page 16-17, lines 453–482; Extended Data Fig. 12; Fig.5e).

3. Effects on GLP-1R Signaling and Weight Reduction: The paper lacks investigation into the physiological effects of these βarr modulators on clinically relevant GPCR systems like GLP-1R, which is important for metabolic regulation and weight control. Given the therapeutic importance of GLP-1 agonists, demonstrating how these compounds affect GLP-1R signaling, particularly on the balance between G protein and βarr pathways, would significantly enhance the pharmacological relevance of this work.

We appreciate the reviewer's comment regarding the importance of assessing βarr modulators in the context of the therapeutic significance of GLP-1R signaling in metabolic regulation and weight control. While we did not evaluate systemic metabolic phenotypes, we directly tested the effect of our modulators on βarr1 recruitment to GLP-1R using semaglutide, a clinically used GLP-1R agonist. As shown in Fig. 1k (Main text, pages 6–7, lines 143–173), all three compounds (Cmpd-5, -46, and -64) strongly inhibited βarr1 recruitment, with an average inhibition of ~93 percent—one of the most pronounced effects across the GPCR panel (Fig. 1e-g,i,k-m; Extended Data Fig. 5). These data highlight the broad applicability of our compounds and support their use in dissecting βarr function at clinically relevant receptors. From a translational perspective, inhibition of βarr recruitment at GLP-1R may influence signal bias and receptor lifetime, especially given reports that sustained G protein Gs-mediated cAMP signaling supports prolonged insulin secretion. In this context, βarr inhibition could reduce

receptor desensitization and internalization, potentially sustaining GLP-1R G protein pathway signaling (Discussion, page 15, lines 421–429).

To expand our interrogation of βarr-dependent physiology, we examined another well-characterized βarr-mediated process: cardiomyocyte contractility downstream of AT_1R. As shown in Fig. 3e,f and Extended Data Fig. 7e (Results, pages 9-10, lines 247–259; Discussion, page 16, lines 439–445), stimulation with the βarr-biased agonist TRV027 enhanced sarcomere shortening and contraction kinetics in isolated adult mouse cardiomyocytes, consistent with known βarr-driven inotropic responses. These effects were robustly suppressed by all three modulators, extending the physiological relevance of βarr inhibition beyond immune cell migration.

4. G Competition Studies: The authors should determine whether inositol hexakisphosphate (IP6), a known βarr binder used as a control, competes with Cmpd-5 for binding. Competition binding assays (ITC, DSF, or radiolabeled displacement) would clarify whether these ligands share overlapping binding sites or regulate βarr allosterically. This would provide additional validation of the binding site and insight into potential endogenous regulation.

To evaluate potential competition between IP_6 and Cmpd-5, we performed DSF-based conformational assays and structural comparisons. These analyses showed that the two ligands stabilize distinct conformational states of βarr1/2, and that Cmpd-5 shifts βarr away from the IP_6-induced active state when both ligands are present. IP_6 is known to stabilize the active conformation of βarr1/2 by engaging the canonical phosphate groove that spans the N- and C-domains, including the N-domain surface that also binds phosphorylated GPCR tails. In contrast, Cmpd-5 binds within the MCL cleft, a regulatory site formed by the middle, C, and lariat loops, located in the central crest of βarr.

Although these pockets are spatially distinct and non-overlapping, structural overlays and functional assays suggest a clear allosteric relationship: Cmpd-5 promotes a βarr conformation that is structurally incompatible with the IP_6-stabilized state. These findings support an allosteric mechanism by which Cmpd-5 functionally opposes βarr activation, preventing formation of the fully engaged receptor complex (core + tail). These results have been incorporated into the revised manuscript (Main text, page 14, lines 376–390; pages 16–17, lines 453–482; Extended Data Fig. 12a, e, f).

5. MD Simulation and Binding Energy: While the authors performed MD simulations, more extensive analysis of the low-energy binding states would strengthen the structural conclusions, especially given the moderate resolution of the cryo-EM data and the relatively weak binding affinity. Calculating binding free energies and performing longer simulations with multiple runs would provide more confidence in the proposed binding mode.

We appreciate the reviewer's suggestion and have taken additional steps to strengthen the computational support for our proposed binding model. Our original submission already included extended simulations, as detailed in Extended Data Table 2, with several trajectories reaching the microsecond scale. Furthermore, Supplementary Videos 1 and 2 illustrate stable engagement of Cmpd-5 within the binding pocket during a representative 3-μs trajectory. To further address this point, we have now extended the Cmpd-5–bound simulations to a

cumulative length of ~10 μs, which confirm that the ligand remains largely engaged within the MCL cleft over long timescales—consistent with its moderate experimental affinity. In addition, we performed absolute binding free energy perturbation (AB-FEP) calculations for Cmpd-5 (see Methods, page 41; Results, page 12, lines 311–319). The calculated binding free energy (~4 kcal/mol) corresponds nominally to a micromolar-to-millimolar affinity range. While AB-FEP methods typically exhibit systematic errors of 1–2 kcal/mol (or more), this computed affinity aligns with our experimentally determined binding affinity values, which range from ~400 nM (ITC) to 2–10 μM (functional assays). We note that while AB-FEP is most powerful for comparing relative affinities across multiple ligands; nonetheless, even for this single compound, the order-of-magnitude agreement provides meaningful validation of the binding interaction. Together, these extended simulations and free energy calculations provide additional support for the proposed binding mode, as presented in the manuscript (Fig. 4e; Extended Data Fig. 10d; Extended Data Table 2; Extended Data Fig. 12g; Supplementary Videos 1 and 2).

6. Binding Mode for Other Compounds: The authors focus structural analysis on Cmpd-5 but do not sufficiently explain how the identified binding site accommodates the structurally distinct Cmpd-46 and Cmpd-64. Docking studies or additional experimental data for these compounds would strengthen the generalizability of the proposed binding mechanism.

In response to the reviewer's suggestion, we performed docking studies to assess whether the MCL site identified for Cmpd-5 can also accommodate the structurally distinct βarr modulators Cmpd-46 and Cmpd-64. While docking of Cmpd-5 at the observed site closely reproduced its cryo-EM pose, with strong alignment of key functional groups and favorable interactions—Cmpd-46 and Cmpd-64 yielded disfavored poses, with poor shape and contact complementarity. These findings suggest that the site preferentially accommodates Cmpd-5, whereas the other chemotypes may engage βarrs via distinct binding modes. Relevant data have been incorporated into the revised manuscript (page 12, lines 319–322; page 17, lines 487–493; Extended Data Fig. 10e, f).

7. ITC Validation with Mutated Proteins: While the authors performed some mutagenesis studies to validate the binding site, a more comprehensive set of ITC experiments with strategically selected mutants would provide stronger validation of the binding interactions. Particularly, examining the thermodynamic parameters of binding for key mutations would better characterize the energetic contributions of specific residues.

We appreciate this insightful suggestion and agree that thermodynamic characterization of specific βarr1 mutants enhances understanding of the binding interface. Building on our structural data, we performed targeted alanine substitutions at residues identified by cryo-EM and MD simulations as contributing to Cmpd-5 binding. To evaluate their impact, we combined functional coupling assays with ITC-based analysis. A subset of mutations, including L129, P133, E134, K138, Y249, C251, and R285, was selected to probe the contributions of both polar and hydrophobic contacts within the pocket. ITC measurements revealed that several of these substitutions significantly reduced Cmpd-5 binding affinity and altered enthalpic values. Notably, E134A and R285A markedly impaired both affinity and enthalpy, consistent with disruption of key stabilizing interactions observed in the cryo-EM structure. Additional mutations such as Y249A and C251A also weakened binding, as reflected in both affinity and thermodynamic profiles, highlighting their role in Cmpd-5 engagement. Together, these functional and thermodynamic results provide energetic validation of the binding interface and

support the structural model derived from our cryo-EM analysis. These findings are presented in Fig. 4g and Extended Data Fig. 11c–j, and are described in the revised manuscript (pages 12–13, lines 332–347).

Minor Questions/Concerns

1. The dose-response curves for βarr inhibition show relatively shallow slopes, suggesting potential complex binding mechanisms or multiple binding sites. This deserves discussion.

We appreciate the reviewer's observation. The dose-response curves for βarr inhibition show consistent and concentration-dependent suppression of βarr recruitment across a broad range of modulator doses. While minor deviations from ideal slope behavior can arise due to assay-specific factors—such as transient transfection variability or signal saturation—we believe the observed responses are pharmacologically consistent with single-site inhibition. We have now briefly noted this point in the revised manuscript, as appropriate. We agree that additional kinetic or mechanistic studies could further clarify the modulators' behavior in future work.

2. The authors should clarify whether these compounds show any selectivity between different GPCR systems or whether the inhibition is universal across all βarr-GPCR interactions.

The question of whether the βarr modulators act selectively or broadly across GPCRs is well taken. To address this, we tested the compounds across receptors with diverse βarr engagement profiles. Altogether, we assessed βarr1/2 recruitment across nearly a dozen receptor–modulator combinations, spanning GPCRs with transient, canonical C-tail–, and ICL3/core-driven βarr coupling. All three modulators consistently inhibited βarr recruitment, with variable efficacy reflecting receptor-specific βarr coupling features. This indicates that the compounds broadly impair productive βarr coupling rather than universally blocking all interactions. These findings support their utility as chemical tools to probe βarr-mediated signaling across distinct GPCR systems (*main text, pages 6–7, lines 143–171; Fig. 1e–g, i, k–m; Extended Data Fig. 5*).

3. Figure 2 data shows substantial enhancement of G protein signaling upon βarr inhibition, but the mechanisms underlying this enhancement (beyond preventing desensitization) should be explored more thoroughly.

We appreciate the reviewer's insightful comment. In this study, the enhancement of G protein signaling is interpreted primarily as a result of impaired βarr-mediated desensitization. However, we agree that additional βarr-dependent mechanisms, including receptor internalization, trafficking, spatial signaling from endosomes, and other processes related to the regulatory and scaffolding functions of βarrs, may also contribute. Exploring how these pathways affect sustained G protein signaling following βarr inhibition represents an interesting direction for future investigation.

4. The authors should clarify whether these compounds affect βarr interactions with non-GPCR partners, since βarrs have numerous binding partners beyond GPCRs.

In light of the reviewer's comment, we examined βarr1 interactions with several prominent non-GPCR effector proteins: Src, ERK2, and JNK3, in the presence and absence of each modulator using a FLAG pulldown assay. As shown in Extended Data Fig. 7b, all compounds qualitatively reduced βarr1 binding. Cmpd-5 significantly inhibited interactions with ERK2, while Cmpd-46

and Cmpd-64 markedly reduced Src binding. In contrast, JNK3 binding remained largely unchanged. These findings suggest that the modulators differentially influence βarr–effector associations and may selectively impair downstream signaling. This analysis is now included in the revised manuscript (Extended Data Fig. 7b; page 9, lines 231-233).

5. The manuscript would benefit from more quantitative analysis of the structural changes induced by Cmpd-5, including RMSD calculations between different states.

We now provide quantitative comparisons between the apo, Cmpd-5–bound, and active βarr1 cryo-EM structures, including Cα RMSD values and Q-scores, in the revised manuscript (Main text, pages 13–14, lines 354–355 and 371–373; Fig. 5a,d; Extended Data Fig. 12a), as well as in the corresponding figure legends.

6. The manuscript occasionally uses "modulator" and "inhibitor" interchangeably, but it should be consistent with terminology throughout.

We appreciate the reviewer's comment. As this represents the first report of small-molecule modulators directly targeting β-arrestins, we use the term "modulators" broadly to reflect their ability to regulate βarr function. When referring to specific functional inhibition, we used the term "inhibitors." We have reviewed the manuscript to apply this distinction as consistently as possible and hope the terminology offers clarity while capturing both the specificity and broader implications of our findings.

7. More detailed discussion of the pharmacokinetic properties and potential for in vivo use would strengthen the paper's translational aspects.

We appreciate the reviewer's interest in the translational potential of these modulators. While this study primarily focuses on in vitro characterization and mechanistic insights, the identification of small-molecule modulators that selectively target βarr function represents a long-standing goal in the field, enabling direct chemical control of βarrs in both cellular and physiological contexts. Their robust effects on T cell migration and cardiomyocyte contractility underscore their immediate utility in probing βarr-regulated processes ex vivo, as well as their potential applicability in vivo. These compounds also display encouraging drug-like properties, including favorable molecular size, natural product-like scaffolds, and aqueous solubility with acceptable CLogP values, supporting their viability as chemical starting points, even prior to formal pharmacokinetic profiling. We have initiated studies to evaluate plasma and tissue stability to guide systemic delivery and future optimization. These points have been incorporated into the revised manuscript (Main text; page 16, lines 445-451).

8. The observed differences in βarr1 vs. βarr2 selectivity for different compounds deserve more mechanistic explanation based on the structural data.

While our study aimed to develop general βarr modulators, we observed modest isoform-specific differences in binding affinity and functional responses. For example, Cmpd-64 exhibited approximately 6-fold selectivity for βarr2 over βarr1. Structural analysis of the Cmpd-5–βarr1 complex revealed that key residues within the MCL cleft are conserved across isoforms, supporting a broadly similar binding mode for Cmpd-5 in βarr2.

However, subtle differences in conformational dynamics and pocket flexibility between βarr1 and βarr2 may contribute to the observed variations in binding and function. Such effects are well documented among homologous proteins, where conserved binding pockets can still yield distinct ligand responses due to variations in structural plasticity or distal allosteric coupling. Despite ~78% sequence identity, these factors likely underlie the isoform-specific differences observed in our assays.

We have clarified this point in the revised manuscript (Main text, page 13, lines 347–350; page 17, lines 484–487; Fig. 4h) and highlight it as a promising area for future exploration. A more focused effort on isoform-selective modulators and detailed mechanistic studies will be important to fully define βarr isoform divergence.

9. Several typos and language issues need attention:
o Line 128: "CFP-Epac2-YFP)46" missing space before reference
o Line 133: "relative to DMSO control (100%)" - clarify if this is set to 100% or represents raw data
o Line 197: "pβ2V2R" - formatting inconsistency with receptor naming
o Line 387: "divergent mechanisms of βarr modulation" - unclear what divergent mechanisms are being referred to
o Lines 208-237: Some redundancy in describing the binding interactions of Cmpd-5

We have reviewed and revised the manuscript for clarity, formatting consistency (including receptor naming), and to address any potential redundancy in the indicated sections. Quantitative language has been clarified to indicate normalization relative to DMSO, set as 100% (page 7, line 183). The phrase "divergent mechanisms of βarr modulation" has also been revised for precision. We thank the reviewer for highlighting these formatting-related points.

February 15, 2026

Small-molecule modulation of β-arrestins

Tracking number: Nature-2025-04-08404C-Z

We thank the reviewers for their thoughtful and constructive comments on our study and for their positive recommendation for publication. We also appreciate the additional point raised during review, which we address below in the revised manuscript. This point has been addressed through additional experiments and corresponding revisions to the main text and Extended Data figure legends. Our responses are shown in blue. We believe these additions further strengthen the manuscript.

Referees' comments:

Referee #1 (Remarks to the Author):

For the most part the authors have done an excellent job responding to my suggestions. The one exception is the assessment of off-target inhibition of G protein signaling, specifically the possible inhibition of Gi activity shown in extended data Figure 4d. The approximately 40% decrease is described as "mild reduction in Gi signaling" (line 133), whereas decreases in arrestin recruitment as small as 35% are described as "robust" (line 156). The conclusion that there is "minimal detectable off-target activity" (line 141) is contradicted by the data. The authors provide ITC data that failed to detect compound binding to purified Gαi (notably not a heterotrimer), but this negative result does not clarify what happened in extended data Figure 4d. Given the EC50 for Ach in this figure there is likely a large receptor reserve, therefore the compounds are probably not acting as orthosteric antagonists, but they could certainly be NAMs at M2R or inhibiting Gi heterotrimers directly. The obvious remedy is to assess other Gi-coupled receptors, possibly with more than one assay, to isolate this consistent (across compounds) inhibition to either the receptor or the components of the trupath assay. If it turns out this is a receptor-specific off-target effect, it will be good to know and a cautionary warning to readers that 50 μM concentrations of small molecules can do unexpected things. If it turns out this is a direct effect on Gi heterotrimers it will still not render the compounds useless, but it will be critically important for potential users to know.

We thank the reviewer for the constructive evaluation of our work and for noting that the revised manuscript addressed the majority of the prior suggestions. We also respond here to the remaining question regarding potential effects of the β-arrestin modulators on Gi signaling. We agree that this is an important point to clarify in light of the compounds effect and reductions in maximal Gi activation observed in the TRUPATH assay at M2R. To address this, we undertook a number of additional complementary cellular and biophysical experiments. As suggested by the reviewer, we extended our analysis using the same TRUPATH platform to evaluate the effect of the modulators on Gi activation through stimulation of the dopamine D2 receptor (D2R), in addition to M2R. Interestingly, a similar inhibition of Gi activation was noted at D2R, indicating that the effect was not confined to a single receptor context (Extended Data Fig. 4f). We next turned to an alternative assay format to assess Gi activation under conditions that more closely reflect endogenous Gi signaling in cells. Using a Rap1GAP-based NanoBiT luciferase complementation reporter to monitor endogenous Gαi activation, we found that β2AR receptor agonist-driven endogenous Gi signaling was not affected by any of the β-arrestin modulators (Extended Data Fig. 4g).

Consistent with this endogenous Gi activation data, we next examined agonist-induced recruitment of mini-Gi to CCR7 and β2AR using a NanoBiT format. Under these conditions, mini-Gi recruitment remained comparable between modulator-treated and control samples and was not detectably perturbed by the β-arrestin modulators (Extended Data Fig. 4h,i), indicating that receptor–G protein coupling mediated through engagement of the Gαi α5 helix with the receptor core was not affect and is preserved.

As the reviewer also noted that our initial ITC experiments were performed with the Gαi subunit, we therefore repeated these measurements using the purified full Gi heterotrimer (Gαiβγ). We observed no detectable or saturable binding with the purified heterotrimer (Extended Data Fig. 4j,k), arguing against direct physical engagement of the Gi complex. Note that the tracings obtained with the compounds do not differ significantly from the buffer/DMSO control. These tracings can be compared with the ITC profiles for compound binding to β-arrestins shown in main Fig. 1h and Extended Data Fig. 3.

In summary, we have examined the effects of the new inhibitors in three assays that report on Gi activity using 4 receptors and have also performed new ITC experiments with heterotrimeric Gi. Taken all these together, these complementary analyses indicate that although effects of the modulators on Gi responses were observed in the trupath assay (while no effects were observed across the other three G protein classes tested), these Gi-related observations were not recapitulated in complementary approaches assessing endogenous Gi activation, receptor–Gi coupling, or direct biophysical interaction of the compounds with the purified Gi heterotrimer. We have therefore revised the manuscript to present these findings in a manner fully supported by the data. All new data and corresponding text updates are included in the revised manuscript (Main text, page 6, lines 125–148; Extended Data Fig. 4). We have also added the following cautionary note to this text: “They do, however, suggest that G_i-related effects observed in the trupath format should be interpreted with caution in the context of these modulators.”

Referee #2 (Remarks to the Author):

The authors have substantially improved a manuscript that was already very good. I am satisfied with their responses to my comments and recommend publication of the manuscript in its current form.

Referee #3 (Remarks to the Author):

The authors have provided a thoughtful, comprehensive, and high-quality revision. All major scientific concerns have been fully addressed with new data, and the manuscript now presents a compelling, rigorous, and field-advancing study.

I fully support publication of this manuscript in its current revised form.

It represents a significant contribution to GPCR signaling research and introduces the first well-validated small-molecule probes that directly modulate β-arrestin function—a toolset the field has long needed.
